# Randomized, placebo‐controlled, double‐blind phase I trial of co‐administered pyronaridine and piperaquine in healthy adults of sub‐Saharan origin

**DOI:** 10.1111/cts.13738

**Published:** 2024-04-09

**Authors:** Andrea Kuemmerle, Denis Gossen, Annick Janin, Andrew Stokes, Nada Abla, Maja Szramowska, Ulrike Lorch, Myriam El Gaaloul, Isabelle Borghini‐Fuhrer, Stephan Chalon

**Affiliations:** ^1^ Medicines for Malaria Venture Geneva Switzerland; ^2^ Mangareva SRL Kraainem Belgium; ^3^ AKJ Consulting Divonne France; ^4^ Richmond Pharmacology Ltd. London UK; ^5^ PharmaKinetic Ltd. Quorn UK

## Abstract

Drug resistance to sulfadoxine‐pyrimethamine and amodiaquine threatens the efficacy of malaria chemoprevention interventions in children and pregnant women. Combining pyronaridine (PYR) and piperaquine (PQP), both components of approved antimalarial therapies, has the potential to protect vulnerable populations from severe malaria. This randomized, double‐blind, placebo‐controlled (double‐dummy), parallel‐group, single site phase I study in healthy adult males or females of Black sub‐Saharan African ancestry investigated the safety, tolerability, and pharmacokinetics of PYR + PQP (*n* = 15), PYR + placebo (*n* = 8), PQP + placebo (*n* = 8), and double placebo (*n* = 6) administered orally once daily for 3 days at the registered dose for the treatment of uncomplicated malaria. All participants completed the study. Forty‐five adverse events were reported in 26 participants, most (41/45) were mild/moderate in severity, with no serious adverse events, deaths, or study withdrawals. Adverse events were reported in 66.7% (10/15) of participants administered PYR + PQP, 87.5% (7/8) with PYR + placebo, 50.0% (4/8) with PQP + placebo, and 83.3% (5/6) with placebo. For PYR containing regimens, five of 23 participants had asymptomatic transient increases in alanine and/or aspartate aminotransferase. With PQP containing regimens, four of 23 participants had mild Fridericia‐corrected QT interval prolongation. Liver enzyme elevations and prolonged QTc interval were consistent with observations for PYR‐artesunate and dihydroartemisinin‐PQP, respectively, administered to healthy adults and malaria patients. Increases in PYR and PQP exposures were observed following co‐administration versus placebo, with substantial interparticipant variability. The findings suggest that PYR + PQP may have potential in chemoprevention strategies. Further studies are needed in the target populations to assess chemoprotective efficacy and define the benefit–risk profile, with special considerations regarding hepatic and cardiac safety.

**Study Highlights**

**WHAT IS THE CURRENT KNOWLEDGE ON THE TOPIC?**

Resistance to current therapies threatens the effectiveness of chemoprevention in populations at risk. The combination of pyronaridine (PYR) and piperaquine (PQP), both components of approved antimalarial therapies, has potential for protecting populations vulnerable to severe malaria.

**WHAT QUESTION DID THIS STUDY ADDRESS?**

This placebo‐controlled phase I study in adults of sub‐Saharan ancestry investigated the safety, tolerability, and pharmacokinetics of PYR and PQP co‐administration at the registered doses and 3‐day dosing regimen used for malaria treatment.

**WHAT DOES THIS STUDY ADD TO OUR KNOWLEDGE?**

The safety and tolerability findings of PYR and PQP following 3‐day dosing in the fasted state in healthy participants of sub‐Saharan ancestry were consistent with previously observed safety/tolerability profiles in healthy adults and patients with malaria who received PYR‐artesunate or dihydroartemisinin‐PQP. The PYR + PQP co‐administration increased the exposure to both PYR and PQP, although with substantial interparticipant variability.

**HOW MIGHT THIS CHANGE CLINICAL PHARMACOLOGY OR TRANSLATIONAL SCIENCE?**

Further studies are needed in the target populations to assess chemopreventive efficacy and define the benefit–risk profile, with special considerations regarding hepatic and cardiac safety.

## INTRODUCTION

There were an estimated 247 million malaria cases across 84 endemic countries in 2021.^1^ Malaria chemoprevention uses antimalarial medicines administered at predefined times, irrespective of infection status, to treat existing infections and prevent new infections in people living in endemic areas.^2^ Strong evidence supports chemoprevention effectiveness in reducing malaria prevalence in selected high‐risk populations. The World Health Organization (WHO) recommends intermittent preventive treatment of malaria in pregnancy (IPTp), perennial malaria chemoprevention (PMC) in children up to 24 months old, and seasonal malaria chemoprevention (SMC) for children aged 3–59 months in areas of highly seasonal malaria transmission.^2^ These interventions are currently dependent upon sulfadoxine‐pyrimethamine (SP) for PMC and IPTp, and SP plus amodiaquine (AQ) for SMC, with doses given at least 1 month apart. Treatment regimens are affordable and well‐tolerated but the development and spread of *P. falciparum* strains resistant to SP and AQ threatens efficacy.^3^, ^4^, ^5^, ^6^

Protecting at‐risk populations requires new chemoprevention drugs. One approach is to re‐combine approved antimalarials that have appropriate pharmacokinetics (PKs), safety, and tolerability. A combination of pyronaridine (PYR) and piperaquine (PQP) may have potential for this purpose with a particular interest for IPTp and SMC strategies in sub‐Saharan Africa. PYR and PQP are components of approved fixed‐dose combinations with artesunate (PYR‐AS; Pyramax) and dihydroartemisinin (DHA‐PQP; Eurartesim). Extensive clinical trials demonstrated the efficacy, positive benefit–risk balance, and tolerability for uncomplicated malaria in adults and children and PYR‐AS and DHA‐PQP are approved by the European Medicines Agency and numerous malaria endemic counties. Both drugs are WHO prequalified and included in the WHO guidelines for malaria and the WHO list of essential medicines.^2^, ^7^ Both PYR and PQP have long half‐lives, compatible with monthly administration.^8^, ^9^, ^10^, ^11^ DHA‐PQP has demonstrated efficacy in malaria chemoprevention in children and pregnant women.^12^, ^13^, ^14^ However, DHA‐PQP is also a frontline treatment for malaria in Africa, and prolonged and widespread use for chemoprevention could increase the risk of drug resistance selection. Because PYR and PQP both have long half‐lives and exhibit different mechanisms of action, their use in combination may reduce the risk for drug resistance selection and spread.^15^, ^16^ As regard to the potential use in pregnant women, neither drug has shown any teratogenic liabilities,^17^, ^18^ with PYR‐AS being investigated in this population,^19^ and DHA‐PQP characterized in IPTp.^13^, ^14^

Malaria chemoprevention is deployed to protect infants, children, and pregnant women without clinical malaria, and pharmacological agents must have a low safety risk and favorable tolerability profile. Gastrointestinal adverse events are reported for PYR and PQP but are usually mild/moderate in severity.^20^, ^21^, ^22^, ^23^ For PYR, the main safety signal is transient asymptomatic elevations in alanine aminotransferase (ALT) and/or aspartate aminotransferase (AST), reported in patients with malaria and healthy volunteers.^20^, ^24^, ^25^, ^26^ Whereas increased ALT/AST is rare with PQP, the drug is known to prolong Fridericia‐corrected QT interval (QTcF). Because of a clinically relevant food effect that increases systemic exposure, PQP should be administered in a fasted state to mitigate QTcF prolongation.^27^, ^28^ PQP‐induced QTcF prolongation does not appear to translate into an increased risk for torsade de pointes if taken in the fasted state when other QT prolongation risk factors are controlled.^29^, ^30^, ^31^ Conversely, no clinically concerning QTcF prolongation is expected with PYR and there is no clinically relevant food effect with PYR administration.^29^ Overall, there are no prohibitive overlaps in the PYR and PQP safety/tolerability profiles preventing further evaluation of this combination for malaria chemoprevention.^17^, ^18^ Furthermore, physiologically‐based pharmacokinetic (PBPK) modeling of potential PK drug–drug interactions (DDIs) using the known properties of the two compounds predicted no significant interaction at therapeutic drug exposures (N. Abla, personal communication).^32^ This phase I study in healthy adults investigated the safety, tolerability, and PKs of PYR and PQP following co‐administration and when administered alone in comparison to placebo.

## METHODS

### Study design

**FIGURE 1:**
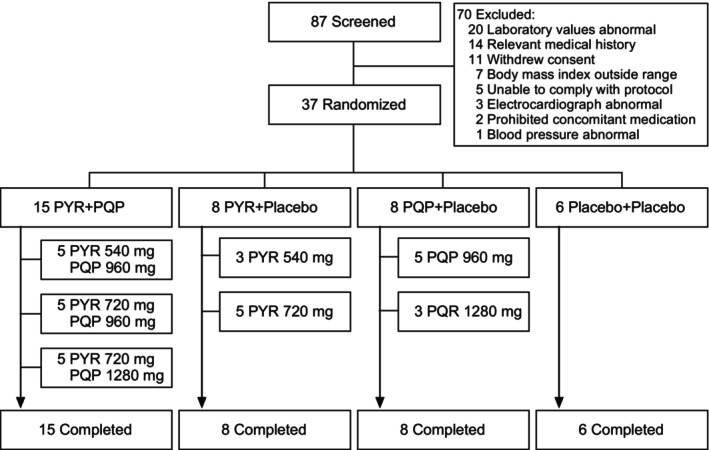
Participant disposition. PYR, pyronaridine; PQP, piperaquine.

This single‐center, randomized, double‐blind, placebo‐controlled (double dummy), parallel‐group, study was conducted at Richmond Pharmacology Ltd., London, between February 14, 2022, and May 31, 2022. The study is registered at ClinicalTrials.gov (NCT05160363) and EUDRACT (2021–005698‐21). The protocol is provided with this article (Protocol S1). Study drugs were PYR 180 mg tablets (Shin Poong Pharm. Co. Ltd., Ansan, Korea), PQP 320 mg tablets (Piramal Pharma Ltd., Mumbai, India), and matched placebos supplied by the manufacturers. Participants were randomized in the ratio 2:1:1:1 using a computer‐generated randomization schedule to PYR + PQP (*n* = 15), PYR + placebo (*n* = 8), PQP + placebo (*n* = 8), and double placebo (*n* = 6). Drug doses were based on bodyweight at screening as per labels for the approved antimalarial drugs PYR‐AS (Pyramax) and DHA‐PQP (Eurartesim): PYR 540 mg (50–<65 kg) and 720 mg (≥65 kg); PQP 960 mg (50–<75 kg) and 1280 mg (≥75 kg), with randomization stratified within each treatment arm to ensure at least two participants in each dose group (Figure 1). Examining a combination of the known efficacious doses of PYR and PQP allowed comparison with existing data. A placebo group was included as a control and to permit concentration–QT interval modeling for individual drugs and the combination (published separately). The study sponsor, clinical unit staff, and participants were blinded to treatment allocation.

### Participants

Eligible participants were healthy men or women self‐reported as Black ethnicity and sub‐Saharan ancestry, aged greater than or equal to 18 to less than or equal to 45 years, bodyweight greater than or equal to 50 kg, body mass index 18–28 kg/m^2^ without clinically relevant illness or surgery within 4 weeks prior to dosing, without abnormalities in baseline electrocardiograph (ECG), safety laboratory, vital signs, or physical examination, or a history of neurological, endocrine, cardiovascular, respiratory, hematological, immunological, psychiatric, gastrointestinal, renal, hepatic, or metabolic disease. Women of childbearing potential and men were required to use highly effective contraception for 110 days from first drug administration (Protocol S1). Pregnant or breastfeeding women and men whose partners were pregnant, breastfeeding, or due to give birth during the trial period were excluded. Additional exclusion criteria are detailed in the protocol (Protocol S1).

### Procedures

Participants were screened between day −22 and day −2 and admitted on day −1. Screening assessments included physical examination, medical history, safety laboratory tests, inclusion/exclusion criteria assessment, triplicate 12‐lead ECGs conducted after resting supine for 10 mins, and a 24‐h Holter ECG.

Participants had to refrain from tobacco, alcohol, caffeine or xanthine containing products, energy drinks or drinks containing taurine or glucuronolactone, strenuous physical activity, or excessive ultraviolet radiation exposure. No prior concomitant medication was allowed except hormonal contraception. The administration of paracetamol (≤1000 mg PRN) was allowed.

Participants received a single dose of study drugs in the morning on days 1, 2, and 3. Drugs were administered after a fast of at least 3 h prioir to dosing and with a fast of at least 4 h after dosing as PQP must be given in the fasted state to reduce the risk of clinically significant QTc prolongation. Study staff supervised all doses.

A sentinel dosing strategy included four initial participants from the PYR + PQP arm, and two from each of the other arms. Participants in the sentinel group were discharged on day 7, with outpatient assessments on days 8, 15, and 22. Blinded safety data to day 15 were reviewed by a Safety Review Committee prior to dosing the remaining participants. Non‐sentinel group participants could be discharged on day 5, with outpatient assessments on days 6, 8, 15, and 22. Final assessments were done on day 30 (±1 day, Protocol S1).

Blood samples for PK assessments were obtained frequently following each drug administration, then at 72, 120, 168, 336, and 504 h post first dose (Protocol S1). PYR and PQP concentrations were determined by Swiss Bioquant AG (Reinach, Switzerland) using validated methods. The limit of quantification (LOQ) was 1.00 ng/mL in whole blood for PYR and 1.00 ng/mL in plasma for PQP.

Adverse events were categorized using the Medical Dictionary for Regulatory Activities (version 25.0) and severity graded using predetermined standardized criteria for phase I studies, with relationship to study medication assessed by the investigator (Protocol S1). Predefined adverse events of special interest (AESI) were ALT or AST greater than three times the upper limit of normal (ULN), uncorrected QT interval greater than 500 msec, second or third AV block, bundle branch block, any arrhythmia, decrease in hemoglobin greater than 25% from baseline, or less than 10 g/dL, platelet count greater than 25% from baseline, or less than 80 × 10^9^/L, or a clinically important decrease in neutrophil count from baseline.

Blood samples for clinical chemistry and hematology were taken, vital signs recorded, and digitalized triplicate ECGs performed predose on all in‐patient days and days 15, 22, and 30. Continuous 12‐lead telemetry was done from at least 1 h predose on day 1 to 24 h postdose on day 3. ECGs of a given participant were over‐read by the same cardiologist blinded to time and treatment allocation. Urinalysis samples were obtained predose and on days 1, 3, 5, 7 (sentinel group only), 8, 15, and 30.

### End points

The primary safety end points were the incidence, severity, and relationship to drug administration of treatment emergent adverse events, the proportion of participants with clinically relevant changes in laboratory safety tests and vital signs, and the proportion of participants with morphological and/or rhythm abnormalities or clinically important changes to ECG time intervals (PR, QRS, QT, and QTcF) against baseline.

Secondary end points were PK parameters on days 1 and 3, derived by noncompartmental analysis methods using individual concentration data for PYR in blood and PQP in plasma, and actual time for drug administration and blood sampling (Phoenix WinNonlin version 8.3; Pharsight Corporation). These were maximum observed concentration (*C*_max_), time to reach maximum concentration (*T*_max_), terminal elimination half‐life (*t*_1/2_), area under the concentration time curve (AUC) from time 0 to last detectable concentration (AUC_0−t_), AUC from time 0 extrapolated to infinity (AUC_0−inf_), percentage of AUC due to extrapolation (%AUC_extrap_, i.e., AUC_t−inf_/AUC_0−inf_), apparent total clearance, apparent volume of distribution during the terminal phase, and terminal elimination rate constant. AUC_0‐t_ and AUC_0‐inf_ were calculated using the linear/log trapezoidal method, applying the linear trapezoidal rule up to *C*_max_ and the log trapezoidal rule for the remainder of the curve. Samples below the LOQ prior to the first quantifiable concentration were set to zero and after the first quantifiable concentration were set to “missing” and omitted from the analysis. Other PK parameters were calculated according to standard equations.

### Statistics

For this exploratory trial, there was no formal sample size calculation. Data were analyzed according to a prospective statistical plan using SAS (version 7.1; SAS Institute Inc.). Safety was analyzed in the safety population including all randomized participants who received at least one dose of study drug. The PKs were analyzed in participants in the safety population with sufficient blood samples for calculation of at least one PK parameter. ECGs were analyzed in those participants in the safety population with at least one valid predose and one postdose ECG assessment based on two evaluable replicates. Safety outcomes were reported using descriptive statistics. ECG analyses compared observations prior to drug administration versus postdose timepoints using descriptive statistics. QT was corrected using Fridericia's method unless there was an effect on heart rate exceeding 10 beats/min. PK data analysis used descriptive statistics, including calculation of geometric mean and inter‐participant co‐efficient of variation.

### Ethics statement

Informed written consent was obtained from all participants. South Central–Berkshire B Ethics Committee granted ethical approval and the protocol was approved by the United Kingdom Medicines and Healthcare Products Regulatory Agency. The study complied with Good Clinical Practice standards of the International Conference on Harmonization on (Topic E6), the Declaration of Helsinki and applicable United Kingdom law.

## RESULTS

### Participants

Of 87 volunteers screened, 37 (42.5%) were enrolled and randomized (Figure 1). All participants completed the study and were included in the safety, PK, and ECG analyses (Figure 1). Overall mean (SD) age was 27.8 (6.0) years, and bodyweight 70.6 (11.1) kg (Table S1). Women comprised 56.8% (21/37) of the population (aged 18–38 years). All women were of child‐bearing potential; 13 practiced abstinence and six took oral contraception (2 received PYR + PQP 540/960 mg, 2 PYR + PQP 720/960 mg, one PYR 720 mg, and one PQP 1280 mg). Adherence to medication was 100% and no participant vomited after dosing.

### Safety

Forty‐five adverse events were reported in 26 participants, 86.7% (39/45) were mild, 4.4% (2/45) moderate, and 8.9% (4/45) severe. Gastrointestinal adverse events were most common overall, occurring in 32.4% (12/37) of participants across all study groups (Figure S1). There were no serious adverse events or deaths and no adverse events leading to study withdrawal.

Adverse events were reported in 66.7% (10/15) of the participants administered PYR + PQP, 87.5% (7/8) with PYR + placebo, 50.0% (4/8) with PQP + placebo, and 83.3% (5/6) with placebo, with no trends based on drug doses (Figure S1). The most common adverse events were hypertransaminasemia with PYR + PQP (3/15) and PYR + placebo (2/8), and nausea (2/8), and upper respiratory infection (2/8) with PQP + placebo (Figure S1).

**FIGURE 2:**
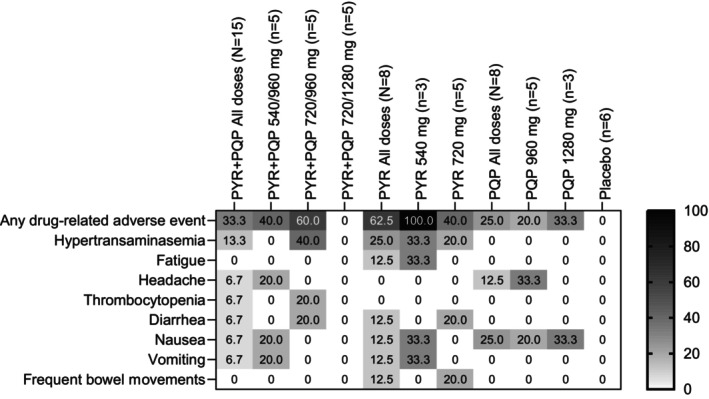
Frequency of treatment‐emergent drug‐related adverse events following administration of PYR, PQP, or co‐administration of PYR + PQP. Values are the percentage of participants experiencing the adverse event. PYR, pyronaridine; PQP, piperaquine.

Treatment‐related adverse events were reported in 33.3% (3/15) of participants administered PYR + PQP, 62.5% (5/8) with PYR + placebo, 25.0% (2/8) with PQP + placebo, and 0% (0/6) with placebo, with no obvious relationship to dose (Figure 2). The most common treatment‐related adverse events were hypertransaminasemia with PYR + PQP (2/15) and PYR + placebo (2/8), and nausea with PQP + placebo (2/8).

There were four severe adverse events in four participants, all were asymptomatic increases in liver transaminases: two in the PYR + PQP 720/960 mg group, one with 540 mg PYR + placebo, and one with 720 mg PYR + placebo (Figure 2, Table 1).

**TABLE 1:** Adverse events of special interest.

Age (years, sex)	Treatment group	Adverse event	Onset	Relationship to drug	Severity	Outcome
33 male	PYR 720 mg	Hypertransaminasemia ALT >5×ULN; AST >3×ULN	Day 4 (24 h postdose)	Related	Severe	Resolved
34 male	PYR + PQP 720/960 mg	Hypertransaminasemia ALT >3×ULN	Day 4 (24 h postdose)	Related	Moderate	Resolved
20 female	PYR + PQP 720/960 mg	Hypertransaminasemia ALT >5×ULN; AST >3×ULN	Day 25 (530 h postdose)	Not related^a^	Severe	Not resolved
30 female	PYR 540 mg	Hypertransaminasemia ALT >20×ULN; AST >20×ULN	Day 3 (24 h postdose)	Related	Severe	Resolved
36 female	PYR + PQP 720/960 mg	Hypertransaminasemia ALT >5×ULN; AST >5×ULN	Day 4 (24 h postdose)	Related	Severe	Resolved
		Thrombocytopenia	Day 8 (144 h postdose)	Related	Moderate	Resolved

Abbreviations: ALT, alanine aminotransferase; AST, aspartate aminotransferase; PYR, pyronaridine; PQP, piperaquine; ULN, upper limit of normal.

^a^ Attributed to documented recent Epstein–Barr infection.

Six AESI occurred in five participants. Five cases had increased liver aminotransferases and one participant (PYR + PQP 720/960 mg) also showed thrombocytopenia, reported as a moderate adverse event (Table 1). One case was attributed to Epstein–Barr virus infection, confirmed by viral serology with ALT/AST elevation starting on day 12, peaking 27 days after the last dose; the time to full recovery was not assessable owing to missing data. The remaining four AESI/increased liver aminotransferase cases (3 severe and 1 moderate) were deemed drug‐related, with ALT/AST elevations observed with a very similar time course, with peak elevation 1 or 2 days after the last dose and reaching ALT/AST values above 20 times the ULN in one study participant, returning to normal range between days 15 and 30, with no apparent relationship to drug exposure (Figure S2). There were no potential Hy's Law cases, as hypertransaminasemia was not concomitant with elevated bilirubin greater than two times the ULN (Figure S3). Safety narratives for these participants are included in the supplementary materials (Narratives S1).

Excepting the above‐mentioned AESI, there were no clinically relevant changes in hematology, coagulation, biochemistry, or urinalysis findings. Low values for lymphocytes were observed for 12 of 15 participants receiving PYR + PQP, eight of eight with PYR + placebo, one of eight with PQP + placebo, and three of six with placebo, although these findings were not clinically important (Table S2). In the PYR containing arms, all the low lymphocyte values followed a similar time‐course, starting at day 4–5, and returning to normal by day 15 (Table S2). Increases in eosinophils percentage were also observed in seven of 15 participants with PYR + PQP, three of eight with PYR + placebo, three of eight with PQP + placebo, and one of six with placebo. There were also sporadic occurrences of low eosinophils percentage. The changes were not clinically important and did not appear to be related to dose (Table S3).

There were no clinically important changes in vital signs or physical examination (Figure S4). There were no changes in QTcF from baseline greater than 60 msec and no QTcF values greater than 480 msec, including in the PQP containing arms (Table S4). For observations after the first dose, changes from baseline QTcF greater than 30 and less than 60 msec occurred for 8.3% (45/540) of observations with PYR + PQP, 4.9% (14/288) with PQP + placebo, 1.0% (3/288) with PYR + placebo, and 0.5% (1/216) with placebo (Table S4). The greatest increase in QTcF from baseline was 43.9 msec (PYR + PQP 720/960 mg) at day 3, for 3 h postdose. QTcF values greater than 450 and less than 480 msec occurred for 0.4% (2/540) of observations with PYR + PQP, 5.6% (16/288) with PQP + placebo, with no occurrences with PYR + placebo or placebo (Table S4). The highest QTcF value was 469 msec, observed at the first ECG on day 3 at 3 h postdose (PQP 960 mg) with second and third QTcF values of 440 and 443 msec, respectively.

### Pharmacokinetics

Systemic concentration data were available for all 37 participants. Note that all comparisons between groups should be interpreted cautiously owing to the parallel study design.

**FIGURE 3:**
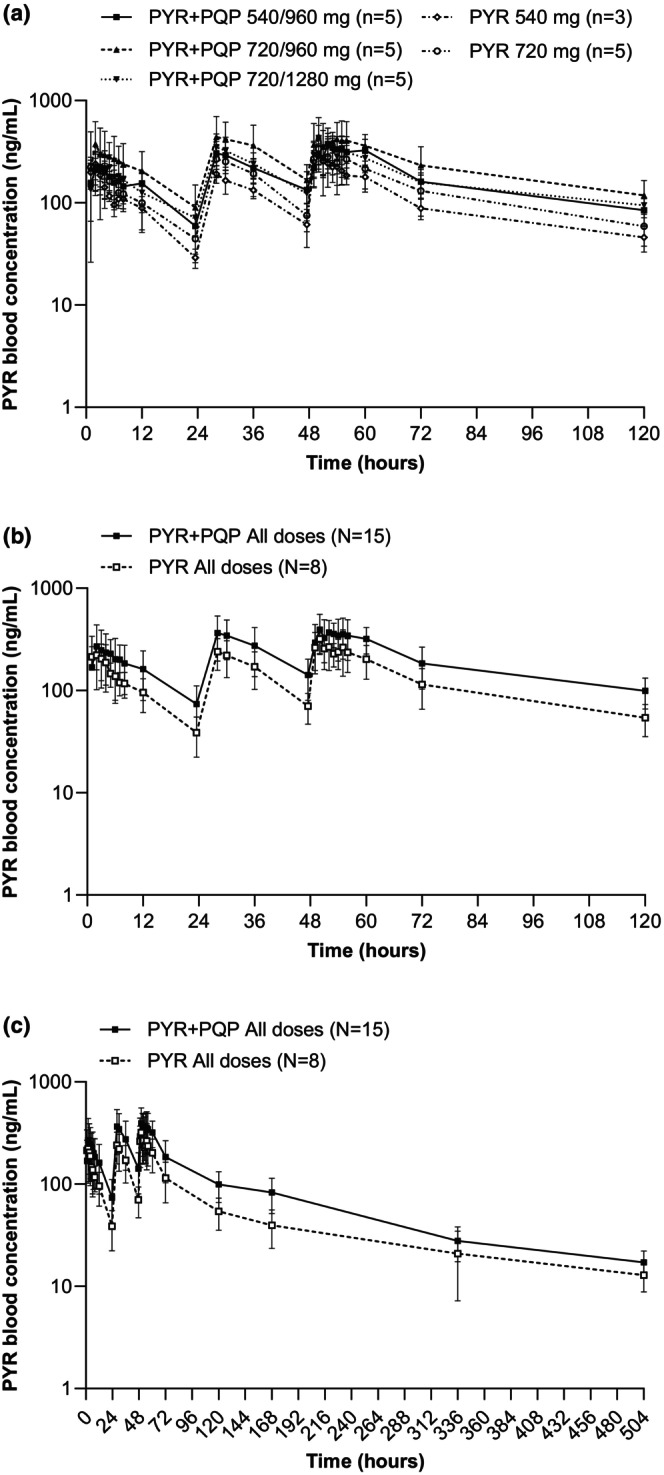
Blood concentration–time profiles for PYR following co‐administration with PQP or placebo. (a) Individual dosing groups over 120 h. (b) Combined PYR + PQP or PQP dosing groups over 120 h. (c) Combined PYR + PQP or PQP dosing groups over 504 h. PYR, pyronaridine; PQP, piperaquine.

PYR blood concentrations declined from their peak in a multiphasic manner following PYR + PQP or PYR + placebo, with drug quantifiable until the last sampling timepoint for all participants (Figure 3). PYR PK parameters following administration of PYR + PQP or PYR + placebo are shown in Table 2. PYR geometric mean *C*_max_ was lower on day 2 versus days 1 and 3 in the PYR + placebo group. In contrast, participants dosed with PYR + PQP had increasing *C*_max_ values from day 1 to day 3. In addition, day 3 *C*_max_ was 1.3‐fold higher (432.1 ng/mL) following PYR + PQP than with PYR + placebo (321.4 ng/mL; Table S5). PYR geometric mean day 3 AUC_0−t_ was multiplied 1.6‐fold with PYR + PQP co‐administration (30,291.5 ng∙h/mL) versus PYR + placebo (18,497.9 ng∙h/mL; Table S5). Day 3 PYR median *T*_max_ was similar for PYR + PQP co‐administration (3.00 h) and PYR + placebo (2.02 h) indicating that PQP co‐administration had no important effect on PYR absorption rate. Day 3 PYR *t*_1/2_ was similar following either PYR + PQP (147.9 h) or PYR + placebo (198.1 h).

**TABLE 2:** Pharmacokinetic parameters for PYR in blood following coadministration with PQP or placebo.

Parameter	Time	PYR + PQP dose groups (mg)				PYR + placebo dose groups (mg)		
		All doses (*N* = 15)	540/960 (*n* = 5)	720/960 (*n* = 5)	720/1280 (*n* = 5)	All doses (*N* = 8)	540 (*n* = 3)	720 (*n* = 5)
*C*_max_ (ng/mL)	Day 1	298.1 (44.9)	258.5 (19.5)	345.8 (60.8)	296.3 (13.7)	258.7 (25.4)	262.9 (12.8)	256.2 (32.3)
	Day 2	347.6 (45.3)	292.7 (30.4)	428.0 (52.1)	335.2 (34.4)	228.1 (35.7)	189.7 (19.5)	254.8 (35.6)
	Day 3	432.1 (32.1)	396.2 (35.2)	475.2 (36.1)	428.6 (27.8)	321.4 (30.2)	300.3 (26.0)	334.8 (33.4)
*T*_max_ (h)	Day 1	2.03 (1.0–5.0)	3.00 (1.0–4.0)	2.03 (2.0–5.0)	2.03 (1.1–5.0)	2.00 (1.0–4.0)	2.00 (1.0–2.0)	2.00 (1.0–4.0)
	Day 2	4.00 (4.0–12.0)	4.00 (4.0–4.0)	6.00 (4.0–12.0)	4.10 (4.0–6.0)	4.09 (4.0–6.0)	4.10 (4.0–4.1)	4.08 (4.1–6.0)
	Day 3	3.00 (1.0–12.0)	4.05 (2.0–12.0)	3.00 (2.0–5.0)	2.00 (1.0–3.0)	2.02 (1.0–7.0)	2.02 (2.0–3.0)	2.02 (1.0–7.0)
*t*_1/2_ (h)	Day 1	7.61 (53.0)^d^	10.01 (7.9)^e^	9.24 (24.6)^e^	5.58 (99.5)^f^	8.86 (51.2)^b^	8.13 (—)^g^	9.11 (58.4)^f^
	Day 3	147.86 (16.0)	154.32 (19.0)	141.75 (19.5)	147.76 (10.5)	198.13 (18.0)	199.96 (13.0)	196.77 (23.2)
AUC_0−t_ (ng∙h/mL)	Day 1	2169.7 (50.1)	1922.1 (20.3)	2654.1 (62.5)	2002.1 (24.1)	1682.5 (22.1)	1532.2 (8.9)	1779.7 (24.7)
	Day 2	3200.7 (44.9)	2761.7 (28.7)	3863.5 (53.6)	3073.0 (33.0)	2056.4 (36.6)	1669.5 (20.2)	2330.4 (35.2)
	Day 3	30291.5 (32.5)	26299.5 (26.9)	34482.0 (40.7)	30649.4 (19.2)	18497.9 (36.0)	16172.3 (22.8)	20050.8 (38.4)
AUC_0−24h_ (ng∙h/mL)	Day 1	3466.4 (51.4)^a^	3242.7 (24.7)^b^	4197.7 (59.0)^c^	2917.1 (45.8)^b^	2451.3 (26.6)	2234.9 (10.1)	2591.1 (30.3)
	Day 3	6790.2 (31.3)	6455.8 (26.2)	7875.2 (35.1)	6158.0 (27.5)	4541.0 (32.7)	4082.9 (21.0)	4840.2 (35.9)
AUC_0−168h_ (ng∙h/mL)	Day 1	5100.9 (59.5)^a^	5955.3 (64.1)^b^	5741.8 (56.6)^c^	3768.1 (67.6)^b^	3249.2 (39.4)	2844.0 (17.2)	3519.6 (43.0)
	Day 3	20789.0 (33.5)	18421.3 (28.3)	23991.5 (40.8)	20329.4 (21.3)	12133.8 (36.1)	10642.9 (24.4)	13126.7 (38.6)
AUC_0−inf_ (ng∙h/mL)	Day 1	3512.5 (57.2)^d^	3480.3 (29.1)^e^	4127.9 (47.8)^e^	3173.4 (88.0)^f^	2675.9 (35.1)^b^	2462.7 (—)^g^	2751.0 (40.2)^f^
	Day 3	33974.9 (31.0)	29428.9 (23.7)	38681.0 (39.3)	34451.0 (17.8)	23792.6 (30.2)	19346.1 (20.9)	27786.2 (25.7)
%AUC_extrap_ (%)	Day 1	33.7 (51.3)^d^	47.3 (7.4)^e^	47.7 (20.7)^e^	21.4 (102.4)^f^	34.7 (43.7)^b^	38.1 (—)^g^	33.7 (54.0)^f^
	Day 3	10.5 (26.2)	10.2 (29.8)	10.4 (31.6)	10.8 (22.7)	15.0 (29.9)^d^	16.2 (17.6)	14.2 (40.4)^b^
AUC_tau_ (ng∙h/mL)	Day 3	6790.2 (31.3)	6455.8 (26.2)	7875.2 (35.1)	6158.0 (27.5)	4541.0 (32.7)^d^	4082.9 (21.0)	4840.2 (35.9)^b^
CL/F (mL/h)	Day 1	188810.1 (54.3)^d^	155157.3 (29.1)^e^	174424.4 (47.8)^e^	226885.1 (60.1)^f^	250395.2 (39.8)^b^	219270.3 (—)^g^	261722.7 (44.3)^f^
	Day 3	96339.2 (29.8)	83645.6 (20.9)	91426.3 (33.0)	116921.7 (26.4)	142340.4 (39.1)	132257.5 (21.2)	148755.4 (45.7)
*λ*_*z*_ (1/h)	Day 1	0.091 (75.9)^d^	0.069 (7.8)^e^	0.075 (24.6)^e^	0.124 (73.6)^f^	0.078 (38.0)^b^	0.085 (—)^g^	0.076 (46.8)^f^
	Day 3	0.0047 (15.9)	0.0045 (19.2)	0.0049 (19.3)	0.0047 (9.9)	0.0035 (18.4)^d^	0.0035 (12.4)	0.0035 (23.5)^b^
*V*_z_/*F* (mL)	Day 1	2074048.6 (21.3)^d^	2240297.4 (21.5)^e^	2324597.4 (24.6)^e^	1825892.5 (19.9)^f^	3199294.1 (24.7)^b^	2571707.6 (—)^g^	3440844.4 (23.0)^f^
	Day 3	20550145.1 (36.4)	18622971.3 (29.0)	18696517.8 (48.6)	24924989.1 (30.5)	36882280.1^d^ (31.9)	38153688.4 (34.3)	35956605.6^b^ (35.3)

*Note*: Values are geometric mean (geometric mean coefficient of variation [CV%]) except for *T*_max_ which is median (range).

^a^ *N* = 13; ^b^ *N* = 4; ^c^ *N* = 5; ^d^ *N* = 7; ^e^ *N* = 2; ^f^ *N* = 3; ^g^ *N* = 1.

Abbreviations: %AUC_extrap_, percentage of AUC due to extrapolation (i.e., AUC_t−inf_/AUC_0−inf_); AUC, area under the blood concentration time curve; AUC_0−inf_, AUC from time 0 extrapolated to infinity; AUC_0−t_, AUC from time 0 to last detectable blood concentration; AUC_tau_, AUC from time zero until the end of the dosing interval; CL/F, apparent total plasma clearance; *C*_max_, maximum observed blood concentration; PQP, piperaquine; PYR, pyronaridine; *t*_1/2_, terminal elimination half‐life; *T*_max_, time to reach maximum blood concentration; *V*_*z*_/*F*, apparent volume of distribution during the terminal phase; *λ*_*z*_, terminal elimination rate constant.

**FIGURE 4:**
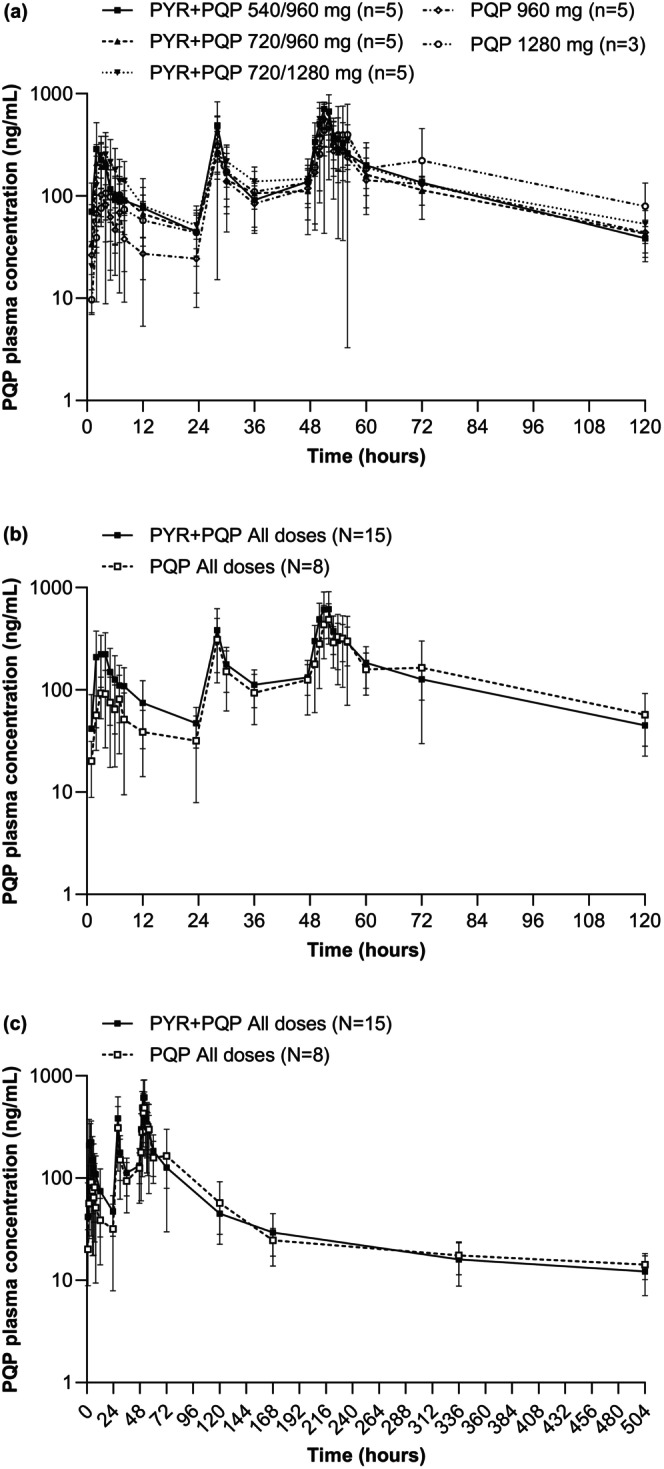
Plasma concentration–time profiles for PQP following co‐administration with PYR or placebo. (a) Individual dosing groups over 120 h. (b) Combined PYR + PQP or PQP dosing groups over 120 h. (c) Combined PYR + PQP or PQP dosing groups over 504 h. PYR, pyronaridine; PQP, piperaquine.

Following peak PQP plasma concentrations, levels declined in a multiphasic manner and remained quantifiable until the last sampling timepoint (Figure 4). PQP PK parameters following administration of PYR + PQP or PQP + placebo are shown in Table 3. Day 1 PQP geometric mean *C*_max_ was increased by 2.7‐fold following PYR + PQP (264.2 ng/mL) versus PQP + placebo (98.1 ng/mL; Table S6). On day 3, the PQP *C*_max_ was 642.6 ng/mL following PYR + PQP versus 473.3 ng/mL with PQP + placebo: a 1.4‐fold increase (Table S6). Following a single dose, PQP geometric mean AUC_0‐t_ following PYR + PQP was 1320.3 ng∙h/mL versus 606.1 ng∙h/mL with PQP + placebo, a 2.2‐fold increase (Table S6). Corresponding values for day 3 PQP AUC_0‐t_ were, however, similar (17,594.1 ng∙h/mL and 17,788.9 ng∙h/mL, respectively; Table S6). A post hoc analysis showed that intake of hormonal contraception did not explain the increased PQP exposure in the combination groups (data not shown). Day 3 PQP median *T*_max_ following PYR + PQP (3.03 h) or PQP + placebo (4.0 h) was similar, indicating no relevant effect of PYR coadministration on PQP absorption rate. On day 3, the PQP geometric mean *t*_1/2_ was 149.8 h following PYR + PQP and 351.9 h with PQP + placebo.

**TABLE 3:** Pharmacokinetic parameters for PQP in plasma following coadministration with PYR or placebo.

Parameter	Time	PYR + PQP dose groups (mg)				PQP + placebo dose groups (mg)		
		All doses (*N* = 15)	540/960 (*n* = 5)	720/960 (*n* = 5)	720/1280 (*n* = 5)	All doses (*N* = 8)	960 (*n* = 5)	1280 (*n* = 3)
*C*_max_ (ng/mL)	Day 1	264.2 (51.7)	290.0 (63.9)	247.1 (49.6)	257.5 (48.0)	98.1 (50.4)	97.9 (38.9)	98.5 (72.0)
	Day 2	317.6 (61.0)	373.9 (68.5)	244.3 (33.9)	350.9 (46.8)	259.2 (62.1)	279.1 (46.8)	229.1 (95.1)
	Day 3	642.6 (41.9)	629.8 (51.6)	596.1 (29.9)	706.8 (43.8)	473.3 (40.3)	519.0 (20.9)	405.8 (72.2)
*T*_max_ (h)	Day 1	3.00 (2.0–5.0)	2.00 (2.0–3.0)	3.00 (2.0–5.0)	3.00 (2.0–5.0)	3.01 (3.0–7.1)	3.00 (3.0–7.1)	3.02 (3.0–4.0)
	Day 2	4.00 (4.0–6.1)	4.00 (4.0–6.1)	4.02 (4.0–4.1)	4.07 (4.0–4.3)	4.02 (4.0–4.5)	4.02 (4.0–4.5)	4.02 (4.0–4.1)
	Day 3	3.03 (2.0–5.1)	3.00 (2.0–4.0)	3.03 (3.0–5.1)	4.00 (2.0–4.0)	4.00 (3.0–4.1)	4.00 (3.0–4.1)	4.00 (3.0–4.0)
*t*_1/2_ (h)	Day 1	5.45 (79.6)^a^	3.72 (−)^b^	4.34 (−)^b^	6.68 (79.3)^c^	4.37 (9.8)^d^	4.08 (−)^b^	4.68 (−)^b^
	Day 3	149.79 (58.2)^a^	215.34 (59.2)^d^	117.72 (−)^b^	117.54 (21.2)^d^	351.86 (67.8)^c^	351.86 (67.8)^c^	–^e^
AUC_0−t_ (ng∙h/mL)	Day 1	1320.3 (45.7)	1339.8 (44.0)	1208.0 (48.4)	1421.9 (50.3)	606.1 (55.8)	553.3 (44.3)	705.8 (69.5)
	Day 2	2032.5 (48.6)	2114.1 (60.4)	1702.4 (25.1)	2332.9 (43.6)	1651.7 (57.0)	1645.9 (43.2)	1661.3 (81.2)
	Day 3	17594.1 (26.2)	16517.2 (38.2)	16513.2 (20.9)	19968.1 (18.7)	17788.9 (43.5)	17224.1 (12.8)	18771.7 (66.8)
AUC_0−24h_ (ng∙h/mL)	Day 1	1767.1 (47.3)	1798.9 (57.0)	1604.6 (44.7)	1911.7 (46.5)	971.3 (47.6)^f^	850.6 (30.5)^g^	1266.7 (59.9)^d^
	Day 3	5665.8 (34.7)	5526.6 (52.2)	5435.3 (16.8)	6054.7 (26.8)	4844.1 (56.0)	4719.7 (21.1)	5058.8 (82.7)
AUC_0−168h_ (ng∙h/mL)	Day 1	2252.8 (173.6)	2785.5 (168.2)	1820.1 (42.6)	2255.2 (41.8)	1099.2 (46.5)^f^	985.3 (41.8)^g^	1368.1 (57.1)^d^
	Day 3	12635.7 (29.4)	11959.9 (43.4)	11787.6 (21.9)	14310.3 (21.6)	12461.8 (57.5)	11625.7 (14.2)	13991.1 (78.8)
AUC_0−inf_ (ng∙h/mL)	Day 1	2525.7 (22.5)^a^	1572.8 (−)^b^	2722.6 (−)^b^	2884.6 (5.1)^c^	1430.4 (51.8)^d^	973.9 (−)^b^	2100.7 (−)^b^
	Day 3	18174.3 (19.8)^a^	14719.5 (15.0)^d^	19464.5 (−)^b^	21683.7 (0.03)^d^	25595.5 (38.6)^c^	25595.5 (38.6)^c^	–^e^
%AUC_extrap_ (%)	Day 1	22.4 (66.3)^a^	12.2 (−)^b^	18.3 (−)^b^	29.5 (58.8)^c^	24.4 (28.2)^d^	19.9 (−)^b^	29.9 (−)^b^
	Day 3	8.6 (107.6)^a^	15.9 (91.3)^d^	4.9 (−)^b^	6.1 (8.0)^d^	22.0 (73.2)^c^	22.0 (73.2)^c^	–^e^
AUC_tau_ (ng∙h/mL)	Day 3	5665.8 (34.7)	5526.6 (52.2)	5435.3 (16.8)	6054.7 (26.8)	4844.1 (56.0)	4719.7 (21.1)	5058.8 (82.7)
CL/F (mL/h)	Day 1	451697.1 (20.7)^a^	610359.3 (−)^b^	352601.5 (−)^b^	443733.5 (5.2)^c^	774985.0 (33.4)^d^	985689.6 (−)^b^	609321.4 (−)^b^
	Day 3	186491.4 (46.3)	173706.0 (76.5)	176621.9 (15.8)	211406.0 (25.2)	220755.6 (50.8)	203404.9 (19.6)	253024.3 (66.2)
*λ*_*z*_ (1/h)	Day 1	0.127 (43.2)^a^	0.186 (−)^b^	0.160 (−)^b^	0.104 (62.7)^c^	0.159 (9.8)^d^	0.170 (−)^b^	0.148 (−)^b^
	Day 3	0.005 (36.0)^a^	0.003 (59.2)^d^	0.006 (−)^b^	0.006 (21.2)^d^	0.002 (76.4)^c^	0.002 (76.4)^c^	–^e^
*V*_*z*_/*F* (mL)	Day 1	3553144.3 (74.9)^a^	3275921.3 (−)^b^	2208576.5 (−)^b^	4277658.5 (77.3)^c^	4886112.6 (23.9)^d^	5796923.5 (−)^b^	4118408.1 (−)^b^
	Day 3	54383402.2 (114.3)^a^	90369125.2 (105.4)^d^	30801769.3 (−)^b^	43486857.2 (0.5)^d^	92525148.7 (60.8)^c^	92525148.7 (60.8)^c^	–^e^

*Note*: Values are geometric mean (geometric mean coefficient of variation [CV%]) except for *T*_max_ which is median (range).

^a^ *N* = 5; ^b^ *N* = 1; ^c^ *N* = 3; ^d^ *N* = 2; ^e^ *N* = 0; ^f^ *N* = 6; ^g^ *N* = 4.

Abbreviations: %AUC_extrap_, percentage of AUC due to extrapolation (i.e., AUC_t−inf_/AUC_0−inf_); AUC, area under the plasma concentration time curve; AUC_0−inf_, AUC from time 0 extrapolated to infinity; AUC_0−t_, AUC from time 0 to last detectable plasma concentration; AUC_tau_, AUC from time zero until the end of the dosing interval; CL/F apparent total plasma clearance; *C*_max_, maximum observed plasma concentration; PQP, piperaquine; PYR, pyronaridine; *t*_1/2_, terminal elimination half‐life; *T*_max_, time to reach maximum plasma concentration; *V*_*z*_/*F*, apparent volume of distribution during the terminal phase; *λ*_*z*_, terminal elimination rate constant.

## DISCUSSION

The safety, tolerability, and PKs of PYR and PQP co‐administration was assessed in healthy adult participants of sub‐Saharan origin. To our knowledge, this was the first clinical study conducted with this combination. Parallel groups of participants were administered with PYR‐PQP, PYR + placebo, PQP + placebo, and placebo + placebo (double‐dummy). All treatments were well‐tolerated, with no trends suggesting an increase in the frequency or severity of adverse events compared with the known safety profiles for PYR‐AS or DHA‐PQP in asymptomatic and symptomatic malaria patients.^12^, ^20^, ^21^, ^22^, ^23^, ^25^, ^27^, ^33^, ^34^

Increased exposures for PYR or PQP following co‐administration over 3 days were not anticipated. The DDI risk assessment performed prior to conducting the study was based on in vitro and clinical DDI data, indicating that PYR and PQP are both CYP3A4 substrates (~60% and 80% metabolized via this pathway, respectively), whereas only PQP is a moderate CYP3A4 inhibitor. PBPK simulations were conducted using models developed and validated for PYR and PQP with Simcyp (version 19; Certara).^32^ These predicted no clinically relevant change in PYR exposure when administered with PQP (i.e., <1.25‐fold increase in PYR exposure). Moreover, a clinical DDI study showed no significant increase in PYR exposure when PYR (associated with artesunate) was administered for 3 days together with the CYP3A4 inhibitor ritonavir over 17 days in healthy volunteers.^10^ Regarding PQP, in vitro or clinical data are not available to explain why PYR may affect its PK profile (e.g., CYP3A4 inhibition was not observed with PYR). Thus, no PBPK simulations were required. Six women administered PQP received concomitant hormonal contraceptives (levonorgestrel, etonogestrel, drospirenone, and ethinylestradiol). Some hormonal contraceptives are weak CYP3A inhibitors, and piperaquine is a CYP34A substrate.^35^, ^36^ However, in a DDI study of PQP and clarithromycin, which is a strong CYP34A inhibitor, PQP exposures were increased less than twofold.^35^, ^36^ Thus, it would not be expected that hormonal contraceptives would impact PQP PKs. Thus, the observed increase in PQP exposure mainly on day 1 *C*_max_ (2.7‐fold increase) when co‐administered with PYR suggests consideration of other mechanisms, such as at the level of PYR + PQP absorption. However, there was considerable interindividual variability observed in the PK parameters and the parallel group comparison was not formally designed to adequately quantify a clinically relevant PK DDI.

Transient and asymptomatic increases in ALT and AST considered drug‐related were observed in four individuals, receiving either PYR alone (*n* = 2) or combined with PQP (*n* = 2), with no significant trends associated with dose or between co‐administration and monotherapy. The observed ALT/AST elevations had a similar temporal pattern, consistent with previous reports for PYR‐AS in healthy adults, that is, ALT/AST elevations starting on day 3–7, peak values on days 8–15, with full resolution by day 21.^10^, ^11^, ^37^ In patients with malaria, the time course of ALT/AST increases following PYR‐AS typically occur on days 3–7 and normalize within 3 weeks.^20^, ^25^, ^26^, ^38^, ^39^, ^40^, ^41^

Three participants who received PYR had ALT increases greater than five times the ULN (including one >20× ULN). In previous studies in healthy adults, only one case of ALT greater than five times the ULN was reported following PYR‐AS, although AST/ALT values greater than three to five times the ULN were observed more frequently.^10^, ^11^, ^37^ There were no reports of possible Hy's law cases in the current study or previous studies in healthy adults.^10^, ^11^, ^37^ In a meta‐analysis of eight randomized clinical trials, ALT elevations greater than five times the ULN occurred in 1.1% (41/3785) of patients with uncomplicated malaria, with one reported case in which raised ALT occurred with raised bilirubin.^24^ However, to our knowledge, no study reported severe drug‐induced liver injury in patients with malaria with PYR‐AS.^24^

Examination of the hypertransaminasemia cases by two independent liver safety experts concluded that there was no clear difference between the observed increases in ALT/AST in this study versus previous reports with PYR‐AS in healthy White patients or patients with malaria.^10^, ^11^, ^20^, ^24^, ^25^, ^26^, ^37^, ^38^, ^39^, ^40^, ^41^ For this limited number of cases, there was no evidence that PQP co‐administration increased the incidence or severity of PYR‐induced ALT/AST elevations or that sub‐Saharan Africans were more at risk compared with previous reports of PYR‐induced ALT/AST elevations in healthy White subjects.^10^, ^37^ The experts concluded that the findings were consistent with a direct acute low‐level hepatotoxic reaction. Direct hepatotoxicity typically has a short latency, with an onset within 1 to 5 days after high therapeutic or supratherapeutic doses, without jaundice.^42^ The elevations resolve when the drug is stopped or the dose lowered but can resolve spontaneously.^42^ In patients with uncomplicated malaria, a repeat treatment study with PYR‐AS, in which patients received up to 11 3‐day treatment courses over 2 years, showed no evidence of a cumulative effect, with no detrimental effect on liver or other safety signals.^25^, ^26^ However, our findings suggest close monitoring of liver function tests with PYR + PQP co‐administration in future clinical development studies, a dosing interval of at least 1 month to allow for normalization of liver enzyme values, and clinical evaluation of shorter treatment durations (i.e., lower cumulative doses of PYR and PQP) to further assess the impact of the combination on the risk–benefit profile for malaria chemoprevention.

QTcF prolongation was expected and primarily observed in the two treatment groups receiving PQP with a higher incidence on day 3 indicating causality. The drug was administered in a fasted state as per the Eurartesim (DHA‐PQP) label, and none of the QTcF changes were clinically relevant, with no reports of absolute values greater than 480 msec or changes versus baseline greater than >60 msec. Changes in QTcF greater than 30 msec and less than 60 msec from baseline were more common with PYR + PQP than with PQP + placebo, but absolute changes greater than 450 and less than 480 were less frequent with the combination versus PQP + placebo. Concentration–QT modeling will be performed to investigate any impact of concomitant PYR administration on PQP‐induced QTc prolongation (reported separately).

Study limitations include a parallel design, weight‐based dosing, and a limited sample size. The study was not formally powered to quantify a clinically relevant DDI. Thus, the PK comparisons of monotherapies versus combined treatments should be interpreted cautiously. Although PQP has a bitter taste which cannot be masked, the end points were objective, and this was not thought to impact the trial outcomes.

In conclusion, PYR and PQP co‐administration for 3 days in the fasted state to healthy participants of sub‐Saharan ancestry at the registered doses used in combination with artemisinin derivatives for malaria treatment was not associated with unexpected safety or tolerability findings, despite an increased exposure of both drugs when compared in a parallel study design. This study supports further studies in the target populations to assess chemopreventive efficacy and to define the benefit–risk profile, with close monitoring of liver function tests, QTcF, and related safety signals.

## AUTHOR CONTRIBUTIONS

All authors wrote the manuscript. S.C., A.K., M.E.G., and I.B.‐F. designed the research. S.C., A.K., D.G., A.S., and U.L. performed the research. S.C., A.K., D.G., M.E.G, A.J., N.A., M.S., I.B.‐F., and U.L. analyzed the data.

## FUNDING INFORMATION

Funding for the conduct of this study and support for preparation of this manuscript was provided by Medicines for Malaria Venture. This work was supported, in whole or in part, by the Bill & Melinda Gates Foundation (grant number 19‐BMGF‐006). Under the grant conditions of the Foundation, a Creative Commons Attribution 4.0 Generic License has already been assigned to the Author Accepted Manuscript version that might arise from this submission.

## CONFLICT OF INTEREST STATEMENT

A.K. was employed at Medicines for Malaria Venture (MMV) when the study was designed and conducted and is a current employee of Novartis Institute for Biomedical Research, Basel, Switzerland; the content of this paper is the responsibility of the individual authors and neither the study nor this publication is associated with the Novartis Institute for Biomedical Research. D.G. is the owner and director of Mangareva SRL, which received financial support from MMV to review and interpret the study results. A.J. is the owner of AKJ Consulting, which received financial support from MMV to conduct the study. A.S. and U.L. are employees of Richmond Pharmacology Ltd., which received financial support from MMV to conduct the study. N.A., M.E.G., I.B.‐F., and S.C. are employees of MMV. M.S. is an employee of PharmaKinetic Ltd. which received financial support from MMV to perform the pharmacokinetic analysis.

## Supporting information


Data S1.

Figure S1.
