# Supplementary material for: Randomized, placebo‐controlled, double‐blind phase I trial of co‐administered pyronaridine and piperaquine in healthy adults of sub‐Saharan origin
Source: Clin Transl Sci. 2024 Apr 9;17(4):e13738. doi: 10.1111/cts.13738 (PMC11004265; doi:10.1111/cts.13738)
Supplement: Supplementary file 1 — Data S1. [file CTS-17-e13738-s001.pdf]

RICHMOND Pharmacology

MMV Medicines for Malaria Venture

CLINICAL STUDY PROTOCOL

Non-Substantial Amendment 2

Sponsor's Reference Number: MMV_SMC_21_01

Richmond Pharmacology Study Number: C21007

EudraCT Number: 2021-005698-21

**TITLE:**	A randomised, double-blind, placebo controlled, parallel group study in healthy adult volunteers to determine the tolerability and safety of pyronaridine (PYR) co-administered with piperaquine (PQP) under fasted conditions
**PHASE:**	Phase I
**DRUG:**	Pyronaridine and Piperaquine
**SPONSOR:**	Medicines for Malaria Venture (MMV)
**PRINCIPAL INVESTIGATOR:**	Dr Ulrike Lorch MD FRCA FFPM [redacted]
**STUDY CENTRE:**	Richmond Pharmacology Ltd. [redacted]
**Version and Date:**	Version 1.0<br>07 February 2022

Information in this protocol is confidential and should not be disclosed, other than to those directly involved in the execution or the ethical/regulatory review of the study, without written authorisation from MMV or its affiliates.

Amendment [Non-Substantial] 2 Signature Page

Protocol No: MMV_SMC_21_01:

This protocol amendment has been subjected to an internal MMV peer review.

A randomised, double-blind, placebo controlled, parallel group study in healthy adult volunteers to determine the tolerability and safety of pyronaridine (PYR) co-administered with piperaquine (PQP) under fasted conditions

I agree to the terms of this protocol amendment (non-substantial).

Sponsor's Signatories:

Stephan Chalon, MD, PhD

Signature: [redacted] **Date:** 08-Feb-2022

Andrea Kümmerle, PhD

Signature: [redacted] **Date:** 08-Feb-2022

Principal Investigator Signature:

I agree to the terms of this protocol amendment dated 07 Feb 2022. I will conduct the trial according to the procedures specified herein, and according to the principles of Good Clinical Practice and local regulations.

Principal Investigator:

Dr Ulrike Lorch MD FRCA FFPM

Signature: [redacted] **Date:** 08 Feb 2022

Reason for amendment

The purpose of this Non-Substantial Amendment 02 is to document changes made to the Clinical Study Protocol (Version 2.0, dated 10 JAN 2022).

The following changes have been incorporated:

1. An administrative error has been identified within the schedule of assessment table in the currently approved Protocol V2.0 dated 10 JAN 2022. Text describing the trial design in the protocol Section 3.0 states that "Non-sentinel group participants may be discharged on Day 5 and return for outpatient assessments on days 6, 8, 15 and 22, provided there are no clinically significant safety and tolerability signals and the SRC are in agreement" i.e., participants in the non-sentinel group may be discharged on Day 5, provided that the data from the sentinel group demonstrate acceptable safety and tolerability, and based on Safety Review Committee (SRC) decision. However, an 'X' mark on Day 7 column of the schedule of assessments, Table 5, indicated that non-sentinel group participants would be expected to attend for an outpatient visit on this day.

2. The corresponding 'X' on Day 7 for PK measurement was also added in error and is being removed to ensure consistency with the protocol design.

   These administrative errors in the schedule of assessments have been corrected to reflect the trial design.

3. An administrative error has been identified in the Synopsis Figure 1 and Section 3.0 Figure 2 – Trial Flow Chart. The outpatient visit for the non-sentinel group of participants was missing and has been added to the figure.

4. An administrative error was found in Section 4.4, Table 10 – Cohort and Trial Rules. In the first two rows, number of participants has been updated from ≤50 % to ≥ 50%.

The sponsor considers these changes as non-substantial as they are administrative changes only, correcting protocol drafting errors. Furthermore, safety of the trial participants is not affected as the safety procedures planned on Day 7 are being conducted on Day 6 and Day 8 as planned in the sentinel group.

The Participant Information Sheet and Informed Consent Form (PIS/ICF) is also being updated to align with the SOA in the updated clinical study protocol.

Sections of the protocol affected by the Non-Substantial Amendment:

1	Section 3.0 Trial Design– Table 5 – Schedule of Assessments and relevant Footnote 11
2	Synopsis – Figure 1 – Trial Flow Chart – The figure has been updated to reflect the trial design.
3	Section 3.0 - Figure 2 – Trial Flow Chart – The figure has been updated to reflect the trial design.
4	Section 4.4 – Adverse Reaction Rules – Table 10 – Cohort and Trial Rules – Text has been updated to correct a typographical error in the first two rows.

Persons who initiated the Amendment

MMV and Richmond Pharmacology Ltd

Sponsor:

Medicines for Malaria Venture (MMV)

Centres affected by the Amendment:

Richmond Pharmacology Ltd, UK

CLINICAL TRIAL PROTOCOL

Sponsor's Reference Number: MMV_SMC_21_01

Richmond Pharmacology Trial Number: C21007

EudraCT Number: 2021-005698-21

**TITLE:**	A randomised, double-blind, placebo controlled, parallel group study in healthy adult volunteers to determine the tolerability and safety of pyronaridine (PYR) co-administered with piperaquine (PQP) under fasted conditions
**PHASE:**	Phase I
**DRUGS:**	Pyronaridine and Piperaquine
**SPONSOR:**	Medicines for Malaria Venture (MMV)
**PRINCIPAL INVESTIGATOR:**	Dr Ulrike Lorch MD FRCA FFPM<br>Richmond Pharmacology Ltd.<br>[redacted] [redacted]<br>[redacted] [redacted]
**TRIAL CENTRE:**	Richmond Pharmacology Ltd.<br>[redacted]
**Protocol Version and Date:**	Version 3.0<br>07-FEB-2022

Information in this protocol is confidential and should not be disclosed, other than to those directly involved in the execution or the ethical/regulatory review of the trial, without written authorisation from MMV or its affiliates.

TABLE OF CONTENTS

PROTOCOL APPROVAL SIGNATURES 7

INVESTIGATOR'S AGREEMENT 8

TRIAL PERSONNEL 9

LIST OF ABBREVIATIONS 11

TRIAL SYNOPSIS 15

1. INTRODUCTION 21

1.1 Rationale for conducting trial 24
1.2 Risk-benefit evaluation 25
1.2.1 Potential benefits 25
1.2.2 Potential risks 25

2. TRIAL OBJECTIVES AND OUTCOMES 30

2.1 Objectives 30
2.1.1 Primary 30
2.1.2 Secondary 30
2.1.3 Exploratory 30
2.2 Endpoints 31
2.2.1 Primary 31
2.2.2 Secondary 31
2.2.3 Exploratory 31

3. TRIAL DESIGN 31

3.1 Overall trial design 31
3.2 Order of procedures, scheduling time windows and protocol deviations 37
3.3 Adaptive design 39
3.4 Rationale for trial design, doses and control groups 42
3.4.1 Justification for the selected dose level 42
3.4.2 Choice of participants for trial 43
3.4.3 Route and rate of administration 43
3.4.4 Precautions to be applied for dosing between participants in sentinel group and participants in the main group 43
3.4.5 Monitoring and communication of Adverse Events/Reactions 43
3.4.6 Investigator site facilities and personnel 44

4. DECISION-MAKING, RULES AND LIMITS 44
4.1 Definitions 44
4.2 Rules and limits governing decision-making 44
4.3 Safety Review Committee 45
4.3.1 SRC Meeting 45
4.4 Adverse Reaction (AR) rules 46

5. SELECTION AND WITHDRAWAL OF PARTICIPANTS 50
5.1 Number and Source of participants 50
5.2 Replacement participants 50
5.3 Inclusion Criteria 50
5.4 Exclusion Criteria 52
5.5 Participant restrictions 55
5.6 Criteria for withdrawal 58
5.6.1 Handling of withdrawals 58

6. TRIAL AND CONCOMITANT TREATMENTS 59
6.1 Investigational Medicinal Products (IMPs) 60
6.1.1 Packaging and labelling of IMPs 61
6.1.2 Drug administration 61
6.1.3 Storage of IMPs 63
6.1.4 Drug accountability 63
6.2 Treatment allocation and blinding 63
6.2.1 Participant randomisation 63
6.2.2 Methods for ensuring blinding 64
6.2.3 Methods for unblinding the trial 64
6.3 Concomitant medications/permitted medications 64
6.4 COVID-19 vaccinations 65

7. TRIAL METHODOLOGY 65
7.1 Medical history 65
7.2 Eligibility check 65
7.3 Prior and concomitant medication check 65
7.4 Beck depression inventory 65
7.5 Meals 65
7.6 Clinical laboratory assessments 66
7.6.1 Haematology, biochemistry and coagulation 67
7.6.2 Serology 67
7.6.3 Pregnancy testing 67

7.6.4 Urinalysis ... 67
7.6.5 Drugs of abuse ... 67
7.6.6 FSH assessment ... 68
7.7 Alcohol breath test ... 68
7.8 Vital Signs (blood pressure, pulse rate, respiratory rate and tympanic temperature) ... 68
7.9 Electrocardiographic (ECG) measurements ... 68
7.9.1 Recording of 12-lead ECGs ... 68
7.9.2 Safety review of 12-lead ECGs ... 69
7.9.3 24-hour Holter ECG ... 69
7.9.4 Real Time ECG Telemetry ... 69
7.9.5 Analysing and over-reading 12-lead ECG for the purpose of intensive cardiac assessments ... 69
7.10 Physical examination, height, weight and BMI ... 70
7.11 Pharmacokinetic assessments ... 70
7.11.1 PK blood samples ... 70
7.12 Volume of blood sampling ... 70
7.13 CYP (polymorphism) sampling ... 70

8. ADVERSE EVENTS ... 71

8.1 Definitions ... 71
8.2 Classification ... 73
8.2.1 Assessment of severity ... 73
8.2.2 Assessment of causality ... 74
8.2.3 Practical application of severity grading and causality assessment in relation to AR rules ... 76
8.2.4 Expectedness (reference safety information) ... 77
8.2.5 Adverse Events of Special Interest (AESI) ... 77
8.3 Recording of adverse events and follow-up ... 78
8.4 Reporting of Serious Adverse Events ... 78
8.5 Pregnancy ... 79
8.5.1 Pregnancy in female partners of male participants ... 80

9. QUALITY ASSURANCE AND QUALITY CONTROL ... 80

9.1 Quality Assurance and Quality Control ... 80
9.2 Monitoring ... 81

10. STATISTICAL ANALYSIS ... 81

10.1 Statistical analysis plan ... 81

10.2 Analysis sets 81
10.2.1 Safety set 81
10.2.2 PK set 81
10.2.3 ECG set 81
10.3 Statistical analysis of safety 82
10.4 ECG analysis 83
10.4.1 Baseline 83
10.4.2 Heart rate correction 83
10.4.3 Per timepoint analysis 84
10.4.4 Hysteresis 84
10.4.5 Concentration-QT_c analysis 84
10.4.6 Assay sensitivity 84
10.5 Pharmacokinetics 85
10.5.1 Evaluation of pharmacokinetic parameters 85
10.5.2 Statistical analysis on PK parameters 85
10.6 Handling of missing and incomplete data 86
10.7 Determination of sample size 86

11. DATA MANAGEMENT 86

11.1 Case Report Forms 87

12. SPONSOR'S AND INVESTIGATOR'S RESPONSIBILITIES 87

12.1 Sponsor's responsibilities 87
12.1.1 GCP compliance 87
12.1.2 Regulatory approval 88
12.1.3 Indemnity/liability and insurance 88
12.1.4 Protocol management 88
12.1.5 End of trial notification 88
12.1.6 Posting or submission of summary of clinical trial report to competent authorities of member states concerned and RECs 88
12.2 Investigator's responsibilities 89
12.2.1 GCP compliance 89
12.2.2 Protocol adherence and Investigator agreement 89
12.2.3 Documentation and retention of records 89
12.3 Ethical considerations 89
12.3.1 Informed consent 89
12.3.2 Research Ethics Committee (REC) approval 90
12.4 Confidentiality 90
12.5 Publication policy 91

13. REFERENCES 92

14. APPENDIX 1 95

LIST OF TABLES

Table 1: Planned treatment arms and IMP administration 18
Table 2: Class effect risk and risk mitigation features 27
Table 3: Clinical and non-clinical known risks and mitigation features 28
Table 4: Planned treatment arms and IMP administration 32
Table 5: Schedule of Assessments 34
Table 6 Schedule of Assessments (continued) 36
Table 7: Adaptive protocol features 39
Table 8: Minimum data requirements to proceed from sentinel dosing to dosing remaining participants 45
Table 9: Individual rules (decision 1) 47
Table 10: Cohort and trial rules (decisions 3 and 4) 48
Table 11: Participant restrictions 56
Table 12: IMP or placebo for each anticipated dose level 62
Table 13: Laboratory parameters 66
Table 14: Categorical grading of AE/ARs 74
Table 15: PK parameters 85

LIST OF FIGURES

Figure 1: Trial flow chart 19
Figure 2: Trial flow chart 33
Figure 3: Trial treatments and concomitant therapies 60

PROTOCOL APPROVAL SIGNATURES

Version 3.0, dated 07-FEB-2022

Sponsor's Approval

This protocol has been approved by Medicines for Malaria Venture (MMV).

Sponsor's Signatory:

Name: **Stephan Chalon MD PhD**

Signature: [redacted] **Date:** 08-Feb-2022

Name: **Andrea Kümmerle PhD**

Signature: [redacted] **Date:** DD-MMM-YYYY 08-Feb-2022

INVESTIGATOR'S AGREEMENT

I have read this MMV Protocol No. MMV_SMC_21_01:

A randomised, double-blind, placebo controlled, parallel group study in healthy adult volunteers to determine the tolerability and safety of pyronaridine (PYR) co-administered with piperaquine (PQP) under fasted conditions

I have fully discussed the objectives of this trial and the contents of this protocol with MMV representative(s).

I understand that the information in this protocol is confidential and should not be disclosed, other than to those directly involved in the execution or the ethical/regulatory review of the trial, without written authorisation from MMV. It is, however, permissible to provide information to a participant to obtain consent.

I agree to conduct this trial according to this protocol and to comply with its requirements, subject to ethical and safety considerations and guidelines, and to conduct the trial in accordance with ICH guidelines on GCP and with the applicable regulatory requirements.

I understand that MMV may decide to suspend or prematurely terminate the trial at any time for any reason as described in the MSA or Work Order with the clinical trial site; such a decision will be communicated to me in writing. Conversely, should I decide to withdraw from execution of the trial I will communicate my intention immediately in writing to MMV.

Principal Investigator:

Dr Ulrike Lorch MD FRCA FFPM

Signature: [redacted] **Date:** 08-Feb-2022

TRIAL PERSONNEL

Sponsor's Representative	Keith Berelowitz M.Sc.<br>Richmond Pharmacology Ltd.<br>[redacted]
Sponsor's Medical Director/Contact	Dr Stephan Chalon MD PhD<br>[redacted]
Sponsor's Clinical Lead/Contact	Dr Andrea Kümmerle PhD<br>[redacted]
Sponsor's Medical Monitor	Dr Annick Janin MD<br>Medical Monitor<br>AKJ Consulting<br>[redacted]
Sponsor site	Medicines for Malaria Venture (MMV)<br>[redacted]
Trial Site	Richmond Pharmacology Ltd.<br>[redacted]
Principal Investigator	Dr Ulrike Lorch MD FRCA FFPM<br>[redacted]
Co-Investigators	Dr Jörg Täubel MD FFPM FESC<br>Dr Andrew Stokes MB BCh BAO MSc
Clinical Laboratory	The Doctor's Laboratory<br>[redacted]
Bioanalytical Laboratory	Anita Kress<br>Swiss BioQuant AG

	[redacted]
PK Statistical Analysis and Reporting	Rachael White<br>PharmaKinetic<br>[redacted]
Data Management	Richmond Pharmacology Ltd.
Statistics	Richmond Pharmacology Ltd.
Qualified Person	Dilawaiz Afaqui
Pharmaco-vigilance	Oliver Abschütz<br>PrimeVigilance<br>[redacted]
Clinical Project Manager	Kapilkumar Undaviya M.Sc.<br>Richmond Pharmacology Ltd.
Medical Writing	Dr Andrew Stokes MB BCh BAO MSc<br>Dr Jasmin Walravens-Evans MB BS BSc

LIST OF ABBREVIATIONS

Abbreviation	Explanation
Ab	Antibody
ABPI	Association of the British Pharmaceutical Industry
ACT	Artemisinin-based Combination Therapies
AE	Adverse Event
AESI	Adverse Event of Special Interest
AIC	Akaike Information Criterion
ALP	Alkaline phosphatase
ALT	Alanine aminotransferase
AR	Adverse Reaction
AS	Artesunate
AST	Aspartate aminotransferase
$\%AUC_{extrap}$	Percentage of AUC that is due to extrapolation from l_{ast} to infinity
AUC	Area under the concentration-time curve
$AUC_{0-\infty}$	Area under the plasma concentration-time curve from time zero extrapolated to infinite time
AUC_{0-t}	Area under the plasma concentration curve from time zero up to the last quantifiable concentration
AV	Atrioventricular
β-hCG	Beta-human chorionic gonadotrophin
BW	Body weight
BMI	Body Mass Index
CI	Confidence interval
CL/F	Apparent total plasma clearance
C_{max}	Maximal plasma concentration
CRF	Case Report Form
CSP	Clinical Study Protocol
CV	Coefficient of Variation
CYP2C19	Cytochrome P2C19
CYP2C9	Cytochrome P2C9

Abbreviation	Explanation
CYP3A4	Cytochrome P3A4
CYP450	Cytochrome P450
D	Day
DBP	Diastolic blood pressure
DHA	Dihydroartemisinin
DHP	Data Handling Protocol
DHP STW	Data Handling Protocol for Scheduling Time Windows
DSUR	Development Safety Update Report
ECG	Electrocardiogram
EMA	European Medicines Agency
ET	Early Termination
fm	Fraction metabolised
FSH	Follicular Stimulating Hormone
GCP	Good Clinical Practice
GMP	Good Manufacturing Practice
h	Hours
Hb	Haemoglobin
HBc	Hepatitis B core
HBsAg	Hepatitis B surface Antigen
HCV	Hepatitis C Virus
hERG	the human Ether-à-go-go-Related Gene
HIV	Human Immunodeficiency Virus
HR	Heart rate
IB	Investigator’s Brochure
ICF	Informed Consent Form
ICH	International Conference on Harmonization
IMP	Investigational Medicinal Product
IPTp	Intermittent preventive treatment of malaria in pregnancy
IUD	Intrauterine device
IUS	Intrauterine hormone-releasing system

Abbreviation	Explanation
LLN	Lower Limit of Normal
LOQ	Limit of Quantification
MedDRA	Medical Dictionary for Regulatory Activities
MMV	Medicines for Malaria Venture
MTS	Master Treatment Schedule
MUSE®	MUSE® Cardiology Information System
N	Number of participants
n	Number of samples
NHS	National Health Service
n(LLOQ)	The number of samples <LLOQ
PBPK	Physiologically based pharmacokinetic
PI	Principal Investigator
PK	Pharmacokinetic
PQP	Piperaquine tetraphosphate
PR	Duration of the PR interval
PT	Preferred Term
PYR	Pyronaridine tetraphosphate
QRS	Duration of the QRS interval
QT	Duration of the QT interval
QT_c	Duration of the QT interval adjusted for heart rate
REC	Research Ethics Committee
RPL	Richmond Pharmacology Ltd.
RR	Respiratory rate
RSI	Reference Safety Information
SAE	Serious Adverse Event
SAP	Statistical Analysis Plan
SAR	Suspected Adverse Reaction
SAS®	Statistical Analysis System®
SBP	Systolic blood pressure
SD	Standard Deviation

Abbreviation	Explanation
SMC	Seasonal malaria chemoprevention
SP	Sulfadoxine-pyrimethamine
SmPC	Summary of Product Characteristics
SOC	System Organ Class
SOM	Study Operations Manual
SOP	Standard Operating Procedure
SPAQ	Sulfadoxine-pyrimethamine plus amodiaquine
SRC	Safety Review Committee
$t_{1/2}$	Terminal Elimination Half-life
TdP	Torsades de Pointes
TEAE	Treatment-Emergent Adverse Event
t_{max}	Time to reach maximum plasma concentration
ULN	Upper Limit of Normal
V_z/F	Apparent volume of distribution during the terminal phase
WHO	World Health Organization
WNCBP	Female participants of non-childbearing potential
WOCBP	Female participants of childbearing potential
λ_z	Terminal Rate Constant

For the purposes of this protocol, ‘Investigator’ refers to the Principal Investigator or their delegate.

TRIAL SYNOPSIS

**Protocol Reference:** MMV_SMC_21_01	**Trial drugs:** Pyronaridine and piperaquine	
**Title of the trial:** A randomised, double-blind, placebo controlled, parallel group study in healthy adult volunteers to determine the tolerability and safety of pyronaridine (PYR) co-administered with piperaquine (PQP) under fasted conditions.		
**Principal Investigator:** Dr Ulrike Lorch MD FRCA FFPM		
**Trial centre:**<br>Richmond Pharmacology Ltd.<br>[redacted]		
**Trial parts:** N/A	**Clinical phase:** 1 (the trial does not have therapeutic or prophylactic intent and does not plan to assess efficacy)	
**Objectives**	**Endpoints**	**Statistical analyses**
**Primary**		
• To determine the safety and tolerability of the registered dose for treatment of acute uncomplicated malaria of PYR (once daily for three days) and the registered dose for the treatment of acute uncomplicated malaria of PQP (once daily for three days) when administered alone and in combination, in comparison with placebo.	• The incidence, severity and relationship of Treatment-Emergent Adver e Event (TEAE).<br>• Proportion of participants with clinically significant changes in laboratory safety tests (haematology, biochemistry, coagulation and urinalysis).<br>• Proportion of participants with morphological and/or rhythm abnormalities on electrocardiogram (ECG).<br>• Proportion of participants with clinically significant changes in ECG time intervals (PR, QRS, QT and QT_c intervals) and clinically significant changes against baseline Holter.<br>• Proportion of participants with clinically significant changes in vital signs (systolic blood pressure, diastolic blood pressure and pulse rate).	• Adverse Events (AE), Adverse Events of Special Interest (AESI), Serious Adver e Event (SAE), vital signs, ECG parameters, and clinical laboratory data will be listed and summarised using descriptive statistics.<br>• All AEs will be summarised and listed by using System Organ Class (SOC) and Preferred Term (PT) assigned to the event using Medical Dictionary for Regulatory Activities (MedDRA). Furthermore, these events will be summarised by the maximum intensity. The number of participants who experienced drug-related AEs will also be summarised. Any SAEs, AESIs and/or AEs that led to withdrawal will be summarised and listed.

Objectives	Endpoints	Statistical analyses
**Secondary**		
• To determine the pharmacokinetics (PK) of PYR and PQP when administered alone and in combination for three days.	• PK parameters derived by non-compartmental methods including maximum observed plasma concentration (C_{max}), time to reach maximum plasma concentration (t_{max}), area under the plasma concentration-time curve from time zero to last detectable plasma concentration (AUC_{0-t}), area under the plasma concentration-time curve from time zero extrapolated to infinite ($AUC_{0-\infty}$), apparent total plasma clearance (CL/F), apparent volume of distribution during the terminal phase (V_z/F), terminal elimination rate constant (λ_z), and terminal elimination half-life ($t_{1/2}$).	• Non-compartmental analysis will be used for estimation of pharmacokinetic parameters.<br>• For individual plasma concentration data, the actual time of investigational medical product (IMP) administration and actual blood sampling time will be used in the derivation of the PK parameters. If there is any doubt in the actual time a sample was taken, then the scheduled time will be used.<br>• Area under the concentration-time curve (AUC) values will be calculated using the linear/log trapezoidal method, applying the linear trapezoidal rule up to C_{max} and the log trapezoidal rule for the remainder of the curve. Samples below Limit of Quantification (LOQ) prior to the first quantifiable concentration will be set to zero. Samples with concentrations below LOQ after the first quantifiable concentration will be set to 'missing' and omitted from the analysis. Other pharmacokinetic parameters will be calculated according to standard equations.
• To determine the relationship between PQP and ECG parameters (QT_c, QRS and PR), between PYR and ECG parameters, and to evaluate any impact of the combination of PYR and PQP on ECG parameters.	• Difference between baseline corrected QT_c following placebo and baseline corrected QT_c following PQP alone, and with PYR.	• Two complementary sets of analyses will be performed: a per timepoint analysis and a concentration-QT_c analysis.<br>• For all quantitative ECG parameters, descriptive statistics will be given for the change from baseline. In addition, for QT_c, a linear model will be fitted for each timepoint and the difference between each of the active treatments and placebo will

		be estimated based on this model and two-sided 90 % confidence intervals be given.<br><br>This analysis will be based on the change of QT_c from baseline and will use baseline QT_c and the concentrations of PYR and PQP as covariates. Treatment and time will be used as discrete fixed effects. A significant treatment effect (based on an F-test) will be considered an indication for model misfit. For the best fitting linear model, predictions of the effect on QT_c will be made at the mean concentrations of the moieties involved at the t_{max} for each of these moieties.
**Exploratory**		
**Objectives**	**Endpoints**	**Statistical analyses**
• To investigate the exposures to PQP metabolites (e.g. N-oxidated metabolite, provided a future bioanalytical method is available).	• PK parameters of PQP metabolites (e.g. N-oxidated metabolite) and metabolites to parent drug ratios.	• PQP metabolites PK parameters including metabolite to parent drug PK parameters will be calculated using non-compartmental methods from plasma concentration-time data and will be summarised by dose group using descriptive statistics.
• To determine if CYP genetic polymorphism influences drug PK and safety & tolerability.	• The relationship between CYP genetic polymorphism, PK parameters, and safety & tolerability.	• The relationship between CYP genetic polymorphism, PK parameters, and safety & tolerability will be summarised as applicable/appropriate.

Trial design

The trial is a single-centre, randomised, double-blind, placebo controlled, parallel group study to determine the safety, tolerability and PK of the registered dose for treatment of acute uncomplicated malaria of PYR + PQP, PYR + PQP placebo, PYR placebo + PQP, as well as PYR placebo + PQP placebo when administered orally once daily for three days to healthy adult male and female participants.

Forty (40) participants are planned to be enrolled and randomised to one of the four treatments in a 2:1:1:1 ratio, as described below:

- Treatment 1 (N=16): PYR + PQP

- Treatment 2 (N = 8): PYR + placebo for PQP
- Treatment 3 (N = 8): placebo for PYR + PQP
- Treatment 4 (N = 8): placebo for PYR + placebo for PQP.

All participants will receive a single dose of PYR or placebo, as well as a single dose of PQP or placebo, on the morning of Day 1, Day 2 and Day 3. Participants will fast for at least three hours prior to dosing and four hours after dosing. Within each treatment arm, fasted participants will take PYR/placebo tablets, followed by PQP/placebo tablets, at the following weight-dependent doses (Table 1).

Table 1: Planned treatment arms and IMP administration

Number of Participants	IMPs	Planned Dose (depending on Body Weight)	Number of Doses/ Dose Interval
16	PYR	540 mg (BW 50 kg - <65 kg); **OR**<br>720 mg (BW 65 kg or greater)	Single morning doses on D1, D2 and D3
	PQP	960 mg (BW 50 kg - <75 kg); **OR**<br>1280 mg (BW 75 kg or greater)	
8	PYR	540 mg (BW 50 kg - <65 kg); **OR**<br>720 mg (BW 65 kg or greater)	
	Placebo for PQP	N/A	
8	Placebo for PYR	N/A	
	PQP	960 mg (BW 50 kg - <75 kg); **OR**<br>1280 mg (BW 75 kg or greater)	
8	Placebo for PYR	N/A	
	Placebo for PQP	N/A	

Abbreviations: BW: body weight; D: day; kg: kilograms; mg: milligrams; PQP: piperaquine; PYR: pyronaridine.

Participant screening will take place between Day -22 and Day -2 (Figure 1). Each participant will receive verbal and written information, then sign the Informed Consent Form (ICF), before any screening procedures take place. Participants will be admitted on Day -1 and dosed once in the mornings of Day 1, Day 2 and Day 3. Participants in the sentinel group will be discharged on Day 7 and will return to the unit for outpatient assessments on Days 8, 15 and 22. If safety and tolerability data allow, and if agreed by the Safety Review Committee (SRC), the remaining (non-sentinel) participants will be discharged on Day 5 and will return to the unit for outpatient assessments on Days 6, 8, 15, 22. Final assessments will be carried out at the follow-up visit on Day 30 (+/-1 day). The trial Schedule of Assessments (Table 5 and Table 6) details all assessments that will take place during the trial. The trial design features as well as the number of participants may be adapted according to the Adaptive Features (Table 7). This trial will use a sentinel dosing strategy. For full details see Section 3.4.4.

Figure 1: Trial flow chart

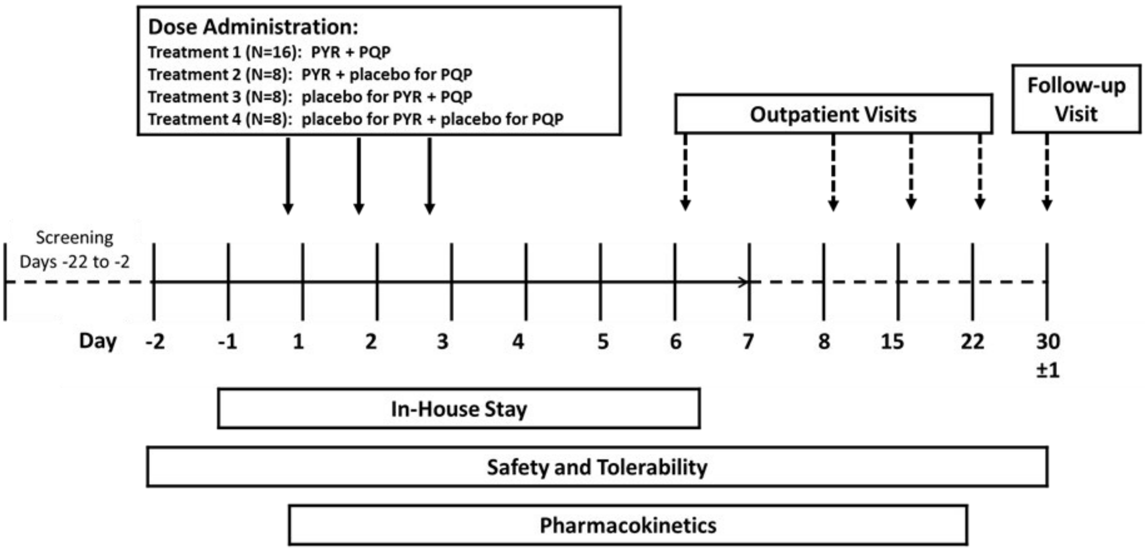

Abbreviations: N: number of participants; PQP: Piperaquine; PYR: Pyronaridine.
Participants in the sentinel group will remain in-house until Day 7. Participants in the main group may be discharged on Day 5, based on the available safety and tolerability data from the sentinel group and as decided by the Safety Review Committee (SRC). The Day 6 OP visit will apply to the non-sentinel group only.

Number of participants

40 healthy male or female participants will be recruited onto the trial. Sufficient reserve participants will be identified to ensure that 40 participants complete the trial. For an individual participant: A participant is considered to have reached the end of the study if the participant has completed the last visit (Day 30 for this protocol). In each cohort, if more than two discontinuations due to non-safety related reasons occur, additional participants may be recruited to replace the discontinued participants. This would require prior agreement with the trial Sponsor.

Main criteria for admission

Healthy black male or female participants, of Sub-Saharan African origin (defined as participants whose parents are both black and are of Sub-Saharan African origin) will be included if they are aged between 18 and 45 years (inclusive), weigh 50 kg or greater and have a Body Mass Index (BMI) between 18 kg/m^2 and 28 kg/m^2 (inclusive). Participants of childbearing potential must use highly effective methods of birth control. All efforts will be made to ensure a balanced male: female ratio throughout the trial. Every effort will be made to recruit participants in order to stratify randomisation according to weight, so that a minimum of two participants receive each of the seven treatment options (see Table 12 for details).

Main exclusion criteria are: Any female participant detected to be pregnant, breastfeeding, or who is likely to become pregnant during the trial or any male participant with a female partner(s) who is (are) pregnant or lactating from the time of the administration of trial medication. Current or recurrent disease (or condition) which, in the opinion of the Investigator, may either put the participant at risk because of participation in the trial, may influence the result of the trial, or the participant’s ability to participate in the trial.

Anticipated test treatment(s) and mode of administration

Oral recommended malaria treatment doses of PYR and PQP administered once daily on Day 1, Day 2 and Day 3, in the fasted state. See Section 6.1.2 for details.

Reference treatment(s) and mode of administration

Oral matched placebo tablets for PYR and PQP.

1. INTRODUCTION

In 2019, an estimated 229 million cases of malaria occurred worldwide, with approximately 94 % occurring in WHO African Region, leading to 409 000 deaths, of which about two thirds were in children under five years of age [1]. In addition, there were an estimated 33 million pregnancies in the moderate to high transmission countries of the WHO African Region, of which 35 % (12 million) were exposed to malaria infection during pregnancy [1].

Case management with highly effective antimalarial drugs has contributed to the decrease in malaria morbidity and mortality. However, preventive administration of antimalarial drugs is also recommended for use in selected high-risk populations, irrespective of malaria infection status, both to treat any unrecognized Plasmodium infections and to prevent new ones. These preventive treatments prevent or cure undetected malarial illness with the goal to achieve therapeutic drug levels in the blood throughout the period of greatest risk [1]. WHO strongly recommends with a high-certainty evidence the following malaria chemopreventive therapies: seasonal malaria chemoprevention (SMC), intermittent preventive treatment in infants (IPTi), and intermittent preventive treatment of malaria in pregnancy (IPTp) [1,2].

Sulfadoxine-pyrimethamine (SP) is recommended for IPTp along with the antenatal care visits starting from the second trimester of pregnancy and IPTi along with infant vaccination taking place usually at 10 weeks, 14 weeks and 9 months of age. Sulfadoxine-pyrimethamine plus amodiaquine (SPAQ) is recommended for SMC as a monthly administration during the malaria season for three to four consecutive months to children between three months and five years of age in areas of highly seasonal malaria transmission [2]. In this context, SPAQ demonstrated a protective efficacy of >80 % in Burkina Faso [3].

Despite the strong public health evidence of chemoprevention, only SP and SPAQ are currently recommended by WHO for chemoprevention, which use is currently threatened by increasing resistance to SP [4,5]. MMV's strategy is to re-combine existing and approved antimalarials used individually for the treatment of malaria, for the purpose of malaria chemoprevention. As part of this strategy, a review of the potential efficacy and duration of protection, the safety and tolerability, as well as the risk of emergence of resistance of currently approved products has been conducted, with a view to identify a new combination of two registered products that may be suitable for malaria chemoprevention.

A combination of pyronaridine (PYR) and piperaquine (PQP) was considered to have potential for this purpose. In the European Union and in many African countries, PQP is marketed in a fixed dose combination with the short acting antimalarial dihydroartemisinin (DHA) as Eurartesim® for treatment of acute uncomplicated malaria. PYR in a fixed dose combination with artesunate (AS) received positive scientific opinion from EMA through Article 58 and is registered in many malaria endemic countries in Africa and South-East Asia as Pyramax® for treatment of acute uncomplicated malaria for both adults and children. The efficacy of both PYR and PQP combined to artemisinin derivatives have been demonstrated in the treatment of uncomplicated malaria in various clinical trials in different regions and these combinations have been in clinical use for at least 20 and 40 years, respectively [4,6].

Based on efficacy data provided to support registration for malaria treatment, PYR and PQP are long-acting antimalarials, which extended half-lives shall confer a

protection for one month with the standard three-day regimen. Additionally, DHA-PQP was associated with a protective effect of over 75 % in children three to 59 months old [3]. It is therefore assumed that a monthly based treatment of the three-day regimen of both PQP and PYR (for three to four consecutive months during the malaria season) shall achieve adequate efficacy for malaria prophylaxis.

Since malaria chemoprevention is administered to otherwise healthy infants, children, and pregnant women (who are either parasite-free or have circulating parasites but are asymptomatic), treatments must show a good safety profile and be well tolerated. In an internal thorough MMV evaluation of multiple candidates, the safety and tolerability profile of these two compounds did not show any prohibitive overlap [7,8]. In addition, until today, neither PYR nor PQP showed any teratogenic liabilities and they are therefore expected to be a potential safe option for malaria prevention in pregnant women and women of child-bearing potential (WOCBP) which is a key asset towards malaria eradication.

Finally, the combination of these two compounds with distinct mechanisms of action is expected to be at low risk of rapid emergence of resistance [4,6].

Mechanism of action, clearance and pharmacokinetics

PYR inhibits the formation of β-haematin and thus prevents the malarial parasite from neutralizing haem, which is toxic to the parasite. Additionally, by forming a drug-haematin complex pyronaridine inhibits glutathione-dependent degradation of haematin and enhances haematin-induced lysis of red blood cells. Both of these actions lead to parasite death [6]. In healthy participants and patients with malaria, peak plasma concentrations of PYR are generally reached between two hours and eight hours post-dose. Pyronaridine appears to have many potential metabolites, with several metabolic pathways involved (including CYP3A4 and CYP2D6). Pyronaridine elimination half-life in adults is between 14 days and 18 days.

PQP's mechanism of action is unknown, but it likely mirrors that of chloroquine, a close structural analogue. Chloroquine binds to toxic haem (derived from the patient's haemoglobin) within the malaria parasite, preventing its detoxification via a polymerisation step [4]. In humans, PQP has a t_{max} of approximately five hours. PQP is mainly metabolized by CYP3A4 and to a lesser extent by CYP2C9 and CYP2C19. PQP is a CYP3A4 inhibitor, and a clinical study of Eurartesim® with midazolam showed a modest increase in the exposure of midazolam (<two-fold) [4]. The terminal elimination half-life of PQP is around 22 days for adult patients.

Safety and tolerability

Through their extensive use in many African and South-East Asian countries, the safety and tolerability of PYR and PQP for the treatment of uncomplicated malaria when combined to AS and DHA respectively, are well established. Safety data from a European registry have also been recently published for DHA-PQP [9].

The main safety signal observed with PYR in healthy participants and malaria patients consists of transient asymptomatic AST/ALT elevations which are not associated with bilirubin changes. The most frequently reported AEs (>1/100 to <1/10) are headache, eosinophilia, neutropenia, anaemia, increased platelet count, vomiting (2.2 % to 2.5 %), abdominal pain, bradycardia, transaminase

increases and hypoglycaemia. Additionally, analyses performed in malaria patients suggest that no clinically concerning QT_c prolongation is expected at therapeutic concentrations of PYR [10].

PQP as a combination antimalarial with DHA is well tolerated in both adults and children, with the main AEs reported being gastrointestinal disturbances such as nausea/vomiting, abdominal pain and diarrhoea [11,12]. Although transient asymptomatic ALT/AST elevations have also been described including in Phase 1 participants, these are generally mild in severity and considered as uncommon. PQP is known to induce QT_c prolongation in human participants [13,14]. Despite significant hERG blockade with PQP and a mean prolongation of 15-20 msec (fasting state) demonstrated in multiple Phase 1 studies, a meta-analysis has recently concluded that patients treated with the registered three-day regimen of DHA-PQP are not at higher risk of torsades de pointes (TdP), ventricular tachycardia or other arrhythmias when compared with the normal population [15]. Based on these observations, although inducing a QT_c prolongation that exceeds the ICH/FDA regulatory threshold of 10 msec, PQP is currently not considered as a torsadogenic drug provided it is taken in a fasted state and other clinical risk factors for QT_c prolongation are appropriately controlled [16].

Based on the reported clinical safety profile of the two drugs, the three major toxicities of focus with combination over three days will be:

1) gastrointestinal toxicity (reported with both drugs)
2) liver toxicity (primarily expected with PYR) and
3) QT_c prolongation (primarily expected with PQP).

Potential for interaction between PQP and PYR

The potential for pharmacokinetic interaction between PQP and PYR, leading to increased exposure and subsequent risk of toxicities has been assessed as follows. Based on in vitro data, PQP is a CYP3A4 time-dependent inhibitor, and pyronaridine a CYP3A4 substrate (estimated fm = 0.6). A clinical study evaluating the PK of PYR in the presence of ritonavir, a typical CYP3A4 time-dependent inhibitor, showed no drug-drug interaction [17]. Therefore, the risk of possible drug-drug interaction between PYR and CYP3A4 inhibitors is considered to be low.

The potential CYP3A4-mediated drug-drug interaction between PQP (as a perpetrator) and PYR (as a substrate) was simulated using Simcyp PBPK modelling, which confirmed its low likelihood (less than 25 % increase in AUC) [18]. Consequently and since the two drugs will be administered in a fasting state, if an increase in PQP exposure is observed in the presence of PYR, the potential impact on the PQP-induced QT_c prolongation should be less than the one documented in the presence of a high fat meal. There is however a risk of accumulation in the liver with PYR as seen in preclinical dog studies where more than 10 % of the dose remained in the liver eight months after a single oral administration [19].

In addition to the risk of accumulation there is risk of potentiation and summation of the overlapping gastrointestinal, CNS (dizziness), liver and cardiac toxicity seen with PQP and PYR. The following aspects of the pharmacokinetic and Adverse Event profiles of PQP and PYR were considered to determine the inclusion and exclusion criteria and to design the tolerability and safety monitoring of this trial:

- the overlapping Adverse Events profiles of PQP and PYR in healthy participants and/or malaria patients (e.g. nausea, vomiting, diarrhoea, dizziness and increases of liver AST and ALT) indicate a potential for additive Adverse Events
- PQP is known to prolong QT_c in humans and this effect is concentration-related [7]
- Based on a study with reduced number of digitalized ECGs, there is no evidence of clinically significant QT_c prolongation with PYR in malaria patients [18], however one cannot formally exclude that PYR may influence the expected QT_c prolongation with PQP in healthy participants with a maximal effect expected on the last day of administration
- PQP has a Terminal Elimination Half-life of approximately 22 days. However, approximately 1 % of the C_{max} is predicted on 672 hours (i.e. 28 days) after the administration of 1280 mg in healthy adults [22]. Therefore, a follow-up of 30 days (i.e. 28 days after last dose) is considered acceptable to ensure that possible delayed effects on safety laboratory tests are appropriately documented.

Close clinical monitoring will include LFT measurements (including AST, ALT, bilirubin and alkaline phosphatase [ALP]) and ECG measurements in triplicate. Participants will remain in the unit under medical supervision until at least 48 hours following the final dose (i.e. at least 120 hours [h] inpatient) and will be followed up to Day 30 (+/-1 day).

1.1 Rationale for conducting trial

This study in healthy adults will be the first one to evaluate combined administration of PYR and PQP in humans, with a view for potential use in the future as seasonal malarial chemoprevention for populations at risk based in endemic areas. A parallel group design (n=40) including a placebo cohort will be used to evaluate safety/tolerability and PK of the two investigational medicines either as monotherapy or in combination. To our knowledge, this study will be the first human study with this new combination. The approved adult malaria treatment doses of PYR and PQP have been chosen for this trial.

The trial will involve four treatment arms:

- Treatment 1 (N=16): PYR + PQP
- Treatment 2 (N=8): PYR + placebo for PQP
- Treatment 3 (N=8): placebo for PYR + PQP
- Treatment 4 (N=8): placebo for PYR + placebo for PQP.

This will facilitate discrimination of the contribution of the investigational medicines to any safety/tolerability signal and determine the pharmacokinetic of PYR and PQP when administered alone and in combination.

Given the ultimate target population, the study will be carried out in healthy black participants of sub-Saharan African origin (defined as participants whose parents are both black and are of Sub-Saharan African origin).

1.2 Risk-benefit evaluation

1.2.1 Potential benefits

Healthy participants will take PYR and PQP for research purposes only. It is not anticipated that participants will receive any medical benefit apart from a general health examination.

1.2.2 Potential risks

1.2.2.1 Piperaquine (PQP)

The risks of PQP are described in detail above in the Safety and Tolerability section.

Further SOC specific risks and mitigation strategies are described below in the Risk Management table.

The risk of PQP-induced QT_c prolongation will be reduced appropriately by controlling for other clinical risk factors for QT_c prolongation [20]. As precautionary measures pertaining to QT_c effects of PQP, the European Medicines Agency (EMA) has advised that:

- DHA-PQP (Eurartesim®) not be administered with a meal to avoid food-induced increase in PQP C_{max} and consequently possible clinically relevant QT_c prolongation
- baseline and post-dose ECGs be obtained
- recent exposure to concomitant drugs known to induce QT_c prolongation be avoided [4,14].

Therefore, during this trial, healthy participants with risk factors for QT_c prolongation will be excluded and enrolled participants will receive PQP under fasting conditions and will be monitored for QT_c effects.

1.2.2.2 Pyronaridine (PYR)

Transient ALT/AST elevations above 3 x ULN are commonly reported in clinical trials investigating the three-day regimen of PYR-AS (pyronaridine-artesunate, Pyramax®). These elevations have been reported in both patients treated for acute uncomplicated malaria and healthy participants enrolled in clinical pharmacology studies. The overall pattern seems consistent across studies with a transient asymptomatic elevation in ALT/AST without bilirubin elevation which for most of the episodes occurred after the three-day treatment, within three and seven days after the initial dose and resolved within 21 days after reaching a peak value between eight and fifteen days after the first day of treatment. Higher elevations of >10 x ULN in patients and >5 x ULN in healthy have also been reported but are less common.

Concomitant elevation in bilirubin and possible Hy's Law cases have not been reported in healthy participants. In a retrospective pooled analysis of completed trials in patients, a total of 16 cases defined as concomitant elevations in ALT or AST >3 x ULN and total bilirubin >2xULN (i.e. Hy's Law cases) were reported in the clinical development program for PYR-AS. Ten cases were reported in malaria patients receiving PYR-AS and six were also reported in patients receiving one of the comparator malaria drugs which could possibly suggest a confounding background effect of the disease. Of these 16 cases, the Independent Safety

Monitoring Board adjudicated six possible Hy's Law cases in the PYR-AS group and four in the comparator drugs group.

A total of four Phase 1 studies were conducted with PYR-AS in healthy participants. A SAD-MAD-Food Effect study was conducted in healthy South Korean participants [21] and three subsequent clinical pharmacology studies including a human C14 study were performed in Switzerland [22,23,24]. With the exception of the human ADME study where PYR was administered as a single oral dose, all these Phase1 studies have evaluated the three-day therapeutic regimen of PYR-AS for acute uncomplicated malaria. Of note, the safety laboratory tests monitoring in the Asian study was limited to two post-dose time-points (72h and Day 11) while the two other studies (both DDI studies) used a very intensive monitoring with almost daily for one week and then weekly ALT/AST/bilirubin evaluations up to study end which covered a full month of observation. In the two DDI studies with ritonavir and metoprolol the LFT monitoring obtained for the control arm (i.e. PYR-AS alone) showed ALT/AST elevations with normal bilirubin consistent with the pattern described in malaria patients [22,23]. For example, in the Metoprolol DDI study, the control group of Caucasian participants dosed with PYR-AS alone over three days in Period 1 showed the following incidence of asymptomatic ALT elevations: 37 % (1-3 x ULN), 3 % (3-5 x ULN) and 10 % (>5 x ULN, including one case >20 x ULN). Similar incidences were observed for AST with 3 % of the study participants reaching values in the range 10-20 x ULN [23]. In the MAD study conducted in South Korea with four incremental doses of PYR-AS, only one case of ALT/AST elevations (>2 x ULN) was reported. However, these results should be interpreted with caution due to the very limited number of timepoints for safety laboratory tests: the study schedule did not include ALT/AST/bilirubin evaluation between Day 4 and Day 11. Additionally, a statistical comparison conducted on ALT/AST changes from baseline to Day 4 or to Day 11 in this Phase 1 study showed a statistically significant increase in ALT/AST suggesting a trend for elevation versus placebo particularly at the two highest doses of PYR 12 and 15 mg/kg [21].

Other most frequently reported AEs (>1/100 to <1/10) are headache, eosinophilia, neutropenia, anaemia, increased platelet count, vomiting (2.2 to 2.5 %), abdominal pain, bradycardia, transaminase increases and hypoglycaemia [18]. Additionally, analyses performed in malaria patients suggest that no clinically concerning QT_c prolongation is expected at therapeutic concentrations of PYR [10].

1.2.2.3 Risk management

This trial will be conducted at an accredited Phase 1 clinical trial unit by an experienced Investigator and well-trained medical staff and technical staff with ample experience in the conduct of early phase clinical trials. The trial has been designed to safely include suitable participants, monitor, treat and communicate potential expected Adverse Reactions as well as potential unexpected Adverse Events. Additionally, a sentinel dosing strategy will be used; please refer to protocol Section 3.4.4.

Although not expected, participants will be closely monitored for potential Serious Adverse Events. There are also special AR rules (both individual and group) that apply to the most significant risks (see Section 4.4).

In applying the above risk management strategies, the overall risk to participants in this trial is considered low.

There is currently no clinical experience with PYR and PQP given in combination. Potential risks will be closely monitored for as part of the safety evaluations being performed in this trial. The risk mitigation measures are summarised in Table 2 and Table 3 below.

Table 2: Class effect risk and risk mitigation features

Area	Other factors	Risk Mitigation
Class Effect	Falls in haemoglobin are common after effective treatment with a variety of anti-malarial drugs and have been reported in patients with Pyramax® (PYR-artesunate) and Eurartesim® (PQP-DHA) [4,19].	1) Standard monitoring as with all early phase studies.<br>2) Close monitoring of full blood count (including haemoglobin and white blood cell/ neutrophil count) will be conducted at regular intervals throughout the trial.

Table 3: Clinical and non-clinical known risks and mitigation features

Target System	Effect	Risk Mitigation
Renal System	PQP: hepatorenal function and urinalysis did not change in study of PQP administration (500mg single dose in healthy adults) [25]. No trials have been conducted with Eurartesim® or PQP alone in patients with renal impairment.<br><br>PYR: Contraindicated in Severe renal failure.<br>Blood, protein or white cells in the urine have been seen uncommonly with Pyramax®.<br>Pre-clinical study in rats:<br>Pyronaridine decreased urine volume with a concomitant increase in density and electrolyte concentration at 500 mg/kg in the rat (no effect at 33 and 100 mg/kg). See Table 4-1 in PYR IB.	1) Standard monitoring as with all early phase studies.<br>2) Urinalysis and safety bloods including urea, creatinine and glomerular filtration rate will be taken at regular intervals highlighted in the Schedule of Assessments (Table 5 and Table 6).<br>3) Additional assessments may be completed if required (including renal ultrasound, unscheduled microscopy or 24-hour urine collection).
Hepatic System	PQP: Overall considered as not commonly associated with LFT derangements [25].<br>LFT elevations have been seen uncommonly in healthy participants and malaria patients.<br><br>PYR: is associated with transient asymptomatic increases in liver enzymes without bilirubin elevation in both healthy participant and patient [8].<br><br>Please see Risk Section 1.2.2.2 above for further details.	1) Specific Inclusion and Exclusion Criteria will be used to select potential participants (Sections 5.3 and 5.4).<br>2) Extensive liver function testing will be completed including ALP, AST, ALT, bilirubin, GGT, Total protein as well as Albumin and Globulin, and Coagulation will be measured as per the clinical Schedule of Assessments (Table 5).<br>3) Previous exposure to Hepatitis B (+/- D) and C will be assessed (excluding participants who have previous exposure).<br>4) Specific AR rules will be implemented.<br>5) Additional assessments could be added depending on clinical requirements, including a FibroScan of the liver.
Cardiovascular system	PQP: alone known to induce QT_c prolongation in human participants with a well-documented exacerbation when the drug is taken with food [12,13]; also associated with tachycardia.<br><br>Please see Risk Management text above for further details.<br><br>PYR: not known to induce clinically concerning QT_c prolongation in human participants but no formal concentration/QT_c modelling in healthy participants is available.	1) Comprehensive monitoring and intensive cardiac assessments (including screening Holter and Telemetry D1-D3 with intensive time matched 12-Lead ECGs on D1 and D3).<br>2) Three hour fasted state dosing to reduce C_{max} of PQP and QT_cF risk. Four hour post dose fast also.<br>3) Exclusion of participants on QT_cF prolonging medications (Section 5.4)<br>4) Strict Exclusion Criteria surrounding history of

Target System	Effect	Risk Mitigation
	Phase I CYP3A4 DDI study of Pyronaridine-DHA with ritonavir, there was no relationship between QTcF change and plasma concentrations of pyronaridine or DHA [23].<br>PYR: more commonly associated with bradycardia in patients. Decrease in heart rate correspond to reduction in fever of malarial infection.<br>PYR use associated with palpitations and ventricular extrasystoles albeit uncommon (≥1/1000 to <1/100).	predisposing cardiac conditions (Section 5.4).
Respiratory System	PQP: No effects noted in human studies.<br><br>PYR:<br>Pre-Clinical Study:<br>A transient increase in respiratory rate was observed at a dose of 500 mg/kg two hours post- dose in a pre-clinical study in guinea pigs. No effects noted at doses of 33 and 100 mg/kg or at any other timepoint [19].<br>Human Malarial Studies:<br>Bronchitis, rhinitis and cough have uncommonly been seen in PYR-AS combination treatment of uncomplicated malaria.	1) Standard monitoring as with all early phase studies.
Central nervous system	PQP: headache (common), dizziness (uncommon) and convulsion (rare/very rare) has been seen in DHA-PQP combination clinical trials.<br>Asthenia commonly seen in DHA-PQP combination.<br><br>PYR: Headache commonly seen with PYR use. Paraesthesia and insomnia less commonly seen. Insomnia or fatigue/asthenia has been seen in PYR-AS combination clinical trials.	1) Standard monitoring as with all early phase studies.<br>2) Warning regarding driving or operating machinery upon discharge if dizziness or fatigue/asthenia occurs.
Haematological system	PQP: Haematological indices did not change as a result of PQP administration [25].<br>Anaemia and a reduction of haemoglobin commonly seen with DHA-PQP use. [25]<br><br>PYR: eosinophilia, neutropenia, anaemia, reduced haemoglobin and increased platelet count [19].	1) Specific inclusion and exclusion criteria will be used to select potential participants.<br>2) Close monitoring of full blood count (including haemoglobin, white blood cell/neutrophil and platelet count) will be conducted at regular intervals throughout the trial.<br>3) Specific AR rules related to haematology.

Target System	Effect	Risk Mitigation
Gastrointestinal system	PQP: gastrointestinal disturbances such as nausea/vomiting, abdominal pain and diarrhoea [9,10].	1) Standard monitoring as with all early phase studies.<br>2) Regular biochemistry blood samples
	PYR: nausea/vomiting (2.2 % to 2.5 %), abdominal pain, diarrhoea and constipation [8].	
Reproductive system	PQP: no specific data relating to the effects of PQP on human fertility; however, to date no AEs have been reported during clinical use [25].<br>Animal studies: In reproductive studies, PQP did not induce malformation in rats or rabbits [25].	1) Specific contraceptive requirements will be in place for participants.<br>2) Contraceptive guidance will be used in Inclusion Criteria (Section 5.3).<br>3) Female participants will need evidence of negative pregnancy tests prior to dosing.
	PYR: no adequate data on the use of pyronaridine alone in human pregnancy or lactation.<br>Animal studies: pyronaridine did not show any teratogenic effects nor effects on fertility or reproductive performance were observed at doses three-fold higher than the proposed human exposure [19].	

2. TRIAL OBJECTIVES AND OUTCOMES

2.1 Objectives

2.1.1 Primary

- To determine the safety and tolerability of the registered dose for treatment of acute uncomplicated malaria of PYR (once daily for three days) and the registered dose for the treatment of acute uncomplicated malaria of PQP (once daily for three days) when administered alone and in combination, in comparison with placebo.

2.1.2 Secondary

- To determine the pharmacokinetics (PK) of PYR and PQP when administered alone, and in combination, for three days.
- To determine the relationship between PQP and ECG parameters (QT_c, QRS and PR), between PYR and ECG parameters, and to evaluate any impact of the combination of PYR and PQP on ECG parameters.

2.1.3 Exploratory

- To investigate the exposures to PQP metabolites (e.g. N-oxidated metabolite, provided a future bioanalytical method is available).

- To determine if CYP genetic polymorphism influences drug PK and safety & tolerability.

2.2 Endpoints

2.2.1 Primary

- The incidence, severity and relationship of Treatment-Emergent Adverse Events (TEAEs).
- Proportion of participants with clinically significant changes in laboratory safety tests (haematology, biochemistry, coagulation and urinalysis).
- Proportion of participants with morphological and/or rhythm abnormalities on electrocardiogram (ECG).
- Proportion of participants with clinically significant changes in ECG time intervals (PR, QRS, QT and QT_c intervals) and clinically significant changes against baseline Holter.
- Proportion of participants with clinically significant changes in vital signs (systolic blood pressure, diastolic blood pressure and pulse rate).

2.2.2 Secondary

- PK parameters derived by non-compartmental methods including maximum observed plasma concentration (C_{max}), time to reach maximum plasma concentration (t_{max}), area under the plasma concentration-time curve from time zero to last detectable plasma concentration (AUC_{0-t}), area under the plasma concentration-time curve from time zero extrapolated to infinite ($AUC_{0-\infty}$), apparent total plasma clearance (CL/F), apparent volume of distribution during the terminal phase (V_z/F), terminal elimination rate constant (λ_z), and terminal elimination half-life ($t_{1/2}$).

  Difference between baseline corrected QT_c following placebo and baseline corrected QT_c following PQP alone, and with PYR.

2.2.3 Exploratory

- PK parameters of PQP metabolites (e.g. N-oxidated metabolite) and metabolites to parent drug ratios.
- The relationship between CYP genetic polymorphism, PK parameters, and safety & tolerability.

3. TRIAL DESIGN

3.1 Overall trial design

This will be a single-centre, randomised, double-blind, placebo-controlled, parallel group study to determine the safety, tolerability and PK of the registered dose to treat acute uncomplicated malaria of PYR + PQP, PYR + PQP placebo, PYR placebo

+ PQP, as well as PYR placebo + PQP placebo when administered orally once daily for three days to healthy adult male and female participants.

Forty (40) participants are planned to be enrolled and randomised to one of the four treatments in a 2:1:1:1 ratio as described below:

- Treatment 1 (N=16): PYR + PQP
- Treatment 2 (N=8): PYR + placebo for PQP
- Treatment 3 (N=8): placebo for PYR + PQP
- Treatment 4 (N=8): placebo for PYR + placebo for PQP.

All participants will receive a single dose of PYR or placebo, as well as a single dose of PQP or placebo, on the morning of Day 1, Day 2 and Day 3. Participants will fast for at least three hours prior to dosing and four hours after dosing. Within each treatment arm, fasted participants will take PYR/placebo tablets, followed by PQP/placebo tablets, at the following weight-dependent doses (Table 4).

Table 4: Planned treatment arms and IMP administration

Number of Participants	IMPs	Planned Dose (which depends on Body Weight [BW])	Number of Doses/ Dose Interval
16	PYR	540 mg (BW 50 kg - <65 kg); **OR**<br>720 mg (BW 65 kg or greater)	Single morning doses (fasted state) on D1, D2 and D3
	PQP	960 mg (BW 50 kg - <75 kg); **OR**<br>1280 mg (BW 75 kg or greater)	
8	PYR	540 mg (BW 50 kg - <65 kg); **OR**<br>720 mg (BW 65 kg or greater)	
	Placebo for PQP	N/A	
8	Placebo for PYR	N/A	
	PQP	960 mg (BW 50 kg - <75 kg); **OR**<br>1280 mg (BW 75 kg or greater)	
8	Placebo for PYR	N/A	
	Placebo for PQP	N/A	

Abbreviations: BW: body weight; D: day; IMP: investigational medicinal product; kg: kilograms; mg: milligrams; PQP: piperaquine; PYR: pyronaridine.

Participant screening will take place between Day -22 and Day -2. Each participant will receive verbal and written information, then sign the Informed Consent Form (ICF), before any screening procedures take place. Participants will be admitted on Day -1 and dosed once in the mornings of Day 1, Day 2 and Day 3. Participants in the sentinel group will be discharged on Day 7 and will return to the unit for outpatient assessments on Days 8, 15, 22. Non-sentinel group participants may be discharged on Day 5 and return for outpatient assessments on Days 6, 8, 15

and 22, provided there are no clinically significant safety and tolerability signals and the SRC are in agreement.

Final assessments will be carried out at the follow-up visit on Day 30 (+/-1 day). The trial Schedule of Assessments (Table 5 and Table 6) details all assessments that will take place during the trial. The study design features as well as the number of participants may be adapted according to the Adaptive Features (Table 7). This trial will use a sentinel dosing strategy. For full details see Section 3.4.4.

Figure 2: Trial flow chart

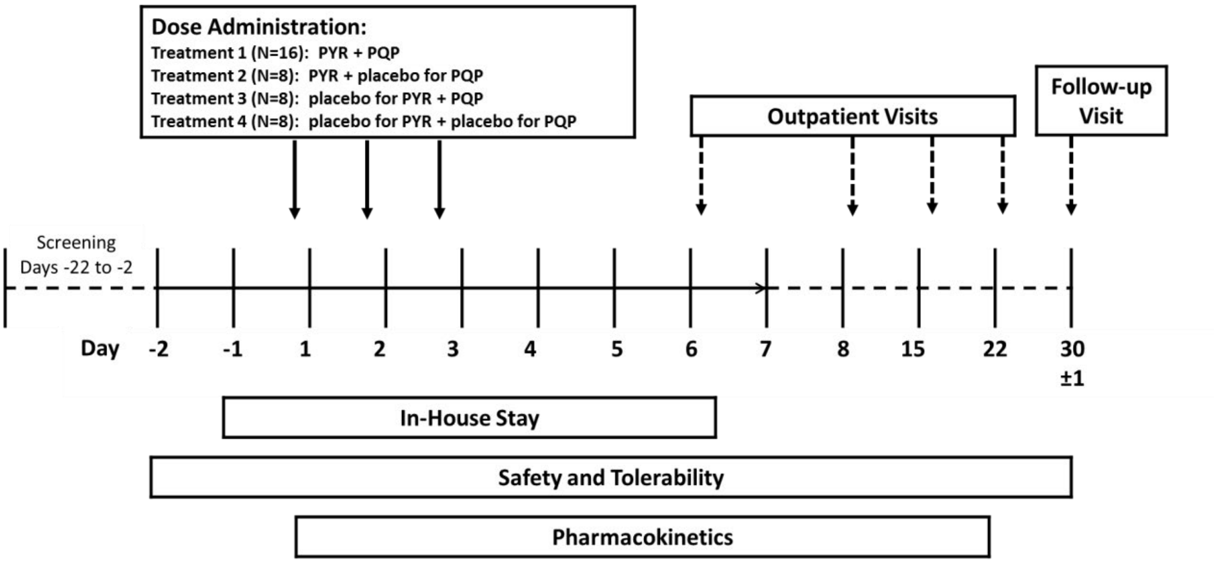

Abbreviations: N: number of participants in corresponding treatment arm; PQP: Piperaquine; PYR: Pyronaridine.
As long as the data from the sentinel group demonstrate acceptable safety and tolerability, the participants in the non-sentinel group will be discharged on Day 5, but this decision will be made by the Safety Review Committee (SRC) (See Table 7). The Day 6 OP visit will apply to the non-sentinel group only.

Table 5: Schedule of Assessments

Procedure	Screening (D-22 to D-2)	D-1	D1	D2	D3	D4	D5	D6	D7[11] Sentinel Group only	D8	D15	D22	Last visit D30 (±1)
In-house stay		X	X	X	X	X	X[11]	X	X				
Outpatient visit								X		X	X	X	X
Informed consent	X												
Eligibility check	X	X											
Medical history/ demographics	X	X[7]											
Prior & concomitant medications	X	X	X	X	X	X	X	X	X	X	X	X	X
Smoking history and current	X												
Beck depression inventory questionnaire	X												
Urine drugs of abuse screen	X	X											
Alcohol breath test	X	X											
Serology HIV 1&2, Hepatitis B&C	X												
Serum pregnancy test (females)	X	X											X
FSH (post-menopausal females)	X												
Randomisation			X										
Trial drug administration[10]			X	X	X								
Meals[9]		X	X	X	X	X	X	X	X				
**Safety and tolerability:**													
Vital signs[1]	X	X	X	X	X	X	X	X	X	X	X	X	X
12-lead ECG[2]	X	X	X	X	X	X	X	X	X	X	X	X	X
24-hour Holter ECG	X												
Telemetry[6]			X	X	X								
BMI calculation	X	X[8]											
Physical examination[3]	X	X	X	X	X	X	X	X	X	X			X

**Procedure**	**Screening (D-22 to D-2)**	**D-1**	**D1**	**D2**	**D3**	**D4**	**D5**	**D6**	**D7[11] Sentinel Group only**	**D8**	**D15**	**D22**	**Last visit D30 (±1)**
Biochemistry, Haematology, Coagulation[4]	X	X	X	X[12]	X[12]	X	X	X	X	X	X	X	X
Urinalysis	X	X	X		X		X		X	X	X		X
**Pharmacokinetics:**													
PK sampling – PYR[5]			X	X	X	X		X		X	X	X	
PK sampling – PQP[5] (including e.g. N-oxidated metabolite)			X	X	X	X		X		X	X	X	
**Pharmacogenomics:**													
CYP polymorphism			X										

1. Vital signs (supine blood pressure, heart rate and respiratory rate) will be measured after rest of at least five minutes in supine position. Tympanic body temperature will be taken at each time-point (resting in supine position is not required for body temperature). Standing blood pressure and heart rate will be performed one minute after standing to assess for orthostatic change at screening and on Day -1, Day 1 and Day 3. See Table 6 for Time-points.
2. 12-lead ECG will be measured in triplicate after a rest period of at least ten minutes in supine position. See Table 6 for time-points.
3. Full physical examination at screening and last visit only. Symptom-directed physical examinations at other time-points.
4. See Table 13 for details of laboratory parameters to be measured.
5. Plasma samples for PQP and blood samples for PYR. See Table 6 for time-points.
6. Continuous 12-lead telemetry will be recorded from at least one hour pre-dose on Day 1 to 24 hours post-dose on Day 3.
7. Update only on Medical History at admission.
8. On Day -1, only weight will be collected and height from screening will be used for BMI calculation.
9. Standard meals will be served at standard unit times. On Days 1, 2 and 3 the first meal will be lunch given four hours post-dose. Meals will be optional on the discharge day.
10. Dosing will be carried out in fasted state on the morning of each dosing day (Days 1, 2 and 3), firstly with PYR/ placebo tablets, followed by PQP/ placebo tablets.
11. Participants in the sentinel group will remain in-house until Day 7. If there are no clinically concerning safety signals, the remaining participants subsequently dosed (from the main group) will remain in-house until Day 5.
12. On Day 2 and Day 3, only the following safety blood tests will be measured: AST, ALT, bilirubin.

Note: COVID-19 tests will be carried out at regular intervals, prior to entry in the unit and throughout the residential period as per the latest RPL COVID-19 Infection Control Guidelines and pre-entry algorithms.

Abbreviations: AE: Adverse Event; BMI: body mass index; D: Day (where Day 1 is the first day that IMPs are administered); ECG: electrocardiogram; FSH: Follicular Stimulating Hormone; PK: pharmacokinetic; PQP: piperaquine; PYR: pyronaridine.

Table 6 Schedule of Assessments (continued)

Trial Day	Time (h)	PK sampling[1]	12-lead ECG[2]	Vitals[3]	Meals[4,5]
D1	-1.5		X		
	-1		X		
	-30 mins	X	X	X	
	1	X	X	X	
	2	X	X	X	
	3	X	X		
	4	X	X		X
	5	X	X	X	
	6	X	X		
	7	X	X		
	8	X	X	X	
	10				X
	12	X	X		X
D2	-1.5		X		
	-1		X		
	-30 mins	X	X	X	
	4	X	X	X	X
	6	X	X	X	
	10				X
	12	X	X	X	X
D3	-1.5		X		
	-1		X		
	-30 mins	X	X	X	
	-5 mins		X		
	1	X	X	X	
	2	X	X	X	
	3	X	X		
	4	X	X		X
	5	X	X	X	
	6	X	X		
	7	X	X		
	8	X	X	X	
	10				X
	12	X	X		X

1. Plasma samples for PQP and blood samples for PYR.
2. 12-lead ECG will be measured in triplicate after a rest period of at least ten minutes in supine position.
3. Vital signs will be measured after rest of at least five minutes in supine position. Standing vitals will be performed after one minute in the upright standing position, to assess for orthostatic change, on Screening, Day -1, and on Day 1 and Day 3 at pre-dose (-30 min) and post dose at 5h.
4. On dosing days, participants will fast from all food and drink (excluding water) at least three hours pre-dose and four hours post-dose. Participants may drink water until one hour before dosing and may re-commence water one hour subsequent to dosing.
5. Four-hour procedures to be completed prior to lunch being given

Abbreviations: D: day; ECG: electrocardiogram; h: hours (where hour 0 is the time at which the first IMP is administered); mins: minutes; PK: pharmacokinetic.

3.2 Order of procedures, scheduling time windows and protocol deviations

Scheduling time windows:

Priorities, order of procedures, scheduling time windows, and deviations from scheduled time and scheduling time windows.

Priorities:

When the protocol's Schedule of Assessments requires that multiple procedures occur at the same timepoint, PK blood sampling takes priority and is to be scheduled at the exact time point defined in the schedule of assessments.

Furthermore, the trial design also incorporates intensive ECGs as part of the analysis planned to determine the effect of the study drug(s) (either alone or in combination) on ECG parameters. Therefore, scheduled ECGs are also important for this trial and should take priority after PK sample.

Order of procedures:

When the Schedule of Assessments requires that multiple procedures occur at the same timepoint, the order of the procedures is as specified below.

Screening:

Screening can occur over several days if required. There is no set order of procedures required if this occurs. If all procedures are performed on the same day and where timepoints collide, then blood samples must be taken after vital signs (including standing vitals) and ECGs, to avoid the process of venepuncture affecting the vital sign readings and the ECG measurements.

Order of procedures for D-1:

1. ECGs (measured after rest of at least 10 minutes in supine position)
2. Vital Signs (measured after rest of at least 5 minutes in supine position and after rest of at least 1 minute in standing position).

Order of procedures pre-dose, during in-house period:

1. ECGs (measured after rest of at least 10 minutes in supine position)
2. Vital Signs (measured after rest of at least 5 minutes in supine position and after rest of at least 1 minute in standing position)
3. Blood Samples (PK [exactly on time] > Biochemistry, Haematology and Coagulation)
4. PGx sample.

Order of procedures post-dose, during in-house period:

1. ECGs (measured after rest of at least 10 minutes in supine position)
2. Vital Signs (measured after rest of at least 5 minutes in supine position and after rest of at least 1 minute in standing position)
3. Blood Samples (PK [exactly on time] > Biochemistry, Haematology and Coagulation)

4. Food (first meal to be served a minimum of four hours post-dose on Days 1, 2 and 3).

Order of procedures during outpatient visits:
1. ECGs (measured after rest of at least 10 minutes in supine position)
2. Vital Signs (measured after rest of at least 5 minutes in supine position and after rest of at least 1 minute in standing position)
3. Blood Samples (PK [exactly on time] > Biochemistry, Haematology and Coagulation).

Meals will be provided in the order below on each dosing day (Days 1, 2 and 3), following the standard timings of the research unit:
1. Lunch (at least 4 hours post dose)
2. Dinner (10 hours post dose)
3. Snack (12 hours post dose).

Meals for the non-dosing residential days will be served as below, following the standard timings of the research unit:
1. Breakfast (following the morning procedures)
2. Lunch
3. Dinner
4. Snack.

Scheduling time windows:

Considering priorities and order of procedures, the design of the master treatment schedule (MTS) requires scheduling time windows for those tasks that occur at the same protocol time point but need to be performed before or after the priority task. The permitted time windows are set out in the Data Handling Protocol for scheduling time windows (DHP STW).

Clinical assessments should be performed exactly at the time prescribed in the MTS.

Deviations:
- Deviation from scheduled time in MTS: If the time of clinical assessment deviates from the time prescribed in the MTS, a clinical comment should be made in the appropriate source data/CRF space that a deviation from the MTS occurred. The following deviations from the MTS do not require a comment:

  All Procedures ± 2 mins

- Deviation from scheduling time window in DHP STW: If the time of clinical assessment deviates from the scheduling time window in the DHP STW, this will be considered a protocol deviation. These deviations will be identified, classified (minor, major, critical), and managed in accordance with the DHP STW.

3.3 Adaptive design

This trial incorporates the use of an adaptive design. Trial specific adaptive features and their limits are described in Table 7.

Adaptive features may be implemented only with the approval of the sponsor. Implementation of adaptive features affecting whole dose groups or cohorts, or the entire trial, will be documented in a non-substantial amendment.

The exceptions to this are those adaptive features in Table 7 that relate to individual participant safety. These may be implemented at the discretion of the Investigator and recorded in that participant's source data.

Table 7: Adaptive protocol features

Adaptive Study Design Areas	Features	Limits
**Sentinel/ subgroup dosing**	1. Cohorts may be split into sub-groups.	I. A mandatory sentinel dosing strategy of dosing four participants from treatment arm 1, and two participants from each of treatment arms 2, 3 and 4, will be used. There will be a total of ten participants in the sentinel dosing. The remaining participants can be dosed if safety and tolerability data up to 15 days post first dose is acceptable.<br>II. The participants in the sentinel group will all be dosed in parallel. If they are successfully dosed (i.e. it is deemed safe to dose the other participants), the remaining 30 participants may all be dosed at the same time, in parallel, or they may be divided into two or more dosing groups, with the groups being dosed on different days. Each dosing group would contain a minimum of two participants from each of the four treatment arms, to maintain blinding and avoid bias (See Table 12).
**Flexible Cohort Sizes**	2. Withdrawn participants can be replaced at the discretion of the sponsor and PI.<br>3. Replacement participants may be enrolled in an ongoing treatment group or dosed together as a group or dosed separately, except for	I. The maximum extension is 100 % of the original treatment group.<br>II. Trial specific AR rules (Section 4.4) apply.

Adaptive Study Design Areas	Features	Limits
	those participants in the sentinel group.<br>4. The number of participants in a treatment group can be extended to gather further information.	
**In-house duration and visit numbers**	5. Participants in the sentinel group will remain in-house until Day 7. Participants in the main group may be discharged on Day 5, based on the available safety and tolerability data from the sentinel group and as decided by the SRC.<br>6. The in-house stay or follow-up period may be prolonged or shortened if:<br>a. it is considered clinically necessary to prolong the in-house stay by the PI/delegate for individuals on a case-by-case basis<br>b. the SRC considers it necessary from a safety/tolerability point	I. A maximum extended in-house or follow-up period cannot be pre-defined as the extension will be as long as necessary to ensure the safety of the individual participant(s).<br>II. The maximum extended in-house or follow-up period for trial will be based on evolving safety and tolerability data.<br>III. Alterations in duration of in-house stay or follow-up need to ensure that sufficient data are obtained to fulfil the trial objectives and be a reflection of the established safety and tolerability PK profile up to the decision-making time-point.
**Samples and Assessments (safety)**	7. Additional safety assessments may be performed on an individual participant if it is considered clinically necessary by the PI for individuals on a case-by-case basis.<br>8. Additional safety assessments (including but not limited to laboratory safety samples, vital signs and ECGs) may be added to the Schedule of Assessments at additional time-points for an upcoming treatment arm if the SRC considers it necessary from a safety/tolerability perspective, on the basis of evolving data.<br>9. Additional safety assessments may refer to either:<br>a. an increased number of the same safety assessments planned in the existing Schedule of Assessments.<br>b. additional parameters (specific tests) on assessments already scheduled e.g. troponin tests on safety blood samples.<br>c. additional safety tests requiring additional blood/urine sample collections or other clinical	I. Trial specific maximum blood volume (470 ml) will not be exceeded (Section 7.12).<br>II. A maximum for individuals will be determined on a case-by-case basis and cannot be pre-defined, because investigations will be performed as necessary to ensure the safety of the individual participants.<br>III. If additional safety assessments or parameters are required for upcoming treatment arms/cohorts and have a similar risk profile to those planned in this trial protocol, they will be documented in a non-substantial amendment. Additional safety assessments required for upcoming treatment arms/cohorts that are more invasive or have a different risk profile to those in this current protocol must be detailed in a substantial amendment.

Adaptive Study Design Areas	Features	Limits
	procedures e.g. ultrasound scans.<br>10. Specialist referrals (e.g. to a cardiologist) may be made (and may include all relevant assessments and investigations) if it is considered clinically necessary by the PI or Sponsor or SRC for individuals on a case-by-case basis.<br>11. The timing of safety assessments including but not limited to laboratory safety samples, vital signs and ECGs may be adjusted in accordance with evolving data and dosing schedule.	IV. Alterations in timing of the safety assessments need to be a reflection of the established safety/tolerability and PK profile up to the decision-making time-point.
**Samples and Assessments (PK)**	12. Additional blood PK assessments may be taken in accordance with evolving data and dosing schedule. This includes those for the analysis of metabolites.<br>13. Additional PK samples may be taken to get a better understanding of PK profile related to safety e.g. in the event of LFT changes for an individual participant during the trial it may be necessary to take additional PK samples.	I. Study specific maximum blood volume will not be exceeded (Section 7.12).
**Samples and Assessments (ECG assessments)**	14. Additional PK samples paired with ECGs may be taken in the case of QT/QT_c interval or LFT changes in an individual participant.<br>15. If the meal-times need to be changed, ECG times may also be changed to maintain the sampling schedule required to capture the food effect for the confirmation of assay sensitivity.<br>15. Telemetry data gathered during the trial may be used in the exploratory analysis of drug-related QT/QT_c interval changes.	I. To confirm assay sensitivity, a minimum of four postprandial time points will be recorded in triplicate between 1 4h after the start of the meal.
**Screening**	16. Screening assessments, including Holter ECG recordings performed at Richmond Pharmacology Ltd on participants screened (but not randomised) for another study can be used for this study to avoid unnecessary tests.	I. The assessments must meet protocol criteria (e.g. the method to be used).<br>II. The assessments must be performed within the protocol defined screening window.<br>III. The Holter ECG recordings are valid for a period of three months.

Adaptive Study Design Areas	Features	Limits
**Optional analysis**	17. Exploratory analysis of CYP isoforms and transporters may be performed in individual participants, selected or all treatment groups.<br>18. The CYP isoforms and transporters can be adjusted according to emerging data.<br>19. Exploratory analysis of PK parameters of PQP metabolites (e.g. N-oxidated metabolite) and metabolites to parent drug ratios may be performed in individual participants, selected or all treatment groups. This may be reported separately from the CSR.<br>20. ECG analysis for the purpose of intensive cardiac assessments may be performed. Telemetry data gathered during the study may be used in the optional analysis.	IV. Any results of optional analysis will be reported; however, they may be reported separately and not in the Clinical Study Report.

Abbreviations: AR: Adverse Reaction; ECG: Electrocardiogram; h: hour; PK: pharmacokinetic.

3.4 Rationale for trial design, doses and control groups

This trial will evaluate the safety and tolerability profile of the approved regimen of PYR and PQP for the treatment of uncomplicated malaria when co-administered once daily for three days. It is anticipated that these same doses will be used in the future, when used for seasonal malarial chemoprevention. The trial is randomised and double blinded in order to minimise bias and includes matched placebo for both IMPs to facilitate identification of effects related to treatment, rather than situational- or procedure-related effects.

3.4.1 Justification for the selected dose level

Based on efficacy data provided to support registration for malaria treatment, PYR and PQP are long acting antimalarials, which extended half-lives shall confer a protection from re-infection for one month with the standard three-day regimen. Additionally, DHA-PQP was associated with a protective effect of over 75 %. It is therefore assumed that a monthly treatment (for three to four consecutive months during the malaria season) based on the registered doses should achieve adequate protective efficacy (i.e. in people who are either parasite-free or have circulating parasites but are asymptomatic).

The approved adult malaria treatment doses of PYR and PQP have therefore been chosen for this trial because these are the adult equivalent doses of the doses that would be used in the treatment of children. The dosage chosen corresponds to the dosages of PYR and PQP used in current combination antimalarials which are already licenced and known to be well tolerated.

PYR will be given at a total daily dose of 540 mg for participants with a body weight between 50 and <65 kg and a total daily dose of 720 mg for participants with a

body weight superior or equal to 65 kg. PQP will be given at a total daily dose 960 mg for participants with a BW between 50 and <75 kg and a total daily dose of 1280 mg for participants with a BW equal or superior to 75 kg. See Table 1 above for weight-based dose calculations.

3.4.2 Choice of participants for trial

Male and female participants aged between 18 and 45 years inclusive considered healthy for their age are planned to be recruited in the trial. The selection criteria are defined such that participants selected for participation in the trial are known to be free from any significant illness which potentially could confound the trial results or put the participant at risk. Healthy participants are also unlikely to require concomitant treatments which could interfere with the trial drugs. All efforts will be made to ensure a reasonable male:female gender balance throughout the trial.

Considering that the African population is at considerably higher risk of contracting malaria and will be the target population for future prophylaxis trials with this new combination, the current trial will only recruit black participants of Sub-Saharan African origin (defined as participants whose parents are both black and are of Sub-Saharan African origin).

3.4.3 Route and rate of administration

PYR, PQP and matched placebo tablets will be administered via the oral route in the fasted condition. Fasting is required for PQP administration in order to minimize the QT_c prolongation induced by the medicine. As no clinically relevant food effect has been observed with PYR, the drug can also be given in a fasted state in order to achieve efficacious exposures. Initially, PYR or matched PYR placebo will be administered. Immediately subsequently, PQP or matched PQP placebo will be administered. Participants will fast of all food and drink (except water) for three hours pre-dose and four hours post-dose. Water may be consumed up to one hour prior to dosing and re-starting one hour subsequent to dosing.

3.4.4 Precautions to be applied for dosing between participants in sentinel group and participants in the main group

A mandatory sentinel dosing strategy of dosing ten participants will be used to provide safety assessment. The sentinel group will consist of four participants from treatment arm 1 and two participants from each of treatment arms 2, 3 and 4. Blinded safety data up to Day 15 in the sentinel group will be reviewed by a Safety Review Committee (SRC) prior to dosing the 30 remaining participants. If safety and tolerability is acceptable from participants within the sentinel group, the remaining participants of each treatment arm may be dosed.

3.4.5 Monitoring and communication of Adverse Events/Reactions

AEs will be continuously monitored throughout the trial from the signing of the ICF until the last follow-up assessment. Each AE reported will be assessed by a trained Research Physician (RP) who will ensure that the event is dealt with as appropriate based on clinical need, trial protocol, study operations manual and Richmond

Pharmacology Standard Operating Procedures (SOPs). AEs will be documented in the participants' source Case Report Forms (CRFs) and reviewed regularly by the RPs and the Investigator.

If any information relating to the IMPs in this trial becomes available after the submission of a final protocol to the Competent Authority which may impact on the conduct of the trial, including but not limited to the risk and benefit evaluations underpinning approvals and participant's consent, MMV shall notify RPL in writing as soon as practically possible and the parties will agree, in writing, what steps need to be taken if any.

3.4.6 Investigator site facilities and personnel

This trial will be conducted in a specialised early phase Clinical Pharmacology Unit with onsite resuscitation equipment and medication, in addition to access to an acute hospital with Critical Care facilities, thus ensuring direct access to equipment and staff for resuscitating and stabilising participants in acute medical conditions and emergencies. The trial is conducted by an experienced PI and well trained medical and technical staff with ample experience in the conduct of early phase clinical trials.

The trial is designed to closely monitor, treat and communicate potential expected Adverse Reactions as well as potential unexpected adverse events.

4. DECISION-MAKING, RULES AND LIMITS

4.1 Definitions

'Continuation' in the context of this protocol refers to either (1) continuing dosing in an individual participant or (2) continuing dosing in the trial.

4.2 Rules and limits governing decision-making

The criteria and rules that will govern PI/SRC decisions are:

1. minimum data requirements (Table 8)
2. Adverse Reaction rules (Section 4.4)
3. Adaptive features and their limits (Table 7).

The dose continuation decision requirement for the trial is outlined in Table 8 below.

Table 8: Minimum data requirements to proceed from sentinel dosing to dosing remaining participants

Decision making time point	Person or body making the decision	Minimum data to be looked at (in accordance with the CSP)	Method of documentation of decision and/or communication to sponsor (if applicable)
Continuation from the sentinel group to the remainder of the participants in all treatment arms.	SRC	The SRC will review all available safety and tolerability data collected up to Day 15. All clinically significant findings will be taken into account by the SRC when determining whether the remaining participants can be dosed as planned.<br>Safety data from minimum of three participants in the combination PYR-PQP sentinel arm is required. Safety data from a minimum of one participant in each of the sentinel IMP-placebo arms is required for SRC.	The SRC will document the decision on the dose continuation approval form.

Abbreviations: CSP: Clinical Study Protocol; SRC: Safety Review Committee

4.3 Safety Review Committee

The SRC will consist of, as a minimum:

- Principal Investigator (RPL) or delegate
- MMV, Study Medical Director or delegate
- Study Medical Monitor or delegate.

Further internal or external experts such as a pharmacokineticist and/or a statistician, may be consulted by the SRC as necessary. Any additional information, if required, will be included in the SOM and the SRC Charter.

4.3.1 SRC Meeting

The SRC will meet to review sentinel group data up to and including Day 15 of the final participant in the sentinel group (Table 8).

There is an option to have ad-hoc SRC meetings to discuss urgent issues should the need arise.

Prior to each SRC meeting, an interim safety report will be prepared, presenting the relevant safety and tolerability data. These will be signed by the investigator.

Initially the data will be reviewed blinded. If the SRC consider it necessary due to a safety concern, either individual participants or the entire cohort may be unblinded to enable their decision-making. For non-emergency unblinding, before breaking the code, the potential decisions and actions should be determined and documented.

These decisions will be signed by one of the sponsor's representatives and by the investigator. For logistical reasons, the signature of the sponsor or investigator may be communicated via email.

The decision of the SRC will be taken in consensus between the members of the SRC. If consensus cannot be reached, then the most cautious approach will proceed.

4.4 Adverse Reaction (AR) rules

An AR is any AE where there is a reasonable possibility of it being related to the IMP(s). ARs will be classified in accordance with Sections 8.1 and 8.2 to support standardised recording and reporting.

- Seriousness will be assessed using the criteria in Section 8.1.
- Severity and causality will be assessed using the criteria in Section 8.2.

Seriousness and severity are assessed independently. 'Severity' characterises the intensity of an AE. 'Serious' is a regulatory definition and serves to define and trigger regulatory reporting obligations.

Seriousness and severity grade both have consequences when applying AR rules. Seriousness of an AR always overrides the severity grade and the rules for Serious AR should be applied, irrespective of severity grade.

These rules will only apply to AEs/SAEs where there is a reasonable possibility of relationship to the IMP (i.e. ARs and SARs). Every AE will be assessed in the following order:

1) the impact on the individual participant, e.g. whether IMP administration can be continued
2) whether blinding can continue for the individual
3) a) (if the participant is unblinded) the impact on the treatment arm the individual participant is part of, e.g. in the following circumstances:
    - if a treatment arm is due to receive further doses (i.e. a dose on the following day), whether that treatment arm can receive further doses as per the dosing schedule
    - if the individual(s) is/are part of the sentinel group, the impact on the successive participants of that treatment arm – i.e. whether the remaining subjects can be dosed or not.

3) b) (if the participant remains blinded) the impact on all remaining participants in the study, e.g. in the following circumstances:

- if participants are due to receive further doses (i.e. a dose on the following day), whether the remaining participants can receive further doses as per the dosing schedule
- if the individual(s) is/are part of the sentinel group, the impact on all successive participants – i.e. whether the remaining cohort can proceed or not.

4) the impact on continuation or suspension of the overall study.

The individual (decision 2), within-cohort (decision 3) and the trial progression (decision 4) rules are shown in Table 9 and Table 10 below.

Table 9: Individual rules (decision 1)

**Adverse Events Related to Trial Drug**	**Action[a]**
Moderate (Table 14) except QT interval prolongation, haematology, and liver function, for which rules are specified below)	Trial drug administration may be continued, delayed, temporarily suspended or discontinued in accordance with Investigator's clinical judgment
Moderate LFT increases: ALT or AST value >3 x and <5 x ULN	
QT interval prolongation:<br>A prolongation of the uncorrected QT interval of greater than 500 ms, using consistent, technically valid triplicate ECG	Trial drug administration will be discontinued.
Haematology:<br>a) Hb drop of >25 % from baseline on Day-1 (only for values below the laboratory reference range), or to an absolute value of <100 g/L<br>b) Platelet count drop of > 25% from baseline on Day-1 (only for values outside the laboratory reference range), or absolute value of <80 x 10^9/L<br>c) Clinically significant neutrophil drop[b]	
Severe (Table 14) except liver function, for which rules are specified below.	
ALT or AST value >3 x and <5 × ULN, and symptomatic or ALT or AST value >5 × ULN +/- symptoms [b]	
Serious (fatal or life-threatening)	
Serious LFT increase: ALT or AST value >3 × ULN together with bilirubin >1.5 x ULN, without other evidence of cholestasis	

[a]Actions are only relevant for dosing regimens where additional doses are planned for administration.
[b]Symptoms include fatigue, nausea, vomiting, right upper quadrant pain or tenderness, fever, rash and/or eosinophilia (eosinophil percent or count above the ULN). Abbreviations: ALT: alanine aminotransferase; AST: aspartate aminotransferase; ECG: Electrocardiogram; Hb: Haemoglobin; ms: milliseconds; QT: QT interval; ULN: upper limit of normal.
*Investigator judgement will be used to determine which of the pre-dose measurements are considered the baseline Where possible, the baseline value should be the one that was taken at the closest time of day to the samples being assessed for change, to account for diurnal variation (for example, the D-1 morning blood samples)

Table 10: Cohort and trial rules (decisions 3 and 4)

<table>
<tr><th>Adverse Events Related to Trial Drug</th><th>Reversibility</th><th>Number of Participants</th><th>Action[a]</th></tr>
<tr><td>Moderate LFT increases:
ALT or AST value >3 x ULN and <5 x ULN</td><td>ALT/AST improves to <3 x ULN in all participants</td><td>≥50 % of sentinel group
≥50 % of a treatment arm (if unblinded)
≤5 participants of the remaining main cohort after sentinel dosing</td><td>Trial drug administration may continue</td></tr>
<tr><td>Moderate LFT increases:
ALT or AST value >3 x ULN and <5 x ULN</td><td>ALT/AST does not improve to <3 x ULN in all participants</td><td>≥50 % of sentinel group
≥50 % of a treatment arm (if unblinded)
≤5 participants of the remaining main cohort after sentinel dosing</td><td rowspan="4">Further dosing of the remaining participants of the relevant treatment arm (if unblinded) or trial (if blinded) will be suspended. Re-commencement of dosing requires regulatory approval via a substantial amendment[a]Further dosing of the remaining participants of the relevant treatment arm (if unblinded) or trial (if blinded) will be suspended. Re-commencement of dosing requires regulatory approval via a substantial amendment[a]</td></tr>
<tr><td>QT interval prolongation:
A prolongation of the uncorrected QT interval of greater than 500 msec, using consistent, technically valid triplicate ECG</td><td>N/A</td><td>≥2</td></tr>
<tr><td>Haematology:
a) Hb drop of >25 % from baseline on Day -1 (only for values below the laboratory reference range), or to an absolute value of <100 g/L
b) Platelet count drop of >25 % from baseline on Day -1 (only for values below the laboratory reference range), or absolute value of <80 x 10^9/L
Clinically significant neutrophil drop[b]</td><td></td><td>≥2</td></tr>
<tr><td>Severe Adverse Reaction (Table 14)</td><td></td><td>≥2</td></tr>
</table>

Adverse Events Related to Trial Drug	Reversibility	Number of Participants	Action[a]
Severe LFT increase: ALT or AST value >3 × ULN and symptomatic/ ALT or AST value >5 × ULN +/- symptoms[b]		≥2	
Serious Adverse Reaction (fatal or life-threatening)		≥1	
Serious LFT increase: ALT or AST value >3 × ULN, together with Bilirubin >1.5 ULN without other evidence of cholestasis		≥1	

[a]A substantial amendment may include an amendment to the protocol, the ICF, or other trial related documents as appropriate.

[b]Symptoms include fatigue, nausea, vomiting, right upper quadrant pain or tenderness, fever, rash and/or eosinophilia (eosinophil percent or count above the ULN).

Abbreviations: ALT: alanine aminotransferase; AST: aspartate aminotransferase; ULN: upper limit of normal; QT: QT interval; ms: milliseconds; ECG: electrocardiogram; Hb: haemoglobin; SOC: System Organ Class; WCC: white cell count.

The investigators judgement will be used to determine which of the pre-dose measurements are considered the baseline Where possible, the baseline value should be the one that was taken at the closest time of day to the samples being assessed for change, to account for diurnal variation (for example, the D-1 morning blood samples).

5. SELECTION AND WITHDRAWAL OF PARTICIPANTS

5.1 Number and Source of participants

Forty (40) participants will be recruited to this trial and will be randomised to one of the four treatment arms in a 2:1:1:1 ratio as described below:

- Treatment 1 (N=16): PYR + PQP
- Treatment 2 (N=8): PYR + placebo for PQP
- Treatment 3 (N=8): placebo for PYR + PQP
- Treatment 4 (N=8): placebo for PYR + placebo for PQP.

Participants enrolled in this trial will be members of the community at large. The recruitment advertisements may use various media types (e.g. radio, newspaper, the clinical site website and participant database).

5.2 Replacement participants

Additional participants may be recruited to replace any discontinued participants, in order to ensure that 40 participants complete the trial. This will require agreement with the trial sponsor.

5.3 Inclusion Criteria

Participants must meet all of the following criteria to be eligible for enrolment in this trial:

1. Black male or female, of Sub-Saharan African origin (defined as participants whose parents are both black and are of Sub-Saharan African origin) aged ≥18 years to ≤45 years at the date of signing informed consent. This inclusion criterion will only be assessed at the screening visit
2. Healthy as defined by:
   - the absence of clinically significant illness and surgery within four weeks prior to dosing. Participants vomiting within 24 hours pre-dose will be carefully evaluated for upcoming illness/disease. Inclusion pre-dosing is at the discretion of the Investigator
   - the absence of clinically significant history of neurological, endocrine, cardiovascular, respiratory, haematological, immunological, psychiatric, gastrointestinal, renal, hepatic and metabolic disease
3. Participants must agree to use the following contraceptive requirements for the applicable duration:
   - <u>Female participants of non-childbearing potential (WNCBP):</u> Defined as either postmenopausal (evidence of menopause based on a combination of amenorrhea for at least one year and increased serum follicle-stimulating hormone (FSH) level [>30 IU/L]), or surgical sterilisation (evidence of hysterectomy and/or bilateral oophorectomy)

     CONTRACEPTION REQUIRED: None
   - <u>Female participants of childbearing potential (WOCBP) who anticipate being sexually active with a male during the trial (from one complete</u>

menstrual cycle prior to the first IMP administration until 110 days (slightly less than 16 weeks) after the last IMP administration):

CONTRACEPTION REQUIRED: Highly effective contraception must start one complete menstrual cycle prior to the first day of dosing and continue until 110 days (slightly less than 16 weeks) after the last IMP administration. Highly effective contraception methods for WOCBP include:

- combined (i.e. oestrogen- and progestogen-containing) hormonal contraception associated with inhibition of ovulation:
    - oral
    - intravaginal
    - transdermal
- progestogen-only hormonal contraception associated with inhibition of ovulation:
    - oral
    - injectable
    - implantable
- intrauterine hormone-releasing system (IUS)
- intrauterine device (IUD)
- bilateral tubal occlusion
- infertile male partner (e.g. vasectomised, permanently sterile following bilateral orchidectomy, or any other documented cause of infertility)

- Female participants of childbearing potential (WOCBP) who agree to remain abstinent for the duration of the trial (from one complete menstrual cycle prior to the first IMP administration until 110 days (slightly less than 16 weeks) after the last IMP administration):

  CONTRACEPTION REQUIRED: Abstinence (sexual abstinence is considered a highly effective method only if defined as refraining from heterosexual intercourse during the entire period of risk associated with the trial treatments. The reliability of sexual abstinence needs to be evaluated in relation to the duration of the clinical trial and the preferred and usual lifestyle of the participant. Calendar, symptothermal and post-ovulation methods of contraception are not considered to be equivalent to abstinence)

- Male participants, who agree to remain abstinent for the duration of the trial (from first IMP administration until 110 days (slightly less than 16 weeks) after the last IMP administration):

  CONTRACEPTION REQUIRED: Abstinence (sexual abstinence is considered a highly effective method only if defined as refraining from heterosexual intercourse during the entire period of risk associated with the trial treatments. The reliability of sexual abstinence needs to be

evaluated in relation to the duration of the clinical trial and the preferred and usual lifestyle of the participant)

If the situation changes post-dose during the trial, participants must use a condom with or without spermicide.

- Male participants, who anticipate being sexually active during the trial period (from first IMP administration until 110 days (slightly less than 16 weeks) after the last IMP administration) with a woman who is either a WOCBP, a woman who is pregnant and/or breast feeding:

  CONTRACEPTION REQUIRED: From the first day of dosing until the end of the systemic exposure of the trial drug. Acceptable methods are:

  - male condom with or without spermicide
  - infertile male (e.g. vasectomised, permanently sterile following bilateral orchidectomy, or any other documented cause of infertility)

4. Participants must agree not to donate sperm or ova from the time of the first administration of trial medication until three months after the end of the systemic exposure of the trial drug
5. Participants must have a body weight of 50 kg or greater and a BMI between 18.0 kg/m² - 28.0 kg/m² (inclusive) at screening
6. Satisfactory medical assessment with no clinically significant or relevant abnormalities as determined by medical history, physical examination, vital signs, 12-lead ECG and clinical laboratory evaluation (haematology, biochemistry, coagulation, and urinalysis) that is reasonably likely to interfere with the participant's participation in or ability to complete the trial as assessed by the Investigator
7. Ability to provide written, personally signed, and dated informed consent to participate in the trial, in accordance with the ICH Good Clinical Practice (GCP) Guideline E6 (R2) (2016) and applicable regulations, before completing any trial-related procedures
8. An understanding, ability, and willingness to fully comply with trial procedures and restrictions.

5.4 Exclusion Criteria

Participants will be excluded from enrolment in this trial if they meet any of the following criteria:

1. Current or recurrent disease (e.g. cardiovascular, haematological, neurological, endocrine, immunological, renal, hepatic or gastrointestinal or other conditions, including cholecystectomy or gastrectomy) that could affect the action, absorption, distribution, metabolism or excretion of PYR or PQP or could affect clinical assessments or clinical laboratory evaluations
2. Any history of seizures or epilepsy
3. Any history of photosensitivity
4. Any documented retinopathy
5. History of malaria in the previous two years

6. A score of 20 or more on the Beck Depression Inventory, and/or a response of 1, 2 or 3 for item 9 of this inventory (related to suicidal ideation) [26].
7. Current or relevant history of physical or psychiatric illness that are not stable or may require a change in treatment, or use of prohibited therapies during the trial, that make the participant unlikely to fully comply with the requirements of the trial or to complete the trial, or any condition that presents undue risk from the investigational product or trial procedures
8. Any other significant disease or disorder which, in the opinion of the Investigator, may either put the participant at risk because of participation in the trial, or may influence the result of the trial or the participant's ability to participate in the trial
9. The history or presence of any of the following cardiac conditions: known structural cardiac abnormalities; family history of long QT syndrome; cardiac syncope or recurrent, idiopathic syncope; exercise-related clinically significant cardiac events
10. Any clinically significant abnormalities in rhythm, conduction or morphology of resting ECG or clinically important abnormalities that may interfere with the interpretation of QT_c interval changes. This includes participants with any of the following (at screening or Day -1):
    - sinus node dysfunction
    - clinically significant PR >220 msecs (PQ) interval prolongation
    - second- or third-degree atrioventricular (AV) block
    - sustained cardiac arrhythmia's including (but not limited to) atrial fibrillation or supraventricular tachycardia, or any symptomatic arrhythmia, with the exception of isolated extra-systoles
    - abnormal T-wave morphology which may impact on the QT/QT_c assessment
    - QT interval corrected using the Fridericia's formula (QT_cF) >450 ms (males and females)
    - any other ECG abnormalities in the standard 12-lead ECG and 24-hour 12 lead Holter ECG or an equivalent assessment which in the opinion of the Investigator will interfere with the ECG analysis

    Participants with borderline abnormalities may be included if the deviations do not pose a safety risk, and if agreed between the appointed cardiologist and the PI.
11. Has vital signs consistently outside of normal range at screening or Day -1. Acceptable normal range is as follows:
    - supine HR 40 - 100 bpm (after at least five minutes of supine rest)
    - supine blood pressure (after at least five minutes of supine rest):
        - systolic blood pressure: 90 - 140 mmHg
        - diastolic blood pressure: 40 - 90 mmHg
12. Has a positive test for Hepatitis B surface Antigen (HBsAg), Hepatitis C Antibody (HCV Ab), or Human Immunodeficiency Virus Antibody (HIV Ab) at screening

13. Has total bilirubin, ALT or AST consistently >ULN at screening (up to two repeats may be taken during the screening period; participant may be included if two out of the three total results are ≤ULN), or has total bilirubin, ALT or AST>ULN on Day -1 (mild variations from baseline may be allowed if considered not clinically significant by the Investigator)
14. Has a haemoglobin, platelet count, total white blood cell count, lymphocyte or monocyte count less than Lower Limit of Normal (LLN) (up to two repeats may be taken during the screening period and on Day -1 (participants may be included if two out of the three total results are greater or equal to LLN), at screening. Where there is a clear diurnal effect on the result participants may be included if variations are considered not clinically relevant by the Investigator
15. Any other abnormal findings on vital signs, ECG, physical examination or laboratory evaluation of blood and urine samples that the Investigator judges as likely to interfere with the trial or pose an additional risk in participating
16. Positive test results for alcohol or drugs of abuse at screening or Day -1
17. Female participants who are pregnant (including a positive serum pregnancy test at screening or on Day -1) or breastfeeding
18. Male participants with a female partner(s) who is (are) pregnant or lactating at screening or on Day -1, or is (are) expected to be during the trial period
19. History or clinical evidence of substance and/or alcohol abuse within the two years before screening. Alcohol abuse is defined as regular weekly intake of more than 14 units (for both males and females), using the following National Health Service (NHS) alcohol tracker https://www.nhs.uk/oneyou/for-your-body/drink-less/know-your-alcohol-units/
20. Treatment with an investigational drug within 90 days or five half-lives preceding the first dose of trial medication (whichever is the longer)
21. Use of tobacco in any form (e.g. smoking or chewing) or other nicotine-containing products in any form (e.g. gum, patch, electronic cigarettes) within six months prior to the planned first day of dosing
22. Has used any medication (see list for reference: https://www.fda.gov/drugs/drug-interactions-labeling/drug-development-and-drug-interactions-table-substrates-inhibitors-and-inducers#transporter) that is either a moderate or strong inhibitor or inducer of CYP3A4 within 30 days or five half-lives (whichever is longer) prior to the planned first day of dosing
23. Additionally, participants must have not consumed other substances known to be potent inhibitors or inducers of CYP3A4 system such as grapefruit or cranberry juice-containing products in the 30 days before the planned first IMP administration
24. Has used any other prescription medication (excluding hormonal contraception and hormone replacement therapy) within 14 days or five half-lives (whichever is longer) prior to Day 1 of the dosing period that the Investigator judges is likely to interfere with the trial or pose an additional risk in participating

25. Has used any over-the-counter medication (including multivitamin, herbal, or homeopathic preparations;) during the seven days or five half-lives of the medication (whichever is longer) prior to Day 1 of the dosing period, that the Investigator judges is likely to interfere with the trial or pose an additional risk in participating
26. Consumption of any herbal remedies or dietary supplements containing a herbal remedy in the 30 days before the planned Day 1 of the dosing period
27. Ingestion of any poppy seeds within the 24 hours prior to screening and admission
28. Known or suspected intolerance or hypersensitivity to the investigational products, any closely related compound, or any of the stated ingredients
29. History of significant allergic reaction (e.g. anaphylaxis, angioedema, but excluding untreated, asymptomatic, seasonal allergies) to any product (food, pharmaceutical, etc)
30. Donation of blood or blood products (excluding plasma) within 90 days prior to trial medication administration
31. Has a mental incapacity or language barriers precluding adequate understanding, co-operation, or compliance with the trial requirements
32. An inability to follow a standardised diet and meal schedule or inability to fast, as required during the trial
33. Inability to swallow up to eight tablets in short succession
34. Participants with veins on either arm that are unsuitable for intravenous puncture or cannulation (e.g. veins that are difficult to locate, or a tendency to rupture during puncture)
35. Prior screen failure (where the cause of the screen failure is not deemed to be temporary), randomisation, participation, or enrolment in this trial. Participants who initially failed due to temporary non-medically significant issues are eligible for re-screening once the cause has resolved
36. Participants who have received or are planning on receiving a COVID-19 vaccination four weeks before first dose administration, or within one week after trial completion
37. Any conditions which in the opinion of the Investigator would make the participant unsuitable for enrolment or could interfere with the participants' participation in or completion of the trial.

5.5 Participant restrictions

Participants will have to comply with the restrictions described in Table 11. Participants must also comply with the latest COVID-19 safety measures/ testing applicable at the site at that time, for entry into the unit and during in-house stays.

Table 11: Participant restrictions

Items participants must not consume or do	When participants must stop	When participants can re-start
Tobacco in any form (e.g. smoking or chewing) or other nicotine-containing products in any form (e.g. gum, patch, electronic cigarettes).	From six months prior to the planned first day of dosing.	After trial completion/last visit.
Meals/snacks/water.	Whenever participants are confined in the ward, only the drinks and meals provided by the trial personnel will be allowed.<br><br>Standard meals will be provided at the standard unit times as stated in the trial plan, and meals should be completed each time.<br><br>On each dosing day, participants will be required to fast from all food and drink except water a minimum of three hours before and four hours after dosing.<br><br>Water will be provided until one hour pre-dose and from one hour post-dose.	Standard meals will be given at regular intervals throughout the in-house stay.
Caffeine-containing or Xanthine-containing products.	48 hours before the planned first trial drug administration and each out-patient/ follow-up visit.	After trial completion/last visit.
Energy drinks or drinks containing taurine, glucuronolactone (e.g. Red Bull).	48 hours before the planned first trial drug administration and each out-patient/ follow-up visit.	After trial completion/last visit.
Alcohol.	48 hours before the planned first trial drug administration and each out-patient/ follow-up visit. On other days: less than 14 units a week and less than three units in one day is permitted.	After trial completion/last visit.
Strenuous physical activity.	48 hours before screening, admission and out-patient/follow-up visit.	After trial completion/last visit.
Activity.	Participants will be requested to remain in a semi-supine position for the first four hours after dosing each day, except to use the bathroom. Participants may then be ambulatory but should not engage in strenuous activities and should rest semi-supine for at least five minutes prior to any Vital Signs or at least	

Items participants must not consume or do	When participants must stop	When participants can re-start
	ten minutes prior to ECG measurement.	
Any prescription medication. For details, including exceptions see Section 5.4 Exclusion Criteria 21 and 23, and Section 6.3 Concomitant Medications.	14 days or five half-lives (whichever is longer) before the planned first trial drug administration.	After trial completion/last visit. If participants have a medical need to take any medication or have any medications prescribed to them by a doctor, they should follow the medical advice but inform the Investigator as soon as possible afterwards. Participants should be informed not to stop taking any medication that has been prescribed by their general practitioner or other doctor.
Any over-the-counter medication. For details, including exceptions, see Section 5.4 Exclusion Criterion 24.	Seven days or five half-lives (whichever is longer) before the planned first trial drug administration.	
Any herbal remedy or dietary supplement containing a herbal remedy.	30 days before the planned first trial drug administration.	After trial completion/last visit.
Not consume any other substances known to be potent inhibitors or inducers of the CYP3A4 system. This includes food or drink products containing cranberry, pomegranate, star fruit, grapefruit, pomelos, exotic citrus fruits or Seville oranges (including marmalade and juices made from these fruits).	Within 30 days or five half-lives (whichever is longer) before the planned first trial drug administration.	After trial completion/last visit.
Blood and plasma donation.	90 days before the planned first trial drug administration.	Three months after trial completion/last visit.
Excessive UV radiation exposure. Participants should use sun protective measures and limit exposure to natural sunlight (including occupational exposure to the sun or sunbathing) and avoid artificial sunlight (tanning beds or phototherapy). Ideally, outdoor activities should be scheduled outside the hours that ultraviolet radiation is most intense or should be performed in the shade. Participants should be advised to use sun protective measures (such as a hat, sunglasses, protective clothing, and sunscreen SPF ≥30).	Excessive sun exposure should be avoided from Admission (Day -1).	Participants can resume usual sun exposure 110 days (slightly less than 16 weeks) after the last dose of the IMP.

Items participants must not consume or do	When participants must stop	When participants can re-start
Contraception: Participants must follow the contraceptive requirements as stated in Section 5.3.	Start times for contraceptives vary according to method used - see applicable contraceptive method in Section 5.3.	110 days (slightly less than 16 weeks) after the last dose of IMP.
COVID-19 vaccination.	Four weeks before the planned first trial drug administration.	Seven days after trial completion/last visit.

5.6 Criteria for withdrawal

The Investigator or designee may withdraw a participant from the trial if the participant:

- is in violation of the protocol, which may jeopardise the trial results or represent a risk to the participant
- has an AE warranting withdrawal
- becomes pregnant
- meets individual rules criteria
- use of/need for a prohibited medication which in the opinion of the Sponsor or Investigator may jeopardise the trial results or represent a risk to the participant
- requests to be withdrawn from the trial (participant withdrawal of consent)
- is found to be considerably non-compliant with the protocol-required dosing visits
- in the Investigator's opinion, is unable to continue trial participation
- is withdrawn from the trial upon the request of Sponsor or the SRC, including if the Sponsor terminates the trial.

5.6.1 Handling of withdrawals

If a participant withdraws or is withdrawn from the trial, the Investigator will inform the Sponsor immediately. If there is a medical reason for withdrawal, the participant will remain under the supervision of the Investigator for protocol-specified safety follow up procedures.

Should any of the participants be withdrawn from the trial (by the Investigator and/or Sponsor) after being dosed, all the relevant assessments in relation to last dose should be completed as per protocol.

Should a participant withdraw themselves from the trial, every effort should be made to conduct a complete Early Termination (ET) visit at an appropriate time-point. The procedures required for the 'last visit' should be performed at this visit.

A participant who fails to return for final evaluations will be contacted by the site in an attempt to have the participant comply with the protocol, in accordance with the site SOPs.

When a participant withdraws from the trial, the primary reason for discontinuation must be recorded in the appropriate section of the Case Report Form (CRF).

6. TRIAL AND CONCOMITANT TREATMENTS

The trial treatments and concomitant therapies are categorised as described below and are shown in Figure 3 below.

An '**Investigational Medicinal Product (IMP)**' is defined as a pharmaceutical form of an active substance or placebo being tested or used as a reference in a clinical trial, including products already with a marketing authorisation but used or assembled (formulated or packaged) in a way different from the authorised form, or when used for an unauthorised indication, or when used to gain further information about the authorised form.

- The term '**test IMP**' is used to indicate only the experimental product under trial or development. Note: This term is more specific than 'IMP' which includes comparators and placebos.
- The term '**reference IMP**' is used to indicate any medicinal product being used as a comparator or reference substance (including placebo). Note: This term is more specific than 'IMP' which includes the experimental product and placebos.
- The '**non-IMP (NIMP)**' is defined as any medicinal product(s) intended for research and development trials, which does not fall within the definition of an IMP. NIMPs include:
    - the rescue medication (for ineffective treatment, anticipated ARs, or anticipated emergency situations): e.g. analgesic rescue medication or laxative in opioid studies, naltrexone
    - challenge agents, e.g. skin prick tests
    - medicinal products used to assess end-points in the clinical trial, e.g. any diagnostic agents used to assess the disease under trial
    - concomitant medicinal products systematically prescribed to the trial participants, e.g. cancer treatment in an opioid trial with cancer patients who all get the same cancer treatment according to the protocol
    - background treatment, standard care that all patients receive in addition to the IMP and for the same indication, e.g. standard chemotherapy in addition to a new oncological product to be tested.

Figure 3: Trial treatments and concomitant therapies

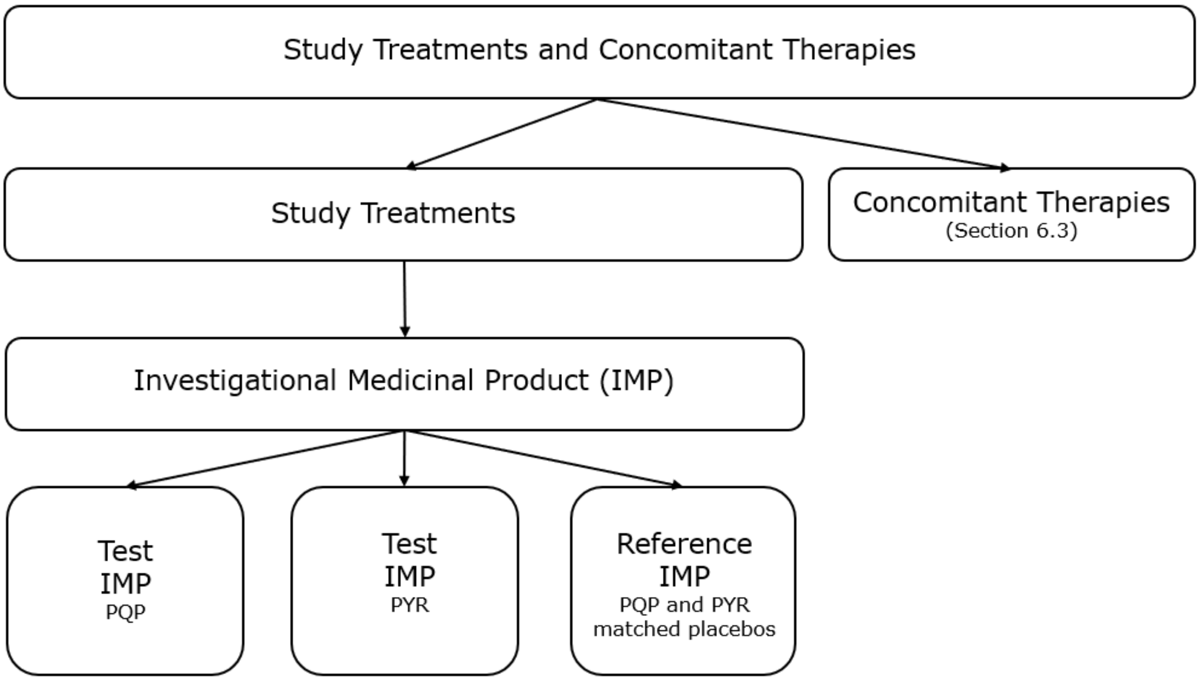

Abbreviations: IMP: Investigational medicinal product; PQP: piperaquine; PYR: pyronaridine.

6.1 Investigational Medicinal Products (IMPs)

The following IMPs will be used in this trial:

- piperaquine tetraphosphate – the test IMP
- pyronaridine tetraphosphate – the test IMP
- piperaquine matched placebo – the reference IMP
- pyronaridine matched placebo – the reference IMP.

The following IMP strengths will be used in this trial:

- PYR – 180 mg tablets
- PQP – 320 mg tablets
- PQP matched placebo tablets
- PYR matched placebo tablets.

Bulk IMPs will be supplied and QP certified by MMV or their contractor. Prior to being used on the clinical trial, the IMPs will be prepared by Richmond Pharmacology's clinical trials pharmacy. The prepared individual participant doses will be QP certified and dispensed by the site's pharmacy staff.

PQP is uncoated and bitter to taste, the effect may be persistent for some time after administration. This effect cannot be masked and may provide an indication of the treatment received. As the key safety and tolerability endpoints are objective assessments and parameters, it is anticipated that there will be no impact on the primary trial objective to determine the safety and tolerability of the registered doses of IMP.

6.1.1 Packaging and labelling of IMPs

The labelling of the trial drugs will be in compliance with Good Manufacturing Practice (GMP) specifications, as described in The Rules Governing Medicinal Products in the European Union, Volume 4, Annex 13, Investigational Medicinal Products, and any other or local applicable regulations.

Sample label(s) will be submitted to the UK health authorities according to the submission requirements.

6.1.2 Drug administration

PYR, PQP and matched placebos will be administered orally as detailed in Table 12. Doses of IMP will depend on the participant's body weight. The weight taken on Day -1 should be compared to the weight taken at screening to ensure there is no more than 5 % variation. More details will be provided in the SOM.

Table 12: IMP or placebo for each anticipated dose level

Treatment group	Weight (kg)	Treatment:	IMP/Placebo Dispensed:	Administration
1	50 - <65	*PYR 540 mg + PQP 960 mg	Three PYR tablets of 180 mg + three PQP tablets of 320 mg	Participants will fast from all food and drink (except for water) for a minimum of three hours prior to dosing.<br>Participants may drink water until one hour prior to dosing, and from one hour after dosing.<br>IMPs will be administered to each participant with approximately 240 mL of water.
	65 - <75	*PYR 720 mg + PQP 960 mg	Four PYR tablets of 180 mg + three PQP tablets of 320 mg	
	≥75	*PYR 720 mg + PQP 1280 mg	Four PYR tablets of 180 mg + four PQP tablets of 320 mg	
2	50 - <65	*PYR 540 mg + placebo for PQP	Three PYR tablets of 180 mg + three placebo PQP tablets	
	65 - <75	*PYR 720 mg + placebo for PQP	Four PYR tablets of 180 mg + three placebo PQP tablets	
	≥75	PYR 720 mg + placebo for PQP	Four PYR tablets of 180 mg + four placebo PQP tablets	
3	50 - <65	*placebo for PYR + PQP 960 mg	Three placebo PYR tablets + three PQP tablets of 320 mg	
	65 - <75	placebo for PYR + PQP 960 mg	Four placebo PYR tablets + three PQP tablets of 320 mg	
	≥75	*placebo for PYR + PQP 1280 mg	Four placebo PYR tablets + four PQP tablets of 320 mg	
4	50 - <65	placebo for PYR + placebo for PQP	Three placebo PYR tablets + three placebo PQP tablets	
	65 - <75	placebo for PYR + placebo for PQP	Four placebo PYR tablets + three placebo PQP tablets	
	≥75	placebo for PYR + placebo for PQP	Four placebo PYR tablets + four placebo PQP tablets	

Abbreviations: IMP: investigational medicinal product; mL: millilitres; PQP: piperaquine; PYR: pyronaridine.

*Efforts will be made to recruit participants in order to stratify randomisation according to weight, so that a minimum of two participants receive each of the seven treatment options, i.e. ≥ two participants will be administered with each of the following treatment options:

- PYR 540 mg + PQP 960 mg
- PYR 720 mg + PQP 960 mg
- PYR 720 mg + PQP 1280 mg
- PYR 540 mg + placebo for PQP
- PYR 720 mg + placebo for PQP
- placebo for PYR + PQP 960 mg
- placebo for PYR + PQP 1280 mg.

An RP will verify doses of PYR, PQP or matched placebo(s) and the details of dosing will be recorded in the CRF. The dosing will be verified by another member of the Investigator's staff. Detailed instructions for dose administration will be included in the SOM.

In the event that a participant vomits following IMP administration on a particular dosing day, that participant will not be re-dosed on that same day, however they will receive IMP on the subsequent days as planned (as per Table 5: Schedule of Assessments). The exact time of vomiting will be documented in the source document and eCRF for PK analysis purposes (i.e. to refine sub-populations depending on the time of vomiting).

6.1.3 Storage of IMPs

PYR, PQP and matched placebos will be stored in accordance with the labelling instructions as defined in the IMPD. The IMPs will be stored securely in a temperature-controlled pharmacy with authorised access only.

6.1.4 Drug accountability

The designated pharmacy staff at the clinical trial site will maintain accurate records of the receipt and the condition of all trial IMP including dates of receipt. In addition, accurate records will be kept by the pharmacy staff of when and how much trial IMP is dispensed and used by each participant in the trial. Any reason for departure from the protocol dispensing regimen must also be recorded.

IMP accountability records and inventory will be available for verification by the Sponsor or designee. At the completion of the trial, there will be a final reconciliation of all trial IMPs.

A trial IMP must not be used for any purpose other than the present trial. The destruction of remaining trial IMP will be arranged by the clinical trial site according to applicable regulations and only after receipt of written authorisation from the Sponsor.

6.2 Treatment allocation and blinding

6.2.1 Participant randomisation

All participants in this trial will be assigned to a treatment regimen according to a randomisation schedule generated by a statistician using PROC Plan. Details regarding the unique screening and participant number will be included in the SOM.

Eligible participants will be randomly assigned in a 2:1:1:1 ratio to one of four treatment arms on Day 1, at the point of dosing. Treatment arms are outlined below:

- Treatment 1 (N=16): PYR + PQP
- Treatment 2 (N=8) : PYR + placebo for PQP
- Treatment 3 (N=8) : placebo for PYR + PQP
- Treatment 4 (N=8) : placebo for PYR + placebo for PQP.

Sentinel dosing (N=10) will occur: four participants for treatment arm 1, and two participants from each of treatment arms 2, 3 and 4, will be dosed before the remaining participants of each treatment arm are dosed together (see Section 3.4.4).

6.2.2 Methods for ensuring blinding

The trial will take place in a double-blind fashion whereby participants and clinical trial site staff are blinded to the active or placebo trial drug assignment.

PQP is uncoated and bitter to taste, the effect may be persistent for some time after administration. This effect can not be masked and may provide an indication of the treatment received. As the key safety and tolerability endpoints are objective assessments and parameters, it is anticipated that there will be no impact on the primary trial objective to determine the safety and tolerability of the registered doses of IMP.

The pharmacy staff preparing the investigational products will not be blinded to trial drug assignment. During the trial, the individual randomisation codes will be kept in the site's clinical trials pharmacy, accessible to the pharmacy personnel only. Upon completion of the trial, after the database lock and after the blind is revealed, the randomisation list will be filed in the Trial Master File (TMF).

Sponsor staff involved in clinical decision-making (such as those involved in SRC decisions) will be blinded to trial drug assignment (see AR rules section above).

6.2.3 Methods for unblinding the trial

In the event of an emergency, an envelope for each participant containing his/her trial drug assignment will be available in the pharmacy at the clinical trial site. Unblinding should only be considered for the safety of the participant. If unblinding is deemed necessary by the Investigator, the Investigator or designee can unblind the participant’s treatment allocation using the envelope available from the pharmacy. The Investigator or designee must note the date, time and reason for unblinding and inform the sponsor of unblinding as soon as practicably possible.

If there is an unblinding, the randomisation schedule should be revised and this should be communicated to the RPL pharmacy team. Unblinding if required will be done in accordance with RPL SOP. SRC can decided whether to unblind a participant in either an emergency or non-emergency fashion.

6.3 Concomitant medications/permitted medications

The use of hormonal contraception and hormone replacement therapy is permitted. During the trial, other prescription or over-the-counter medications may be permitted at the discretion of the Investigator. The need for other medication may lead to participant’s withdrawal from the trial. In any case, the Investigator will inform the sponsor about the concurrent medication given.

Ibuprofen/NSAIDS should be used for rescue pain relief only, at the discretion of the PI. The dose and reason for ibuprofen/NSAID use in AEs other than as rescue medication must be recorded in the CRF and source record.

Details of all other prior and concomitant medications should be recorded by the Investigator on the CRF and source record.

6.4 COVID-19 vaccinations

Given the current COVID-19 pandemic, consideration has been made regarding participants who have had the vaccination or may be offered the vaccination during trial participation (see Section 5.4).

To optimise the data collected from this trial, the following rules will apply.

Participants may not be considered for participation if they:

- have received a COVID-19 vaccination within the four weeks prior to Day 1 of the trial
- plan to receive a vaccination from one week after trial completion/last visit.

7. TRIAL METHODOLOGY

7.1 Medical history

All clinically significant medical history (including any significant surgical procedures) must be recorded on the CRF for each participant. Each participant's full medical history will be obtained through direct questioning and the medical assessment at screening and will be updated at the day of admission (Day -1). If any clinically relevant observations or investigation results are detected prior to dosing, they will be recorded as medical history.

7.2 Eligibility check

The RP will perform a trial eligibility check for all trial participants during the screening period and on Day -1, as described in the Schedule of Assessments (Table 5 and Table 6). Further details will be provided in the SOM.

7.3 Prior and concomitant medication check

Each participant will have a prior and concomitant medication check as described in the Schedule of Assessments (Table 5 and Table 6) and in Section 0.

7.4 Beck depression inventory

Each participant will undergo a Beck Depression Inventory questionnaire [26], as described in the Schedule of Assessments (Table 5 and Table 6). A participant will be excluded from the trial if they have a score of 20 or more on the Beck Depression Inventory, and/or a response of 1, 2 or 3 for item 9 of this inventory (related to suicidal ideation) [26].

7.5 Meals

Standardised meals will be provided during the trial period according to the timings described in the Schedule of Assessments (Table 5 and Table 6).

On dosing days, participants will fast of all food and drink (except water) for at least three hours prior to IMP administration and for at least four hours post-dose.

Participants can drink water until one hour pre-dose and from one hour post-dose. Meals on the discharge day are optional.

7.6 Clinical laboratory assessments

Laboratory parameters to be measured are presented in Table 13.

Table 13: Laboratory parameters

Haematology	Biochemistry	Urinalysis
• Platelets	• Aspartate aminotransferase (AST)	• Leukocytes
• Hb	• Alanine aminotransferase (ALT)	• Nitrite
• Haematocrit	• Alkaline phosphatase (ALP)	• Urobilinogen
• White blood cells	• BUN	• Protein
• Neutrophils	• Gamma GT	• pH
• Eosinophils	• Total bilirubin	• Blood
• Basophils	• Direct bilirubin	• Specific gravity
• Lymphocytes	• Indirect bilirubin	• Ketones
• Monocytes	• Creatinine	• Bilirubin
• Red blood cells	• Urea	• Glucose
• Mean cell haemoglobin (MCH)	• Total Serum Proteins	• Urine microscopy**
• Mean corpuscular haemoglobin concentration (MCHC)	• Albumin	
• Mean corpuscular volume (MCV)	• Alpha1 Glycoprotein#	
• HbA1c***	• Sodium	
	• Potassium	
**Coagulation**	• Calcium	**Urine Screen for Drugs of Abuse**
• aPTT	• Corrected calcium	• Benzodiazepines
• PT	• Magnesium	• Opiates
• Fibrinogen	• Chloride	• Amphetamines
• INR	• Bicarbonate	• Methamphetamines
**Serology**	• Amylase	• Methadone
• Hepatitis B surface Antigen (HBsAg)	• Total cholesterol	• Cocaine
• Hepatitis B core Antibody (anti-HBC IgG + IgM, if IgG positive)	• Triglycerides	• Cannabinoids
• Hepatitis C Antibody (anti-HCV)	• Serum pregnancy test*	• Barbiturates
• HIV I and II Antibodies	• FSH*	• Phencyclidine
		• Tricyclic antidepressants

* Measured at the times presented in the Schedule of Assessments (Table 5 and Table 6). Serum pregnancy test for pre-menopausal women only; FSH for post-menopausal women only. ** Only if urinalysis is abnormal. *** Measured at screening only. # On admission day only.

7.6.1 Haematology, biochemistry and coagulation

Blood samples to determine biochemistry, haematology and coagulation parameters will be taken at the times given in the Schedule of Assessments (Table 5 and Table 6). The date and time of collection will be recorded on the appropriate CRF pages. Further details will be described in the SOM.

7.6.2 Serology

Serology will take place at screening as detailed in the Schedule of Assessments (Table 5 and Table 6). At the screening visit all participants will be tested for the parameters listed in Table 13. This is for the safety of the trial personnel and the result from the tests will not be entered into the trial database.

If a participant is found to be confirmed positive in any of these tests, they will be referred for further examination/treatment and will not be included in the trial, with the exception of a participant with a confirmed positive Anti-HBc IgG and negative Anti-HBc IgM and negative HBsAg, indicative of natural immunity due to a past infection without active chronic or acute infection.

The serology tests will be analysed in the same blood sample used for biochemistry.

7.6.3 Pregnancy testing

Serum β-hCG pregnancy tests will be performed for females of child-bearing potential, as per the Schedule of Assessments (Table 5 and Table 6); as well as any time a pregnancy is suspected. The results of the pregnancy test must be known before IMP administration. Participants who are pregnant will not be eligible for trial participation. Any woman with a positive pregnancy test will not receive any further IMP but will continue to be followed up for safety until the pregnancy outcome is known. See Section 8.5 for follow-up instructions.

7.6.4 Urinalysis

Urine samples for determination of urinalysis parameters will be taken at the times given in the Schedule of Assessments (Table 5 and Table 6). If deemed necessary, based on a clinically significant positive test, microscopic examination of urine will be performed.

7.6.5 Drugs of abuse

Urine will be tested for the drugs of abuse as described in the Schedule of Assessments (Table 5 and Table 6). If a participant fails the drug abuse screen, they will be excluded from the trial. A repeat drug screen can be performed where methodological reasons are believed to have led to a false positive. If participants are suspected to be positive due to medication e.g. flu/cold remedies, they may undergo a repeat drug screen.

7.6.6 FSH assessment

The postmenopausal status of women will be confirmed by:

1) a positive medical history of at least one year of amenorrhoea **and**
2) an increased blood FSH level (>30 IU/L).

FSH will be measured in postmenopausal female participants at the time of screening (Table 5).

7.7 Alcohol breath test

An alcohol breath test will be done using an alcometer as described in the Schedule of Assessments (Table 5 and Table 6). If a participant tests positive to the test they will be excluded from the trial.

7.8 Vital Signs (blood pressure, pulse rate, respiratory rate and tympanic temperature)

Vital signs will be measured at the time points as detailed in the Schedule of Assessments (Table 5 and Table 6). Blood pressure and pulse rate will be measured in supine position after the participant has rested comfortably for at least five minutes, using automated blood pressure monitors. Respiratory rate will be measured by manual counting for one minute. Temperature will be measured using tympanic thermometers. Standing vitals where described in the Schedule of Assessments (Table 5 and Table 6) will occur after standing for one minute.

7.9 Electrocardiographic (ECG) measurements

7.9.1 Recording of 12-lead ECGs

12-lead ECGs will be recorded at the time-points described in the Schedule of Assessments (Table 5 and Table 6) using a GE ECG recorder connected via a fixed network connection to the MUSE® Cardiology Information System (MUSE®). ECGs recorded during screening will be stored electronically on the MUSE® information system. Only ECG recorded electronically will be valid ECG for any purpose other than safety assessment. ECG printouts may be filed in the participant's CRF for medical safety reviews.

Each ECG recorder will be set up to the required technical specifications and will contain the information required to identify the records. Each ECG recording will be clearly identified (with participant ID, visit date, and the actual times of ECG recordings).

12-lead ECG recordings will be made after the participants have been resting in a supine position for at least ten minutes. The participants will avoid postural changes during the ECG recordings and clinical staff will ensure that participants are awake during the ECG recording.

At each time point, the ECG will be recorded in triplicate, to reduce variance and to improve the precision of measurement. The triplicates will be performed at approximately one-minute intervals. Each ECG recording (trace) will last ten seconds. Repeat ECG will be performed until at least three ten-second ECG records per scheduled time-point meet the quality criteria set out in the SOM and the

applicable SOP to enable the reading and analysing of at least five complexes per derivation.

7.9.2 Safety review of 12-lead ECGs

All recorded ECGs will be reviewed by an RP and the review will be documented in the CRF. If a participant shows an abnormal ECG, additional safety recordings (including the use of 5-lead or 12-lead Holter equipment) may be made and the abnormality be followed to resolution if required.

7.9.3 24-hour Holter ECG

Holter recording will be performed at screening as described in Table 5. Each electronic Holter ECG file will be downloaded onto the GE Gatemed Holter Analysis system.

7.9.4 Real Time ECG Telemetry

A 12-lead real time ECG will be recorded as described in the Schedule of Assessments (Table 5 and Table 6). ECG telemetry will be monitored by the investigator or qualified member of clinical staff. The system will be managed according to local working practices. The ECG telemetry reports will be archived with study documents.

7.9.5 Analysing and over-reading 12-lead ECG for the purpose of intensive cardiac assessments

Each electronic ECG will contain the ECG data as well as the result of the automated ECG analysis performed by the Marquette® 12SL™ ECG Analysis Program, a program resident in each of the ECG machines. All of the ECG and its associated automated interval measurements will subsequently be reviewed by qualified cardiologists in accordance with the ICH E14 Guidance for Industry document and ICH E14 Implementation Working Group Questions and Answers document, before any of the ECG is used for the thorough ECG analysis. The manual adjudication process applied in this trial is also referred to in the ICH guidance and relevant literature as 'manual over-read', 'computer-assisted' or 'semi-automated' ECG measurements. The following parameters on each ECG will be assessed by a cardiologist using the commercially available MUSE® in its latest version. The intention is to also measure sub-intervals:

- QT interval and sub intervals
- RR interval
- heart rate (HR)
- PR interval
- presence or absence of U-wave
- quantitative and qualitative ECG variations.

Manual on-screen over-reading using electronic callipers in MUSE® will be performed by a small and select group of cardiologists with extensive experience

with manual QT measurement (including on-screen measurement with electronic callipers). For all trial ECG the over-reading cardiologists will be blinded to time, date, treatment and any data that identify the participant. All ECGs of a given participant will be over-read by the same cardiologist (or cardiologists in case manual adjustments of the automated measurement are necessary).

7.10 Physical examination, height, weight and BMI

The physical examination performed at screening and at the post-trial visit will include an assessment of the following: general appearance, skin, eyes, ears, nose, neck, lymph nodes, throat, heart, lungs, abdomen, musculoskeletal system and extremities. The timings of the physical examinations are described in the Schedule of Assessments (Table 5 and Table 6).

Height will be measured in centimetres and weight in kilograms. Measurements should be taken with participants wearing light clothing and without shoes, using calibrated scales for all measurements. BMI will be calculated from the height and weight. Full details will be described in the SOM.

7.11 Pharmacokinetic assessments

7.11.1 PK blood samples

For the timing of individual samples refer to the Schedule of Assessments (Table 5 and Table 6). The date and time of collection will be recorded on the appropriate CRF.

Plasma samples to determine PQP (and N-oxidated metabolite of PQP, provided a future bioanalytical method is available) and blood samples to determine PYR will be analysed by Swiss BioQuant AG using a validated method. Full details of the analytical methods used will be described in a separate bioanalytical report. All sample handling procedures, including the time of each sample collection, the time of placement into frozen storage (at the end of the sample workup), and the date of transfer or shipment of the samples to the responsible analyst will be documented in detail in the SOM.

7.12 Volume of blood sampling

The maximum total blood volume collected from participants that take part in this trial will not exceed 470 mL overall. The NHS blood donation policy at the time of completion of this protocol (https://www.blood.co.uk/who-can-give-blood/) states that women can give 470 mL of blood every 16 weeks. Therefore, the 470 mL permitted in this trial is consistent with current clinical practice.

7.13 CYP (polymorphism) sampling

Blood samples for *CYP* polymorphism will be collected from all participants as indicated in the Schedule of Assessments (Table 5 and Table 6). The sample analysis will be optional and may be performed retrospectively, if decided by the Sponsor.

8. ADVERSE EVENTS

The collection, evaluation and reporting of AEs/ARs arising from this clinical trial will be performed in accordance with:

- detailed guidance on the collection, verification and presentation of AE/AR reports arising from clinical trials on medicinal products for human use ('CT-3') (2011/C 172/01)
- International Conference on Harmonization (ICH) harmonised tripartite guideline on clinical safety data management: 'Definitions and standards for expedited reporting' E2A
- ICH harmonised tripartite guideline on Development Safety Update Report: E2F
- ICH guideline E2F 'Note for guidance on Development Safety Update Reports (DSUR)'.

It is the Investigator's responsibility to document and report all AEs that occur in the clinical trial. The period of observation for collection of AEs extends from the signing of the ICF until the final visit. Additionally, spontaneously reported Serious Adverse Events (SAEs) will be collected until 30 days after the final trial visit. SAEs experienced after this 30-day period will only be reported if the Investigator suspects a causal relationship with the trial drug.

8.1 Definitions

An '**Adverse Event (AE)**' is any unfavourable and unintended sign (including an abnormal laboratory finding), symptom or disease temporally associated with the use of a medicinal product, whether considered related to it or not.

An AE may be:

- a new symptom or medical condition
- a new diagnosis
- an inter-current illness or an accident
- a worsening of a medical condition/disease(s) that existed before the start of the clinical trial
- the recurrence of a disease
- an increase in frequency or intensity of episodic diseases
- a change in a laboratory or other clinical test parameter
    - the criteria to determine whether an abnormal test result should be reported as an AE/AR are as follows. The abnormal test result:
        - is associated with accompanying symptoms, and/or
        - requires additional diagnostic testing or medical/surgical intervention
- an event that leads to a change in trial dosing outside of protocol-stipulated dose adjustments, or discontinuation from the trial, significant additional concomitant drug treatment, or other therapy, and/or

- considered an Adverse Event by the Investigator or Sponsor.

An AE does not necessarily include the following:

- an abnormal test that needs repeating, in the absence of any of the above conditions. Any abnormal test result that is determined to be an error does not require reporting as an Adverse Event
- surgical procedures themselves are not AEs; they are therapeutic measures for conditions that require surgery. The condition for which the surgery is required may be an AE/AR. Planned surgical measures permitted by the clinical trial protocol and the condition(s) leading to these measures are not AEs, if the condition leading to the measure was present before inclusion in the trial and has not worsened. In the latter case, the condition should be reported as medical history.

A '**Serious Adverse Event (SAE)**' is defined as any Adverse Event that fulfils any of the following criteria:

- it results in death
- it is life-threatening (the term 'life-threatening' in the definition of 'serious' refers to an event in which the participant was at immediate risk of death at the time of the event; it does not refer to an event which hypothetically might have caused death if it were more severe)
- it requires a hospitalisation* or prolongs existing hospitalisation
- it results in persistent or significant disability/incapacity
- it is a congenital abnormality/birth defect
- it is considered medically important (medical and scientific judgement should be exercised in deciding whether other AE/ARs are to be considered serious, such as important medical events that may not be immediately life-threatening or result in death or hospitalisation* but may jeopardise the participant or may require intervention to prevent one of the other outcomes listed in the definition above. Examples of such events are intensive treatment in an emergency room or at home for allergic bronchospasm; blood dyscrasias; convulsions that do not result in hospitalisation; development of drug dependency or drug abuse).

*'Hospitalisation' qualifying for an SAEs does not include the following: under 24 hours; admission unrelated to an Adverse Event e.g. for labour and delivery, cosmetic surgery, social or administrative for temporary placement (e.g. admission due to lack of transport or support at home); admissions for diagnosis or therapy of a pre-existing condition that has not increased in severity or frequency; protocol-specified admission; pre-planned admission; admission to a rehabilitation or nursing facility; hospice; presentation to emergency departments or other urgent care centres; admissions to hospital in-patient facilities for logistical reasons only that did not result in any therapeutic intervention (e.g. awaiting consultation with and decision by senior medical staff or specialists, or for an investigation that is not immediately available); admissions to hospital in-patient facilities for investigation alone and where no significant abnormality was identified and/or no therapeutic intervention was necessary; same-day outpatient surgery.

An '**Adverse Reaction (AR)**' is a response to a medicinal product that is noxious and unintended, which occurs at any dose (in pre-approval clinical experience) or

at doses normally used in man for prophylaxis, diagnosis, or therapy of disease or for modification of physiological function (in post-approval clinical experience). The term 'reaction' means that a causal relationship between a medicinal product and an Adverse Event is at least a reasonable possibility. This means that there are facts (evidence) or arguments to suggest a causal relationship.

A '**Serious Adverse Reaction (SAR)**' is any AR that fulfils the criteria of seriousness, as defined above.

A '**Suspected Unexpected Serious Adverse Reaction (SUSAR)**' is a Serious AR that is unexpected. The 'expectedness' of a serious AR is assessed in the light of the Reference Safety Information (RSI).

8.2 Classification

8.2.1 Assessment of severity

The Investigator will assess the severity of AE/ARs using a categorical grading (mild, moderate or severe) with grading of individual AE/ARs based on global clinical assessment undertaken by an appropriately delegated Research Physician.

Changes in the severity of an AE/AR should be documented to allow an assessment of the AE/AR duration at each level of severity. AE/AR characterised as intermittent require documentation of the start and end of each incidence.

The table on the following page guides categorical grading of AE/ARs.

Table 14: Categorical grading of AE/ARs

Grade	Global clinical assessment & application of grading to a diagnosis
Mild	Overall clinical condition is asymptomatic, or symptomatic with all the following characteristics:<br>• no or minimal interference with usual social and/or functional activities<br>• no impact, or minimal impact (e.g. of short duration or a return to normal status without intervention), upon usual self-care activities<br>• deviation from reference range for the population and from the participant's clinically determined physiological baseline has no clinical impact and does not signal potential safety concern or unanticipated clinical risk<br>• the condition is reversible to baseline within an anticipated timeframe<br>• intervention or treatment is either not indicated or indicated only to increase participant's sense of wellbeing and comfort, but not due to safety concerns or risk.
Moderate	Overall clinical condition is asymptomatic or is symptomatic with no or minimal impact on usual self-care activities and one or more of the following characteristics:<br>• greater than minimal interference with usual social & functional activities<br>• deviation from reference range for the population and from the participant's baseline has clinical impact, but there is no acute safety concern or unanticipated risk. However, should the condition persist and/or occur more frequently, it could signal a potential safety concern or unanticipated risk and should therefore be monitored<br>• reversibility of the condition to baseline condition takes longer than desirable or anticipated, impacting on the participant's wellbeing beyond the mere severity of the condition<br>• further medical assessment, intervention or treatment indicated to increase participant's sense of wellbeing and comfort and to accelerate recovery and/or to prevent worsening.
Severe	Overall clinical condition is asymptomatic or is symptomatic with one or more of the following characteristics:<br>• significant impact on usual self-care activities<br>• inability to perform usual social & functional activities<br>• the deviation from reference range for the population and from the participant's baseline has significant clinical impact, it is an acute safety concern or unanticipated risk and requires close and continuous monitoring<br>• the reversibility of the condition to baseline condition takes significantly longer than desirable or anticipated, with significant impact on the participant's wellbeing<br>• intervention or treatment essential to treat the acute manifestations and to prevent worsening<br>• life-threatening or actually leads to death.

8.2.2 Assessment of causality

AEs will be evaluated for whether they meet the criteria for an adverse reaction (AR). An AR is a response to IMP which is noxious and unintended, and which occurs at any dose. The term "reaction" means that a causal relationship between IMP and an adverse event is at least a reasonable possibility. This

means that there are facts (evidence) or arguments to suggest a causal relationship.

- The Investigator will use clinical judgment to determine the relationship.
- Alternative causes, such as underlying disease(s), concomitant therapy, and other risk factors, as well as the temporal relationship of the event to IMP administration must be assessed.
- The Investigator will also consult the Investigator's Brochure (IB)/SmPC in his/her assessment.
- For each AE/SAE, the Investigator must document in the medical notes that he/she has reviewed the AE/SAE and has provided an assessment of causality.
- There may be situations in which an SAE has occurred and the Investigator has minimal information to include in the initial report to MMV. However, it is important that the Investigator attempts to make an assessment of causality before the initial transmission of the SAE data to the sponsor or designee.
- This is due to the fact that the causality assessment is one of the criteria used when determining regulatory reporting requirements.
- The Investigator may change his/her opinion of causality in light of follow-up information and send an SAE follow-up report with the updated causality assessment.
- The Investigator should also comment on the source document whether an AE is not related to the study treatment but is related to study participation (e.g. study procedures, wash-out periods etc.). AEs where a causal relationship between study drug and the AE is at least a reasonable possibility, are referred to as ARs.

The causality assessment of an AE to the IMP will be rated as follows by the Investigator:

'**Related**': An AE for which it can be reasonably explained that the study drug caused the AE by available facts (evidence) or arguments. For example, the occurrence of the AE cannot be explained by other causative factors, but can be explained by pharmacological effect of the study drug such as:

- temporal relationship to IMP exposure
- event is known to be associated with the IMP drug class
- event improved on discontinuation or dose reduction of IMP
- event reoccurred on re-challenge of IMP
- biological plausibility
- other (must be specified).

'**Not Related**': An AE which cannot be reasonably explained by available facts (evidence) or arguments that the study drug caused the AE. For example, the occurrence of the AE can be explained by other causative factors, such as:

- event attributed to concomitant medication (provide details of the concomitant medication)
- event attributed to the concurrent disease(s)/condition(s) (provide details of the disease/condition)

- event attributed to a non-investigational medicinal product (NIMP) (specify the NIMP)
- no reasonably temporal relationship associated with IMP administration
- event is expected in the study indication and/or target population
- negative de-challenge and/or negative re-challenge
- other (must be specified).

The Investigator should also comment on the source document whether an AE is not related to the study treatment but is related to study participation (e.g. study procedures, wash-out periods etc.).

The relationship to the study medication will be classified according to the categories described in Table 14.

8.2.3 Practical application of severity grading and causality assessment in relation to AR rules

Step 1: Confirm diagnosis of the AE/AR (exact term will be decided using the MedDRA), once the root cause of the clinical manifestations has been determined (rather than diagnosing individual signs, symptoms, assessments, and measurements).

Step 2: Distinguish between serious and non-serious AE/ARs using the standard definitions of what constitutes a 'serious' AE/AR (see Section 8.1).

Step 3: Assess whether there is a reasonable possibility of a relationship with the IMP.

Step 4: If there is a reasonable possibility that it is related to the IMP, apply the correct AR rules (see Section 4.4):

- '**serious AR**': The classification as 'serious' (see Sections 8.1 and 8.2) overrides the application of the categorical (mild/moderate/severe) grading system. The rules for serious AR must be applied. Categorical grading (moderate or severe) should also be independently recorded to add additional information.
- '**non-serious AR**':
  - some individual clinical manifestations, signs, symptoms, assessments, or measurements (such as QT prolongation, anaemia, thrombocytopenia, liver enzyme abnormalities) have pre-defined special AR rules, which should be applied if they occur
  - for all other non-serious ARs use the global clinical assessment and categorical grading method outlined above to assign the severity grade (Section 8.2). Consider:
    - the clinical significance (including presence or absence of clinical symptoms)
    - participant populations' specific references ranges and degree of deviations thereof
    - the degree of deviations from baseline
    - timing, duration, and reversibility

- the nature and intensity of treatment required and the responses to the treatment; in addition, the potential to worsen or to become defined as serious
- apply the general AR rules according to the assigned categorical severity grade.

8.2.4 Expectedness (reference safety information)

No serious adverse reactions (SARs) are considered expected by the Sponsor for the purpose of expedited reporting of suspected, unexpected serious adverse reactions (SUSARs).

8.2.5 Adverse Events of Special Interest (AESI)

An 'AESI' is an Adverse Event of scientific or medical concern that is specific to the Sponsor or the particular product or program, for which ongoing monitoring and rapid communication by the Investigator to the Sponsor may be appropriate. It may require further investigation in order to be characterised and understood. It could be serious or non-serious and could include events that might be potential precursors or prodromes for more serious medical conditions in susceptible individuals. If an AESI is serious, reporting procedures for SAE/SUSAR will be used. For non-serious AESI, reporting procedures will be described in the SOM.

The AESIs for this protocol include, but are not limited to, the following:

- hepatic:
  - Moderate: any ALT or AST >3 x and <5 x ULN
  - Severe: any ALT or AST >3 x and <5 x ULN with symptoms/ ALT or AST >5 x ULN with or without symptoms*
  - Serious: ALT or AST >3 x ULN together with an increase in bilirubin to >1.5 x ULN
- cardiac:
  - uncorrected QT interval >500 msecs, using consistent, technically valid triplicate ECG
  - second- or third-degree atrioventricular (AV) block
  - bundle branch block
  - any arrhythmia
- haematological:
  - Hb drop of >25 % from baseline on Day -1 (only for values below the laboratory reference range), or to an absolute value of <100 g/L
  - platelet count drop of >25 % from baseline on Day -1 (only for values outside the laboratory reference range) or absolute value of <80 x 10^9/L.
  - clinically significant drop from baseline in neutrophil count**

**symptoms include fatigue, nausea, vomiting, right upper quadrant pain or tenderness, fever, rash and/or eosinophilia (eosinophil percent or count above the ULN).*

***baseline refers to all samples taken prior to dosing on Day 1 (e.g. at screening, on Day -1).*

8.3 Recording of adverse events and follow-up

All (serious and non-serious) Adverse Events detected by the Investigator or spontaneously notified by the participant at each visit/examination must be reported on the particular section of the CRF.

The following information should be reported for each Adverse Event, whether it can be attributed to trial drug or not:

- description of Adverse Event
- date of onset/date of resolution
- characteristics of the event (seriousness, intensity)
- actions taken (required treatment or dose adjustments must be reported in the CRF)
- outcome
- relationship with trial drug (causality assessment) and/or trial participation.

All Adverse Events must be documented and followed up until the event is either resolved or a satisfactory explanation is found, or the Investigator considers it medically justifiable to terminate the follow-up. The reason(s) will be recorded in the CRF when the AE follow-up is terminated.

Symptoms and signs that are being recorded as other methods specified in the protocol should not be additionally recorded as AEs, unless it leads to the participant's withdrawal from dosing, or it is one symptom of another diagnosis.

8.4 Reporting of Serious Adverse Events

Detailed reporting procedures will be outlined in the pharmacovigilance plan.

If an SAE occurs, the Investigators will take appropriate action immediately and will strive to identify the cause(s) of the events.

All SAEs/SUSARs will be notified by the Investigator to the MMV Medical Monitor, the MMV Medical Director and to Prime Vigilance within 24 hours of awareness by email to:

Name: Dr Annick Janin MD (MMV Medical Monitor)

[redacted]

Name: Oliver Abschütz (Prime Vigilance)

[redacted]

Name: Stephan Chalon (MMV Medical Director)

[redacted]

using the 'Serious Adverse Event Report Form for Clinical Trials'. The pharmacovigilance provider will notify the Sponsor within one working day after receipt of the SAE form.

The SAE follow-up observation period, for the concerned participants, will be jointly decided by the Investigator, Sponsor and/or SRC.

The initial report will be followed up by a full written report within three working days or five calendar days, whichever is sooner, unless no further information is available. In that case, the follow-up report will be provided as soon as new information becomes available. Further follow-up reports will be provided as and when new information becomes available. Photocopies of relevant CRF pages, such as demography, medical history, concomitant medications, as well as test results, consultant report(s), a summary of the outcome of the reaction and the Investigator's opinion of IMPs relationship to the SAE/SUSAR will accompany the SAE form if and when available.

The Sponsor will also perform an evaluation of all SAEs.

SUSARs will be notified to the Competent Authority by MMV and to the relevant REC(s) by MMV within seven days (for fatal and life-threatening SUSARs) or 15 days (all other SUSARs).

Annual safety reporting to the national Competent Authority and the REC will be in agreement with ICH guideline E2F 'Note for guidance on Development Safety Update Reports (DSUR)'.

8.5 Pregnancy

Pregnancy in itself is not regarded as an AE, unless there is a suspicion that the IMP may have interfered with the effectiveness of a contraceptive medication.

However, complications of pregnancy and abnormal outcomes of pregnancy are AEs, and many may meet criteria for an SAE. Complications of pregnancy and abnormal outcomes of pregnancy, such as ectopic pregnancy, spontaneous abortion, intrauterine foetal demise, neonatal death, or congenital anomaly, would meet the criteria of an SAE and therefore should be reported as an SAE. Elective abortions without complications should not be handled as an AE.

If a participant becomes or is found to be pregnant while being treated or exposed to trial drug, the Investigator must submit the 'Pregnancy Reporting and Outcome/Breast Feeding Form' to Prime Vigilance and MMV via the same method as SAE reporting.

The participant should be followed until the outcome of the pregnancy is known (spontaneous miscarriage, elective termination, normal birth or congenital abnormality), When the outcome of the pregnancy becomes known, the form should be updated and returned to Prime Vigilance and MMV. If additional follow-up of the participant is required, the Investigator will be requested to provide the information.

8.5.1 Pregnancy in female partners of male participants

If a male participant's female partner becomes or is found to be pregnant while the participant was being treated or exposed to trial drug, the Investigator must submit the 'Pregnancy Reporting and Outcome/Breast Feeding Form' to RPL and MMV via the same method as SAE reporting.

Male participants may continue in the trial if an accidental pregnancy of their female partner occurs despite adequate contraception.

The male participant's partner should be followed until the outcome of the pregnancy is known (spontaneous miscarriage, elective termination, normal birth or congenital abnormality), even if the male participant discontinues the trial drug or discontinues from the trial. When the outcome of the pregnancy becomes known, the form should be updated and returned to RPL and MMV. If additional follow-up of the female partner is required, the Investigator will be requested to provide the information.

9. QUALITY ASSURANCE AND QUALITY CONTROL

9.1 Quality Assurance and Quality Control

Regulatory agencies may carry out a Regulatory Inspection of this trial. Such audits/inspections can occur at any time during or after completion of the trial. If an audit or inspection occurs, the PI and RPL agree to allow the auditor/inspector direct access to all relevant documents and to allocate their time and the time of their staff to the auditor/inspector to discuss any findings or relevant issues.

Quality Control (QC) procedures at the RPL will be implemented to ensure data recorded into the CRFs are accurate. QC checks will be carried out on an ongoing basis and according to the relevant SOPs. Records of QC checks will be documented and available for review.

9.2 Monitoring

All aspects of the trial will be carefully monitored by the Sponsor, or designee, for compliance with applicable government regulations with respect to Good Clinical Practice (GCP) and current Standard Operating Procedures.

The monitoring of this trial will be performed by the Sponsor's monitor(s) or a designee in accordance with the principles of GCP as laid out in the International Council for Harmonisation of Technical Requirements for Pharmaceuticals for Human Use (ICH) Good Clinical Practice Guideline E6(R2) (2016).

The clinical monitor, as a representative of the Sponsor, has an obligation to follow the trial closely. In doing so, the monitor will visit the Investigator and site periodically and will maintain frequent telephone and email contact. The monitor will maintain current personal knowledge of the trial through observation, review of trial records and source documentation, and discussion of the conduct of the trial with the Investigator and staff. Further details will be described in the SOM.

10. STATISTICAL ANALYSIS

10.1 Statistical analysis plan

A Statistical Analysis Plan (SAP) containing detailed statistical methodology will be written and signed off before the database hard lock. The plan may be updated to reflect adaptive features of the trial as appropriate.

10.2 Analysis sets

The analysis of data will be based on different analysis sets according to the purpose of analysis. Participant eligibility for each analysis set will be finalised before the database hard lock. A participant who withdraws prior to the last planned observation in a trial period will be included in the analyses up to the time of discontinuation.

10.2.1 Safety set

The safety set will consist of all randomised participants who received at least one dose of the IMP. The safety set will be used for the safety analyses.

10.2.2 PK set

The PK set will consist of those participants in the safety set who have sufficient blood samples taken for at least one of the PK variables to be calculated. The PK set will be used for the PK analyses.

10.2.3 ECG set

The ECG set will consist of those participants in the safety set that have at least one valid pre-dose ECG assessment and one valid post-dose assessment. An ECG

assessment will be considered valid if it is based on at least two evaluable replicates with measurable QT_c and RR.

The analysis set for intensive cardiac assessment will be based on the intersection of the PK set and the ECG set. In addition, participants on placebo will be included with plasma concentrations of PYR and PQP set to zero. Individual QT_c/concentration pairs will be excluded from this set if the time of ECG and the time of blood sampling are too far apart, based on time window deviations defined in DHP. Details will be defined in the SAP.

10.3 Statistical analysis of safety

Individual participant demographics (age, gender and race) and body measurement data (height, weight and BMI) at screening will be listed. These demographic characteristics and body measurements will be summarised by treatment group and overall, using the safety analysis set. Other baseline characteristics will be listed only.

AE data will be listed and summarised using descriptive statistics: the number (and percentage) of participants who experienced any AEs and the number of AE episodes will be summarised for each treatment group. All AEs will be summarised and listed by using the System Organ Class (SOC) and Preferred Term (PT) assigned to the event using the MedDRA. Furthermore, these events will be summarised by their maximum intensity. The number of participants who experienced drug-related AEs will also be summarised. Any SAEs and/or AEs that led to trial withdrawal will be summarised and listed.

Vital signs data (SBP, DBP, HR, temperature) will be listed and summarised, along with changes from baseline, using descriptive statistics (mean, median, Standard Deviation, minimum, maximum). Out-of-reference-range values will be flagged as high or low and as being clinically relevant or not. The number of participants presenting out-of-range and clinically relevant values will be summarised.

All safety clinical laboratory data will be listed. Laboratory test results will also be compared to laboratory reference ranges. Those values outside of the applicable range will be flagged as high or low, and as clinically relevant or not. The number of participants that present out-of-range, and clinically relevant values, will be summarised. The quantitative laboratory data and changes from baseline will be summarised using descriptive statistics (mean, median, Standard Deviation, minimum, maximum). Change from baseline values at each assessment will be calculated as the assessment value minus the baseline value. The qualitative urinalysis data will be listed only.

ECG analyses will be performed on two sets of ECGs: all ECGs prior to adjudication and selected triplicates from each time-point after adjudication.

All un-adjudicated ECG data (PR, QRS, QT, QT_cB, QT_cF and HR) and overall ECG evaluation will be listed. ECG data and changes from baseline will be summarised using descriptive statistics (mean, median, Standard Deviation, minimum, maximum).

Furthermore, categorical analysis of QT_cF data will be presented as follows:

- absolute QT_cF interval prolongation
    - QT_cF interval >450 ms

  - QT_cF interval >480 ms
  - QT_cF interval >500 ms
- change from baseline in QT_cF interval
  - QT_cF interval increases from baseline >30 ms
  - QT_cF interval increases from baseline >60 ms.

The mean change from baseline value of QT_cF will be plotted by treatment group and by time-point. Out-of-reference-range values will be flagged as clinically relevant or not. The number of participants presenting out-of-range and clinically relevant values will be summarised.

A similar categorical analysis will be presented as follows:

- increase of PR from baseline >25% resulting in PR > 200 ms
- increase of QRS from baseline >25% resulting in QRS > 120 ms
- decrease of HR from baseline >25% resulting in HR < 50 bpm
- increase of HR from baseline >25% resulting in HR > 100 bpm.

10.4 ECG analysis

Analysis of drug related QT/QT_c interval changes relative to plasma PK concentrations will be conducted. The principles of this analysis follow the statistical methods described by Garnett et al., 2018 [20]. This analysis will be performed on the analysis set for intensive cardiac assessment.

In addition, a per timepoint analysis will be performed. While this analysis is based on minimal assumptions on the nature of the data, it has limited power to show the absence of a prolonging effect. On the other hand, the concentration-QT_c analysis has been shown to have this power, but it is based on more restrictive assumptions. Concentration/ QT_c modelling may be performed after completion of the Clinical Study Report and will be delivered in a separate report.

10.4.1 Baseline

For all quantitative parameters, baseline will be the average over the values obtained at the three pre-dose timepoints of Day 1.

10.4.2 Heart rate correction

QT_cF will be used for ECG analyses unless there is an effect of one of the drugs on HR exceeding 10 bpm. Evidence of the absence of such an effect will be considered given, if either the absolute mean time matched difference to placebo is bolow 10 bpm for all timepoints and for both monotherapies and the combination therapy or a concentration-effect model similar to the one described below excludes an effect exceeding 10 bpm at relecant concentrations. If an effect of the therapy on HR cannot be excluded following the criteria above, QT_cI will be determined based on data extracted from the 24h Holter data and the primary correction method will be selected based on the mean squared slope criteria [28] using the Day -1 12-lead ECG data. Details will be given in the SAP.

10.4.3 Per timepoint analysis

For all quantitative ECG parameters, descriptive statistics will be given for the change from baseline. In addition, for QT_c, a linear model with the two treatments (PYR and PQP) and their interaction as factors and baseline as covariate will be fitted for each timepoint; the difference between each of the active treatments and placebo will be estimated based on this model; and two-sided 90% confidence intervals (CI) will be given.

10.4.4 Hysteresis

Hysteresis between the effect of each of the monotherapies and its concentration will be investigated based on the results of the per timepoint analysis and the mean concentrations under these therapies. Details will be given in the SAP.

10.4.5 Concentration-QT_c analysis

This analysis will be based on the change of QT_c from baseline and will use baseline QT_c (centred on the mean across participants) and the concentrations of PYR and PQP as covariates. Concentrations of relevant metabolites may also be included. Treatment with levels Placebo, PYR, PQP and combination (referred to as "treatment intercept" in the sequel) and time will be used as discrete fixed effects. A series of linear models including one or more of the concentrations will be fitted and the most appropriate one will be selected based on the Akaike Information Criterion (AIC) and the size of the fixed treatment effect. A significant treatment effect (based on an F-test) will be considered an indication for model misfit. For the best fitting linear model, predictions of the effect on QT_c will be made. For these predictions, the T_{max} of every moiety included in the model will be determined and the mean concentration of all moietis involved at these timepoints will be used. Details of the model selection procedure and the concentrations to be included in the best fitting model will be given in the SAP.

Linearity of the concentration-ΔQTc relationship will be explored based on quantile plots as described in Garnett et al (2018) [20]. In addition, a significant treatment intercept can be interpreted as sign of either hysteresis or nonlinearity. If the best fitting linear model has a significant treatment effect, nonlinear e-max models will also be considered, and predictions will be made based on the best fitting model. In this case, hysteresis between QT_c and the concentrations of the relevant analytes will be investigated graphically.

Absence of an effect of concern of the IMP on QT_c will be concluded if the predictions based on the best-fitting model at the concentrations described above exclude an effect exceeding 10 ms, i.e. the upper limit of the two-sided confidence interval for these predictions is below 10 ms.

10.4.6 Assay sensitivity

In a study analysing the effect of a combination of OZ439 and PQP, Darpö et al 2015 [27] found an effect of PQP concentration on QTc of 0.0475 ms/(ng/mL) with a 90 % confidence interval of (0.038, 0.057). If the effect of PQP in the primary

linear model of this analysis is similar to this value, assay sensitivity will be deemed shown.

10.5 Pharmacokinetics

10.5.1 Evaluation of pharmacokinetic parameters

Non-compartmental analysis will be used for estimation of PK parameters.

At least the following PK parameters will be calculated for PYR and PQP (and N-oxidated metabolite of PQP, provided a future bioanalytical method is available):

Table 15: PK parameters

C_{max}	Maximal plasma concentration
t_{max}	Time to reach maximum plasma concentration
$t_{1/2}$	Terminal Elimination Half-life
AUC_{0-inf}	Area under the plasma concentration-time curve from time zero extrapolated to infinite time
AUC_{0-t}	Area under the plasma concentration curve from time zero up to the last quantifiable concentration
$\%AUC_{extrap}$	Percentage of AUC that is due to extrapolation from l_{ast} to infinity
CL/F	Apparent total plasma clearance
V_z/F	Apparent volume of distribution during the terminal phase

The individual plasma concentration data, and the actual time for PYR and PQP administration and blood sampling will be used in the derivation of the PK parameters. If there is any doubt as to the actual time that a sample was taken, then the scheduled time will be used.

AUC_{0-t} and AUC_{0-inf} will be calculated using the linear/log trapezoidal method, applying the linear trapezoidal rule up to C_{max} and the log trapezoidal rule for the remainder of the curve. Samples below the Limit of Quantification (LOQ) prior to the first quantifiable concentration will be set to zero. Samples with concentrations below LOQ after the first quantifiable concentration will be set to 'missing' and omitted from the analysis. Other pharmacokinetic parameters will be calculated according to standard equations. Details will be provided in the SAP.

10.5.2 Statistical analysis on PK parameters

Plasma concentrations will be listed and summarised by time point, including:

- the number of participants (N)
- the number of samples (n)
- the number of samples <LLOQ (nLLOQ)
- arithmetic mean

- Standard Deviation (SD)
- Coefficient of Variation (CV)
- geometric mean
- median
- minimum
- maximum.

The PK parameters will be listed for each participant and summarised for each treatment group using descriptive statistics, including:

- N
- arithmetic mean
- SD
- CV
- geometric mean
- median
- minimum
- maximum.

10.6 Handling of missing and incomplete data

Unrecorded values will be treated as missing. The appropriateness of the method(s) for handling missing data may be reassessed at the data review prior to database lock.

10.7 Determination of sample size

This is an exploratory trial and the sample-size is not based on formal statistical considerations. The numbers assigned to each treatment group are considered adequate to describe the tolerability, safety and pharmacokinetics.

Assuming that an event occurs in 10 % of the cases, the probability to see at least one case in 16 subjects on combination treatment is 81 %. If the event occurs in 20 % of the cases, this probability increases to 97 %.

11. DATA MANAGEMENT

The data management department of RPL will perform data management. The Data Handling Protocol (DHP) will outline the data management process in detail.

The RPL data management department will:

- develop and maintain the DHP
- set and validate the clinical trial database
- programme validation checks
- enter data into the clinical trial database

- review data for accuracy, completeness, and consistency between the CRF and the database
- verify adherence to the clinical pharmacology trial protocol and the DHP.

Clinical data queries will be generated and resolved according to the DHP. RPL clinical staff, trial RP/PI will assist to resolve clinical data queries.

After all clinical data queries are resolved, final error rate is confirmed, and QC checks are acceptable, the database will be locked.

Standard Statistical Analysis System (SAS®) datasets will be generated from the final trial database ready for analyses.

RPL will perform medical coding. AEs, diagnoses from medical history and procedures from surgical history will be classified according to MedDRA. Concomitant medication will be coded using WHODRUG.

SAEs in the clinical database will be reconciled with the safety database.

Final raw SAS® datasets will be transferred to the statistician and the Sponsor (as applicable).

11.1 Case Report Forms

The Sponsor and the Investigator will sign a source data agreement to define what constitutes source data for all types of data captured.

Case Report Forms will be used to record source data in the trial. Data should be recorded legibly onto the CRFs in black ballpoint pen. Correction fluid or covering labels must not be used.

The monitor will check data at the monitoring visits to the trial site. The PI will ensure that the data in the CRFs are accurate, complete, and legible.

The data manager will ensure that data from the completed CRFs are entered into RPL's clinical trial database and validated. Screening failures (participants who signed consent to take part in the trial but were not randomised) and admission data for reserves will not be entered into the clinical trial database. Any missing, impossible (inconsistent with human life), or inconsistent recordings in the CRFs will be queried to the PI and be documented for each individual participant before clean file status is declared.

12. SPONSOR'S AND INVESTIGATOR'S RESPONSIBILITIES

12.1 Sponsor's responsibilities

12.1.1 GCP compliance

MMV and any third party to whom aspects of the trial management or monitoring have been delegated will undertake their roles for this trial in compliance with all applicable regulations and ICH GCP Guidelines.

Representatives of MMV will conduct visits to Investigator sites in order to inspect trial data, participants' medical records, and CRFs in accordance with current ICH Good Clinical Practice Guideline E6 (R2) (2016) and the respective local and national government regulations and guidelines. Additionally, auditors or competent authorities may review records and data.

12.1.2 Regulatory approval

MMV (or delegate) will ensure that local competent authority requirements are met before the start of the trial.

12.1.3 Indemnity/liability and insurance

MMV will adhere to the recommendations of the Association of British Pharmaceutical Industry (ABPI) Guidelines. The Investigator will receive a copy of the indemnity document before trial initiation.

MMV will ensure that suitable insurance cover is in place prior to the start of the trial. RPL will receive an insurance certificate and a statement of insurance.

12.1.4 Protocol management

MMV and/or RPL will prepare all protocols and amendments. If it becomes necessary to issue a protocol amendment during the trial, MMV will notify the Investigator and collect a documented Investigator agreement to the amendment.

12.1.5 End of trial notification

RPL on behalf of MMV will submit an end of trial notification to the MHRA within 90 days of the end of the trial in accordance with EU Directive 2001/20/EC. The PI will be responsible for submitting these to the REC within 90 days of the end of the trial.

For the purposes of this notification, the end of the trial will be defined as database lock.

12.1.6 Posting or submission of summary of clinical trial report to competent authorities of member states concerned and RECs

MMV or its delegate will post result-related information on this clinical trial in accordance with the UK Statutory Instrument within one year of the end of the complete trial, to the competent authority of the Member State concerned, as required by the regulatory requirement and to comply with the community guideline on Good Clinical Practice. RPL on behalf of MMV will send a short confirmatory email to CT.Submission@mhra.gov.uk once the result-related information has been uploaded to the public register and provide a link. In addition RPL on behalf of MMV will submit a summary of the clinical trial report to the concerned REC.

12.2 Investigator's responsibilities

12.2.1 GCP compliance

The Investigator must undertake to perform the trial in accordance with ICH GCP Guidelines, and the applicable MHRA regulatory requirements.

It is the Investigator's responsibility to ensure that adequate time and appropriate resources are available at the trial site prior to commitment to participate in this trial. The Investigator should also be able to estimate or to demonstrate a potential for recruiting the required number of suitable participants within the agreed recruitment period.

The Investigator will maintain a record of appropriately qualified persons to whom the Investigator has delegated significant trial-related tasks. Before the trial starts, MMV (or designee) will receive an up-to-date copy of the *curriculum vitaes* for the Investigator, sub-Investigator(s), and essential trial staff.

The PI will demonstrate agreement with the final clinical trial by dated signature, in compliance with UK Statutory Instrument and ICH E3.

12.2.2 Protocol adherence and Investigator agreement

The PI and delegates must adhere to the CSP as detailed in this document. The PI will be responsible for including only those participants who have met CSP eligibility criteria. The PI must sign an Investigator agreement to confirm acceptance and willingness for themselves and delegates to comply with the CSP.

12.2.3 Documentation and retention of records

After completion of the trial, the PI will keep all documents and data relating to the trial in a secure file and/or electronically, in a secure and orderly manner. The data will be available for inspection by MMV or their representatives. Essential documents must be retained for 25 years after the final marketing approval in an ICH region or until at least 25 years have elapsed since the formal discontinuation of clinical development of PYR and PQP. The PI or delegate must contact MMV before destroying any trial-related documentation and it is the responsibility of MMV to inform the investigative site of when these documents can be destroyed. In addition, all participant records and other source documentation will be kept for a longer period if required by the applicable regulatory requirements.

12.3 Ethical considerations

This protocol complies with the principles of the World Medical Assembly (Helsinki 1964) and subsequent amendments.

12.3.1 Informed consent

The informed consent is a process by which a participant voluntarily confirms his/her willingness to participate in a clinical trial. It is the responsibility of the PI or delegate to obtain written informed consent from participants. All consent documentation must be in accordance with applicable regulations and the ICH

Good Clinical Practice Guideline E6 (R2) (2016). Each participant will be requested to sign the ICF after they have received and read the written participant information and received an explanation of what the trial involves, including but not limited to: the objectives, potential benefits and risk, inconveniences, and the participant's rights and responsibilities. Signed ICFs must remain on file and must be available for verification by trial monitors at any time. Another signed original of the ICF must be given to the participant or the participant's legally authorised representative. The PI or delegate will provide the Sponsor with a copy of the REC approved consent forms, and a copy of the REC written approval, prior to the start of the trial.

12.3.2 Research Ethics Committee (REC) approval

It is the responsibility of the PI to submit this CSP, the informed consent document (approved by MMV), relevant supporting information, and all types of participant recruitment information to the REC for review, and all must be approved prior to the start of participant screening. In addition, advertisements must be approved by the REC prior to use at the site. Prior to implementing changes in the trial, MMV and the REC must also approve any substantial amendments to the CSP and corresponding updates to informed consent documents. For non-substantial protocol amendments (that do not require REC approval) and subsequent updates of the ICF, all changes will be made in agreement with MMV and RPL.

12.4 Confidentiality

For the purposes of this Section 12.4, 'Applicable Data Protection Law' shall mean (a) the Data Protection Act 2018; (b), the UK GDPR (as defined in Section 3(10) of the Data Protection Act 2018) and (c) the General Data Protection Regulation ((EU) 2016/679) as applicable, and any applicable legislation introduced in the UK.

Data collected during this trial may be used to support the development, registration, or marketing of medicinal products. MMV will control all data collected during the trial and will abide by the Applicable Data Protection Law. For the purpose of the Applicable Data Protection Law, MMV will be the data controller. To the extent that RPL processes personal data on behalf of MMV, in relation to such data RPL shall only act in accordance with the terms of this protocol and MMV's reasonable written instructions and RPL shall take appropriate technical and organisational measures against the unauthorised or unlawful processing of such personal data.

After participants have consented to take part in the trial, MMV and/or its representatives will review their medical records and the data collected during the trial. These records and data may, in addition, be reviewed by the following: independent auditors who validate the data on behalf of MMV, national or local regulatory authorities, and the REC which gave its approval for this trial to proceed.

Although participants will be known by a unique number, their date of birth will also be collected by RPL and be used to assist MMV to verify the accuracy of the data, for example, that the results of trial assessments are assigned to the correct participant. The results of this trial containing the unique number, date of birth, and relevant medical information including ethnicity may be recorded and transferred to and used in other countries throughout the world. If personal data

are transferred outside of the UK, RPL will ensure applicable data transfer mechanisms are in place to ensure the data receives essentially equivalent protections as are applicable in the UK. The purpose of any such transfer would be to support regulatory submissions made by MMV in such countries. The parties agree to comply with the relevant provisions of the Applicable Data Protection Law and any directions issued by the UK Information Commissioner's Office in its processing of such personal data. All nominative information in the participant's medical record will be kept strictly confidential. Nominative information shall mean the name, the address and all other personally identifiable information associated with a participant's name. MMV access to participant's data shall be performed in such a way that no participant could be identified by such data.

If there are any contradictions in terms of confidentiality requirements, the requirements of Applicable Data Protection Law will prevail.

To the extent that RPL processes personal data on behalf of MMV, the terms of Appendix 1 shall also apply.

12.5 Publication policy

If the Sponsor and RPL agree that it will be desirable to publish the results of this trial; both parties will liaise in good faith to publish the results. RPL agree to obtain the Sponsor's prior written approval of such publications.

13. REFERENCES

1. Geneva: World Health Organization. World malaria report 2020: 20 years of global progress and challenges. https://www.who.int/publications/i/item/9789240015791. Published 20 November 2020. Accessed 8 October 2021.
2. Chang C, Lin-Hua T, Jantanavivat C. Studies on a new antimalarial compound: Pyronaridine. Transactions of The Royal Society of Tropical Medicine and Hygiene. 1992;86, 1: 7-10. https://doi.org/10.1016/0035-9203(92)90414-8. Accessed 12 October 2021.
3. Zongo I, Milligan P, Compaore Y, Some A, Greenwood B, Tarning J, Rosenthal P, Sutherland C, Nosten F, Ouedraogo J. Randomized noninferiority trial of dihydroartemisinin-piperaquine compared with sulfadoxine-pyrimethamine plus amodiaquine for seasonal malaria chemoprevention in Burkina Faso. Antimicrobial Agents and Chemotherapy. 2015;59(8):4387-96. Accessed 25 November 2021.
4. European Medicines Agency. Eurartesim. https://www.ema.europa.eu/en/medicines/human/EPAR/eurartesim#product-information-Section. Published 2021. Accessed 8 October 2021.
5. Medicines for Malaria Venture. Pyramax® (Artesunate- pyronaridine) for the treatment of acute uncomplicated malaria in adults and children. https://www.mmv.org/access/products-projects/pyramax-pyronaridine-artesunate-treatment-acute-uncomplicated-malaria. Published 2019. Accessed 7 October 2021.
6. Bailly C. Pyronaridine: an update on its pharmacological activities and mechanisms of action. Biopolymers. 2020;112, 4. https://doi.org/10.1002/bip.23398. Accessed 7 October 2021.
7. DrugBank online. Piperaquine. https://go.drugbank.com/drugs/DB13941. Published 2021. Accessed 7 October 2021.
8. DrugBank online. Pyronaridine. https://go.drugbank.com/drugs/DB12975. Published 2021. Accessed 7 October 2021.
9. Myint H, Ashley E, Day N, Nosten F, White N. Efficacy and safety of dihydroartemisinin-piperaquine. Transactions of The Royal Society of Tropical Medicine and Hygiene. 2007;101(9): 858-66. https://pubmed.ncbi.nlm.nih.gov/17659311/. Accessed 7 October 2021.
10. Ratcliff A, Siswantoro H, Kenangalem E, Maristela R, Wuwung R, Laihad F, Ebsworth E, Anstey N, Tijtra E, Price R. Two fixed-dose artemisinin combinations for drug-resistant falciparum and vivax malaria in Papua, Indonesia: an open-label randomised comparison. Lancet. 2007;369(9563): 757-65. https://pubmed.ncbi.nlm.nih.gov/17336652/. Accessed 8 October 2021.

11. Internal MMV Material.
12. Karunajeewa H, Lim C, Hung T, Ilett K, Denis M, Socheat D, Davis T. Safety evaluation of fixed combination piperaquine plus dihydroartemisinin (Artekin) in Cambodian children and adults with malaria. British Journal of Clinical Pharmacology. 2007;57(1): 93-9. Accessed 8 October 2021.
13. Mytton O, Ashley E, Peto L, Price R, La Y, Hae R, Singhasivanon P, White N, Nosten F. Electrocardiographic safety evaluation of dihydroartemisinin piperaquine in the treatment of uncomplicated falciparum malaria. American Journal of Tropical Medicine and Hygiene. 2007; 77(3): 447-50. https://pubmed.ncbi.nlm.nih.gov/17827358/. Accessed 8 October 2021.
14. Wu W, Lu C, Liang Y, Zhang H, Deng C, Wang Q, Xu Q, Tan B, Zhou C, Song J. Electrocardiographic effect of artemisinin-piperaquine, dihydroartemisinin-piperaquine, and artemether-lumefantrine treatment in falciparum malaria patients. The Journal of the Brazilian Society of Tropical Medicine. 2021;54:e05362020. https://pubmed.ncbi.nlm.nih.gov/33605379/. Accessed 8 October 2021.
15. Credible Meds. Clinical Factors Associated with Prolonged QT_c and/or TdP. Crediblemeds.org. https://www.crediblemeds.org/research-scientists. Published 2013. Accessed 12 October 2021.
16. Funck-Brentano C, Ouologuem N, Duparc S, Felices M, Sirima S, Sagara I, Soulama I, Ouedraogo J, Beavogui A, Borghini-Fuhrer I, Khan Y, Djimdé A, Voiriot P. Evaluation of the effects on the QT-interval of 4 artemisinin-based combination therapies with a correction-free and heart rate-free method. Scientific Reports. 2019;9(1):883. https://pubmed.ncbi.nlm.nih.gov/30696921/. Accessed 8 November 2021.
17. Stein C, Fritsch C, Robinson C, Sbruzzi G, Plentz R. Effects of electrical stimulation in spastic muscles after stroke: systematic review and meta-analysis of randomised controlled trials. Stroke. 2015; 46(8):2197-205. https://pubmed.ncbi.nlm.nih.gov/26173724/. Accessed 12 October 2021.
18. Unpublished internal MMV Summary Safety Slideset Material.
19. Pyronaridine Investigator's Brochure, V2, Section 6.3.2.
20. Garnett C, Bonate P, Dang Q, Ferber G, Huang D, Liu J, Mehortra D, Riley P, Sager P, Tornoe C, Wang Y. Scientific white paper on concentration-QT_c modelling. Journal of Pharmacokinetics and Pharmacodynamics. 2018;45, 383-397. https://link.springer.com/article/10.1007%2Fs10928-017-9558-5. Accessed on 15 October 2021.
21. Unpublished internal MMV data; CSR SPC001-03.
22. Morris C, Lopez-Lazaro L, Jung D, Methaneethorn J, Duparc S, Borghini-Fuhrer I, Pokorny R, Shin C, Fleckenstein L. Drug-Drug Interaction Analysis of Pyronaridine-artesunate and Ritonavir in

Healthy Participants. American Journal of Tropical Medicine and Hygiene. 2012;86: 489- 495. Accessed 25 November 2021.

23. Morris C, Pokorny R, Lopez-Lazaro L, Miller R, Arbe-Barnes S, Duparc S, Borghini-Fuhrer I, Shin J, Fleckenstein L. Pharmacokinetic Interaction between Pyronaridine-Artesunate and Metoprolol. Antimicrobial Agents and Chemotherapy. 2014;58: 5900-5908. Accessed 25 November 2021.
24. Morris C, Dueker S, Lohstroh P, Wang LQ, Fang X, Jung D, Lopez-Lazaro L, Baker M, Duparc S, Borghini-Fuhrer I, Pokorny R, Shin J, Fleckenstein L. Mass balance and metabolism of the antimalarial pyronaridine in healthy participants. European Journal of Drug Metabolism and Pharmacokinetics. 2015;40: 75-86. Accessed 25 November 2021.
25. Sim I, Davis T, Ilett K. Effects of a high-fat meal on the relative oral bioavailability of piperaquine. Antimicrobial Agents and Chemotherapy. 2005;49(6): 2407-2411. https://europepmc.org/article/PMC/1140540. Accessed 10 November 2021.
26. Beck A, Ward C, Mendelson M, Mock J, Erbaugh J. An inventory for measuring depression. Archives of General Psychiatry. 1961;4:561-71. doi: 10.1001/archpsyc.1961.01710120031004. Accessed 26 Nov 2021.
27. Darpö B, Ferber G, Siegl P, Laurijssens B, Macintyre F, Toovey S, Duparc S. Evaluation of the QT effect of a combination of piperaquine and a novel anti-malarial drug candidate OZ439, for the treatment of uncomplicated malaria. British Journal of Clinical Pharmacology. 2015;80(4):706-15. Accessed 29 November 2021.
28. Tornøe C, Grnett C, Wang Y, Florian J, Li M, Gobburu J. Creation of a knowledge management system for QT analyses. The Journal of Clinical Pharmacology. 2011;51(7):1035–1042. Accessed 29 November 2021.

14. APPENDIX 1

Where RPL processes personal data on behalf of MMV, RPL shall act as a data processor and the Sponsor shall be a data controller. To the extent RPL is the data processor on behalf of MMV, RPL shall:

1.1 process the personal data only in accordance with the documented instructions of MMV

1.2 implement appropriate technical and organisational measures to protect the personal data against unauthorised or unlawful processing and against accidental loss, destruction, damage, alteration or disclosure. These measures shall be appropriate to the harm and risk which might result from any unauthorised or unlawful processing, accidental loss, destruction or damage to the personal data and having regard to the nature of the personal data which is to be protected and shall include inter alia as appropriate:

1.2.1 the pseudonymisation and encryption of personal data

1.2.2 the ability to ensure the ongoing confidentiality, integrity, availability and resilience of systems and services processing personal data

1.2.3 the ability to restore the availability and access to personal data in a timely manner in the event of a physical or technical incident

1.2.4 a process for regular testing, assessing and evaluating the effectiveness of technical and organisation measures for ensuring the security of processing.

In order to enable RPL to implement appropriate technical and organisational measures, MMV shall provide to RPL any information reasonably required by RPL to enable it to assess the appropriateness of such measures.

1.3 only employ or appoint personnel to process the personal data who have given binding undertakings of confidentiality or are under a statutory obligation of confidentiality

1.4 remain entitled to appoint third party sub-processors. Where RPL appoints a third-party sub-processor, it shall:

1.4.1 ensure that the third party is subject to, and contractually bound by, at least the same obligations as RPL

1.4.2 provide to MMV copies of any documentation to demonstrate compliance with the obligations under this section

1.4.3 remain fully liable to MMV for all acts and omissions of the third party

1.5 notify MMV without undue delay after becoming aware that it has suffered a data breach

1.6 at MMV’s cost, permit MMV (subject to reasonable and appropriate confidentiality undertakings), to inspect and audit RPL’s data processing activities to enable MMV to verify and/or procure that RPL is in full compliance with its obligations under the protocol

1.7 taking into account the nature of the processing, assist MMV by appropriate technical and organisational measures, insofar as this is possible, with the fulfilment of MMV's obligation to respond to requests from data participants in connection to this protocol exercising their rights under Applicable Data Protection Law

1.8 unless applicable law requires otherwise, upon termination of the protocol:

1.8.1 at the option of MMV, comply or procure the compliance with the following:

(a) return to MMV all personal data and any other information provided by MMV to RPL; and/or

(b) permanently delete all personal data provided by MMV to RPL; and

1.8.2 cease to process the personal data

Notwithstanding the foregoing, RPL shall be entitled to retain personal data to the extent that it is required to do so pursuant to the law of the United Kingdom

1.9 where the laws of the country where RPL is established require RPL to transfer the personal data to a third country or an international organisation, inform MMV as soon as reasonably possible of that legal requirement unless that law prohibits such communication on important grounds of public interest

1.10 RPL shall only transfer personal data outside the United Kingdom to a country or territory that is subject to a UK or European Commission adequacy decision, where appropriate safeguards are in place, and on the condition that the level of protection offered to the data subject in the third country are essentially equivalent to those offered by the Applicable Data Protection Law.
