# Supplementary material for: Randomized, placebo‐controlled, double‐blind phase I trial of co‐administered pyronaridine and piperaquine in healthy adults of sub‐Saharan origin
Source: Clin Transl Sci. 2024 Apr 9;17(4):e13738. doi: 10.1111/cts.13738 (PMC11004265; doi:10.1111/cts.13738)
Supplement: Supplementary file 2 — Figure S1. [file CTS-17-e13738-s002.pdf]

Supplementary Material

Randomized, Placebo-controlled, Double-blind Phase 1 Trial of Co-administered Pyronaridine and Piperaquine in Healthy Adults of Sub-Saharan Origin

*Andrea Kuemmerle[1], Denis Gossen[2], Annick Janin[3], Andrew Stokes[4], Nada Abla[1], Maja Szramowska[5], Ulrike Lorch[4], Myriam El Gaaloul[1], Isabelle Borghini-Fuhrer[1], Stephan Chalon[1]**

[1]Medicines for Malaria Venture, Geneva, Switzerland; [2]Mangareva SRL, Kraainem, Belgium; [3]AKJ Consulting, Divonne, France; [4]Richmond Pharmacology Ltd, London, United Kingdom; [5]PharmaKinetic Ltd., Quorn, United Kingdom.

CONTENTS

Table S1 Participant baseline demographics. 3

Figure S1 Frequency of treatment-emergent adverse events of any cause following administration of pyronaridine (PYR), piperaquine (PQP) or coadministration of PYR+PQP. Values are the percentage of participants experiencing the adverse event.. 4

Figure S2 Drug exposure to pyronaridine based on (a) AUC_{0-t} and (b) C_{max} for participants with or without an adverse event of special interest (AESI) of hypertransaminasemia................................... 5

Figure S3 Relationship between maximum values of (a) alanine aminotransferase (ALT) or (b) aspartate aminotransferase (AST) and total bilirubin for participants with hypertransaminasemia 6

Narratives S1 Safety narratives for adverse events of special interest. 7

Table S2 Time course of lymphocyte values for individual participants with values below the lower limit of normal. 9

Table S3 Time course of eosinophil % values for individual participants with values outside the limits of normal.. 10

Figure S4 Changes in blood pressure. 11

Table S4 Post-baseline categorical data for Fridericia-corrected QT interval (QTcF) change from baseline >30 msec and absolute values >450 msec. 13

Table S5. Fold changes comparing pyronaridine (PYR) exposure following PYR+placebo (n=8) with PYR exposure following PYR+piperaquine (PQP) coadministration. 16

Table S6. Fold changes comparing piperaquine (PQP) exposure following PQP+placebo (n=8) with PQP exposure following pyronaridine (PYR)+PQP coadministration.. 17

Table S1 Participant baseline demographics.

Parameter	PYR+PQP dose groups (mg)				PYR+placebo dose groups (mg)			PQP+placebo dose groups (mg)			Placebo
	All doses (N=15)	540/960 (n=5)	720/960 (n=5)	720/1280 (n=5)	All doses (N=8)	540 (n=3)	720 (n=5)	All doses (N=8)	960 (n=5)	1280 (n=3)	(N=6)
Sex, n (%)											
Male	7 (46.7)	0	3 (60.0)	4 (80.0)	4 (50.0)	0	4 (80.0)	3 (37.5)	1 (20.0)	2 (66.7)	2 (33.3)
Female	8 (53.3)	5 (100.0)	2 (40.0)	1 (20.0)	4 (50.0)	3 (100.0)	1 (20.0)	5 (62.5)	4 (80.0)	1 (33.3)	4 (66.7)
Women of child bearing potential[a]	8 (100)	5 (100)	2 (100)	1 (100)	4 (100)	3 (100)	1 (100)	5 (100)	4 (100)	1 (100)	4 (100)
Mean age, years (SD)	27.0 (6.24)	22.2 (1.64)	30.2 (6.65)	28.6 (6.69)	30.5 (5.93)	27.7 (4.04)	32.2 (6.61)	27.9 (5.87)	28.0 (5.96)	27.7 (7.02)	26.0 (6.03)
Mean height, cm (SD)	169.5 (10.03)	159.0 (4.64)	173.6 (10.69)	175.8 (2.86)	170.5 (6.07)	165.7 (4.04)	173.4 (5.32)	168.3 (12.44)	163.6 (12.01)	176.0 (10.44)	168.7 (5.82)
Mean weight, kg (SD)	70.30 (10.58)	58.58 (6.03)	71.26 (2.56)	81.06 (5.59)	70.40 (12.15)	59.80 (6.39)	76.76 (10.16)	69.78 (14.33)	60.82 (7.62)	84.70 (8.23)	72.47 (8.87)

PYR, pyronaridine; PQP, piperaquine; SD, standard deviation.

[a] Denominator is number of women.

Figure S1 Frequency of treatment-emergent adverse events of any cause following administration of pyronaridine (PYR), piperaquine (PQP) or coadministration of PYR+PQP. Values are the percentage of participants experiencing the adverse event.

	PYR+PQP All doses (N=15)	PYR+PQP 540/960 mg (n=5)	PYR+PQP 720/960 mg (n=5)	PYR+PQP 720/1280 mg (n=5)	PYR All doses (N=8)	PYR 540 mg (n=3)	PYR 720 mg (n=5)	PQP All doses (N=8)	PQP 960 mg (n=5)	PQP 1280 mg (n=3)	Placebo (n=6)
Any adverse event	66.7	80.0	80.0	40.0	87.5	100.0	80.0	50.0	40.0	66.7	83.3
Hypertransaminasemia	20.0	0	60.0	0	25.0	33.3	20.0	0	0	0	0
Fatigue	13.3	20.0	20.0	0	12.5	33.3	0	0	0	0	16.7
Headache	13.3	20.0	20.0	0	12.5	0	20.0	12.5	0	33.3	16.7
Thrombocytopenia	6.7	0	20.0	0	0	0	0	0	0	0	0
Dizziness postural	6.7	0	0	20.0	0	0	0	0	0	0	0
Change in bowel habit	6.7	0	20.0	0	0	0	0	0	0	0	0
Diarrhea	6.7	0	20.0	0	12.5	0	20.0	0	0	0	0
Nausea	6.7	20.0	0	0	12.5	33.3	0	25.0	20.0	33.3	0
Vomiting	6.7	20.0	0	0	12.5	33.3	0	0	0	0	0
Pruritis	6.7	20.0	0	0	0	0	0	0	0	0	16.7
Skin hyperpigmentation	6.7	0	0	20.0	0	0	0	0	0	0	0
Epitaxis	6.7	20.0	0	0	0	0	0	0	0	0	0
Palpitations	0	0	0	0	12.5	33.3	0	0	0	0	0
Presyncope	0	0	0	0	0	0	0	0	0	0	16.7
Visual impairment	0	0	0	0	12.5	33.3	0	0	0	0	0
Constipation	0	0	0	0	12.5	33.3	0	0	0	0	0
Frequent bowel movements	0	0	0	0	12.5	0	20.0	0	0	0	0
Gastroesophageal reflux disease	0	0	0	0	0	0	0	12.5	20.0	0	0
COVID-19	0	0	0	0	12.5	33.3	0	0	0	0	0
Nasopharyngitis	0	0	0	0	0	0	0	0	0	0	16.7
Pharyngitis	0	0	0	0	0	0	0	0	0	0	16.7
Sinusitis	0	0	0	0	0	0	0	12.5	0	33.3	0
Systemic viral infection	0	0	0	0	12.5	20.0	0	0	0	0	0
Upper RTI	0	0	0	0	0	0	0	25.0	20.0	33.3	0
Musculoskeletal chest pain	0	0	0	0	12.5	0	20.0	0	0	0	0
Orthostatic hypertension	0	0	0	0	0	0	0	12.5	20.0	0	0

COVID-19, coronavirus disease of 2019; PYR, pyronaridine; PQP, piperaquine; RTI, respiratory tract infection.

Figure S2 Drug exposure to pyronaridine based on (a) $AUC_{0\text{-}t}$ and (b) C_{max} for participants with or without an adverse event of special interest (AESI) of hypertransaminasemia

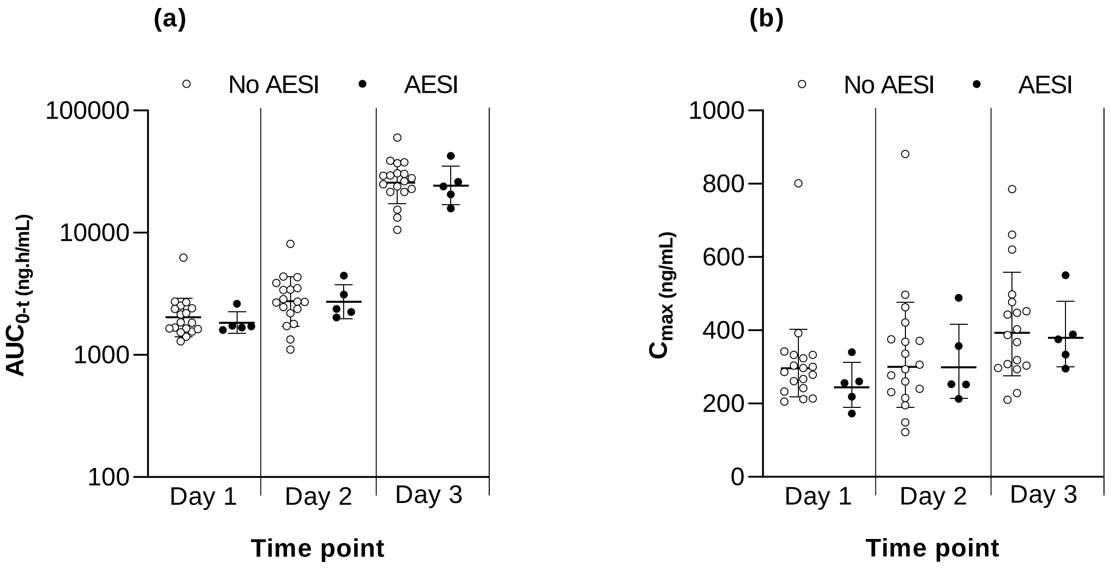

Figure S3 Relationship between maximum values of (a) alanine aminotransferase (ALT) or (b) aspartate aminotransferase (AST) and total bilirubin for participants with hypertransaminasemia

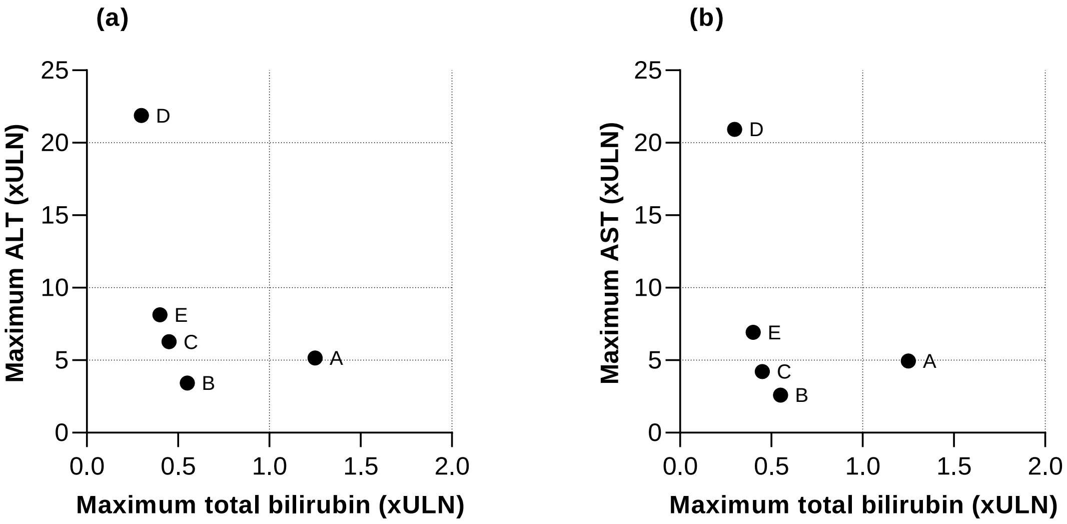

Participant identifiers (A to E) are indicated adjacent to the relevant data point.

Narratives S1 Safety narratives for adverse events of special interest.

Abbreviations

AESI, adverse event of special interest; ALP, alkaline phosphatase; ALT, alanine aminotransferase; AST, aspartate aminotransferase; GGT, gamma-glutamyl transferase; LFTs, Liver function tests; PYR, pyronaridine; PQP, piperaquine; ULN, upper limit of normal.

Narrative A

33 years of age/male; PYR 720 mg.

Asymptomatic elevated LFTs (hypertransaminasemia), severe, drug related, resolved.

Severely deranged ALT (>5xULN) and moderately deranged AST (>4xULN) and GGT (>3xULN). The transaminases were first elevated on D3, (ALT 63 IU/L, AST 59 IU/L). By D4 they fulfilled the AESI criteria with moderately elevated ALT (>3xULN) and AST (>4xULN). Levels peaked on D5, severely elevated ALT (>5xULN) and moderately elevated AST (>4xULN). From D6 onwards the LFTs began to recover. By D8, the ALT was down to 2xULN and the AST was only mildly raised at 52 IU/L. By D15 both transaminase levels were back to normal. There were no clinically significant bilirubin rises throughout the period of elevated LFTs up to Day 30. The participant remained asymptomatic throughout and no concomitant medications were required. They did not experience any relevant symptoms such as fatigue, nausea, vomiting, right upper quadrant pain, fever or rash. There were no clinically significant changes in vital signs, physical examination, ECG/telemetry or urinalysis. Of note, the participant was positive for anti-HBc IgG/IgM but negative for anti-HBc IgM at screening. Given that remaining hepatitis serology was negative, this is likely in keeping with past, resolved infection. Given the known safety profile of PYR in previous Phase 1 studies, and the temporal correlation to dosing, this was deemed to be related to drug administration and graded severe as per protocol.

Narrative B

34 years of age/male; PYR+PQP 720/960 mg.

Asymptomatic elevated LFTs (hypertransaminasemia), moderate, drug related, resolved.

The ALT was first raised on D3 at 148 IU/L, nearly 3xULN. ALT and AST peaked on D4 fulfilling AESI criteria as the ALT was 171 IU/L (>3xULN). The AST was >2xULN at 96 IU/L. On the same day, the participant's bilirubin was normal at 4 µmol/L (ULN 20 µmol/L), ALP at 107 IU/L (ULN 129 IU/L) and GGT was high at 114 IU/L (ULN 71). From D5 onwards the LFTs began to recover. By D8 the ALT was only mildly raised (71 IU/L), AST was within normal limits and GGT close to normal 78 (ULN 71 IU/L). All LFTs were within normal range on D15. Total bilirubin and ALP remained within normal range throughout the study. The participant remained asymptomatic throughout and no concomitant medications were required. They did not experience any relevant symptoms such as fatigue, nausea, vomiting, RUQ pain, fever, rash or an associated eosinophilia. There were no clinically significant changes in vital signs, physical examination, ECG/telemetry or urinalysis. Of note, Participant 10007 was positive for anti-HBc IgG/IgM but negative for anti-HBc IgM at screening. Given that remaining hepatitis serology was negative, this is likely in keeping with past, resolved infection. Given the known safety profile of PYR in previous Phase 1 studies, and the temporal correlation to dosing, this was deemed to be drug related and graded moderate as per protocol.

Narrative C

20 years of age/female; PYR+PQP 720/960 mg.

Asymptomatic elevated LFTs (hypertransaminasemia), severe, not drug related, resolving.

The temporal pattern of LFT elevations in this study participant was different from the other volunteers who also showed an ALT/AST elevation signal. AST first elevated D15, peaking on D25 at >3xULN (116 IU/L). ALT was first elevated on D15, again peaked D25 at >5xULN (182 IU/L). Thus, fulfilling AESI criteria. A recent Epstein Barr Virus (EBV) infection was documented on subsequent liver safety screening tests. Hepatitis C, Hepatitis B, Hepatitis A, and Hepatitis E serologies were negative. LFTS were returning to normal on unscheduled bloods taken on D49, ALT 65 IU/L (ULN 35 IU/L) and AST 45 IU/L (ULN 31 IU/L). Bilirubin, ALP and GGT remained within normal range throughout the study. The elevation of transaminases was associated with a lymphocytosis starting on Day 22. Lymphocytes 3.86 x 10^9/L (ULN 3.65) returning to normal on Day 28. Given the temporal relationship to dosing and documented acute EBV infection, this was deemed not drug related and graded as severe per protocol.

Narrative D
30 years of age/female; PYR 540 mg.
Asymptomatic elevated LFTs (hypertransaminasemia), severe, drug related, resolving.

Asymptomatic elevation of transaminases starting at D3 (AST and ALT >5xULN). Peak values D3 were ALT 766 IU/L (21.9xULN) and AST 649 IU/L (20.9xULN). ALP was high at 119 IU/L (ULN 104) as was GGT at 110 IU/L (ULN 42). On the same day, abdominal ultrasound scan was performed and showed normal appearance of liver. An area of calcification in the right lobe was suggestive of hepatic granuloma. Additionally, no solid focal lesion was seen. Hepatitis C, Hepatitis B, Hepatitis A, and Hepatitis E serologies were negative. ALT and AST values slowly returned to normal by D22 without treatment or medication, as well as ALP and GGT. Bilirubin remained within normal range throughout the study. Given the known safety profile of PYR in previous Phase 1 studies and temporal relationship to dosing, this was deemed drug related.

Narrative E
36 years of age/female; PYR+PQP 720/960 mg.
Asymptomatic elevated LFTs (hypertransaminasemia), severe, drug related, resolved.
Low platelet count (thrombocytopenia), moderate, drug related, resolved.
Asymptomatic elevation of transaminases starting at D4 (AST and ALT >5xULN). AST peaked at 215 IU/L D4 (6.94xULN) and ALT peaked at 285 IU/L D5 (8.14xULN). Values returned to normal without treatment or medication. Bilirubin remained within normal range throughout the study. Given the known safety profile of PYR in previous Phase 1 studies, and temporal relationship to dosing, this was deemed drug related. Gradual decrease in platelet count since dosing D1. Baseline value (Day −1) was 164 x 10^9/L. Lowest value on D8 (68 x 10^9/L), slowly recovering from then to within normal limits by D15 (182 x 10^9/L). Participant remained asymptomatic. Coagulation parameters were normal, whereas other hematology safety laboratory values showed a mild eosinophilia, a mild neutropenia confirmed with blood film test and a mild lymphopenia. Concomitant recovering transaminitis as described above. This case was discussed with a Consultant Haematologist. Given the associated eosinophilia, known side effect profile of PYR and temporal relationship to dosing, this was deemed drug related.

Table S2 Time course of lymphocyte values for individual participants with values below the lower limit of normal.

**Treatment group**	**Lymphocyte values (x10^9/L) at Day**								
	−1	4	5	6	7	8	15	22	30
PYR+PQP 540/960 mg	1.42	1.63	0.97	1.00	1.08	1.57	1.12	1.17	1.08
	1.95	2.31	1.15	1.30	1.71	1.83	1.80	1.74	1.80
	2.16	2.05	1.15	1.21	1.15	1.43	1.72	1.77	1.20
	1.82	1.35	1.01	1.08	1.19	1.62	1.84	1.35	1.74
PYR+PQP 720/960 mg	1.37	1.22	0.56	0.83	1.11	1.59	1.65	1.45	1.20
	1.29	1.46	1.01	1.02	0.96	1.22	1.29	1.03	0.92
	1.76	1.73	0.89	0.95	1.02	1.47	1.92	2.16	3.86
	1.21	1.00	0.40	0.53	0.40	0.70	1.09	1.07	0.94
PYR+PQP 720/1280 mg	1.80	1.53	1.23	1.14	1.30	1.69	1.63	1.49	1.46
	1.69	1.77	1.19	1.19	1.08	1.26	1.92	1.41	1.57
	1.04	0.97	0.52	0.79	0.73	0.89	1.51	1.29	1.16
	1.49	1.16	0.68	0.86	0.81	1.15	1.30	1.55	1.67
PYR 540 mg	1.07	1.32	0.67	0.8	0.96	1.09	1.27	1.30	1.32
	1.53	1.58	1.30	1.16	0.96	1.51	1.75	–	1.99
	1.71	1.31	0.51	0.51	0.74	0.73	1.24	1.41	1.30
PYR 720 mg	1.86	1.61	1.14	1.06	1.07	2.15	1.51	1.84	2.05
	1.19	1.43	0.66	1.06	0.95	1.17	1.03	1.12	1.25
	2.27	2.07	0.73	1.05	1.57	1.76	2.34	1.29	1.33
	2.14	1.77	0.73	0.91	0.65	1.58	1.81	2.27	1.75
	1.61	1.61	1.24	1.17	1.12	1.55	1.50	1.36	2.02
PYR 960 mg	1.55	1.55	1.64	–	1.57	1.42	1.69	1.18	2.09
PQP 1280 mg	1.36	1.59	1.28	1.15	1.06	1.02	1.67	0.89	1.03
Placebo	1.31	1.66	1.46	1.32	1.34	1.51	1.10	1.20	1.52
	1.36	1.26	1.52	1.49	1.17	1.08	1.83	2.22	1.71
	1.87	1.35	1.19	1.35	1.52	1.65	1.38	1.43	1.28

Values below the lower limit of normal (1.2 x10^9/L) are shaded.

Table S3 Time course of eosinophil % values for individual participants with values outside the limits of normal.

Treatment group	Eosinophils % values at Day									
	−1	1	4	5	6	7	8	15	22	30
PYR+PQP 540/960 mg	3.3	5.3	6.9	5.7	5.8	6.2	3.3	3.5	2.2	2.9
	1.7	1.9	2.5	3.0	2.2	1.5	0.9	1.0	0.9	1.5
	5.5	5.4	6.4	7.2	8.0	6.4	4.4	2.4	3.2	2.2
	0.9	1.2	3.1	3.2	3.0	2.2	2.1	0.7	0.8	0.9
PYR+PQP 720/960 mg	0.5	0.9	2.7	2.7	2.7	1.6	1.0	0.8	0.4	0.7
	2.3	2.7	6.5	5.5	6.5	5.4	3.9	2.6	1.4	2.7
	2.2	4.4	9.5	11.1	11.9	13.0	9.4	2.0	1.9	3.0
	4.8	10.2	10.2	14.4	10.7	11.8	9.0	7.8	4.5	8.8
PYR+PQP 720/1280 mg	2.4	1.9	2.6	3.0	3.6	1.9	0.9	0.3	4.9	5.1
	4.2	4.1	8.2	8.3	7.9	6.5	4.7	6.0	4.6	5.8
	3.7	5.1	6.3	6.2	7.5	6.7	3.4	5.3	2.9	5.7
	1.4	2.3	4.7	6.0	5.0	4.8	3.4	0.6	3.3	2.3
PYR 540 mg	0.6	1.2	3.1	3.8	3.7	4.0	1.7	–	0.6	1.3
PYR 720 mg	5.2	5.4	5.1	8.7	9.3	8.4	8.5	7.5	6.8	7.3
	6.4	5.0	7.1	7.1	6.5	5.8	3.5	4.0	4.6	4.7
	1.4	2.2	5.2	5.2	5.8	5.8	4.2	0.3	2.4	1.6
	1.2	1.8	2.2	3.7	3.2	3.6	2.4	1.8	1.3	0.2
	11.7	11.7	18.0	15.0	15.3	13.9	14.6	0	4.0	8.8
PQP 960 mg	0.9	1.7	1.6	–	2.6	2.6	1.7	3.5	5.9	2.7
	5.3	4.7	3.8	5.8	6.0	4.7	3.6	7.2	14.3	6.2
	0.2	1.5	2.3	2.2	1.6	1.7	2.3	0.8	0.4	1.0
PQP 1280 mg	8.4	11.4	5.6	7.6	6.5	8.2	4.0	9.4	6.9	11.2
	2.7	2.8	3.4	2.8	3.3	2.3	2.4	8.5	5.5	6.6
Placebo	1.1	0.8	1.4	1.6	1.6	1.9	0.9	1.1	0.7	0.6
	6.3	7.8	10.4	9.6	11.2	9.2	8.5	7.0	9.7	4.9
	0.6	1.0	2.0	1.9	2.2	1.4	1.2	1.0	0.9	0.7

Values above the upper limit of normal (6.0%) are shaded lighter and below the lower limit of normal (1.0%) are shaded darker.

Figure S4 Changes in blood pressure.

Values are mean ± standard deviation.

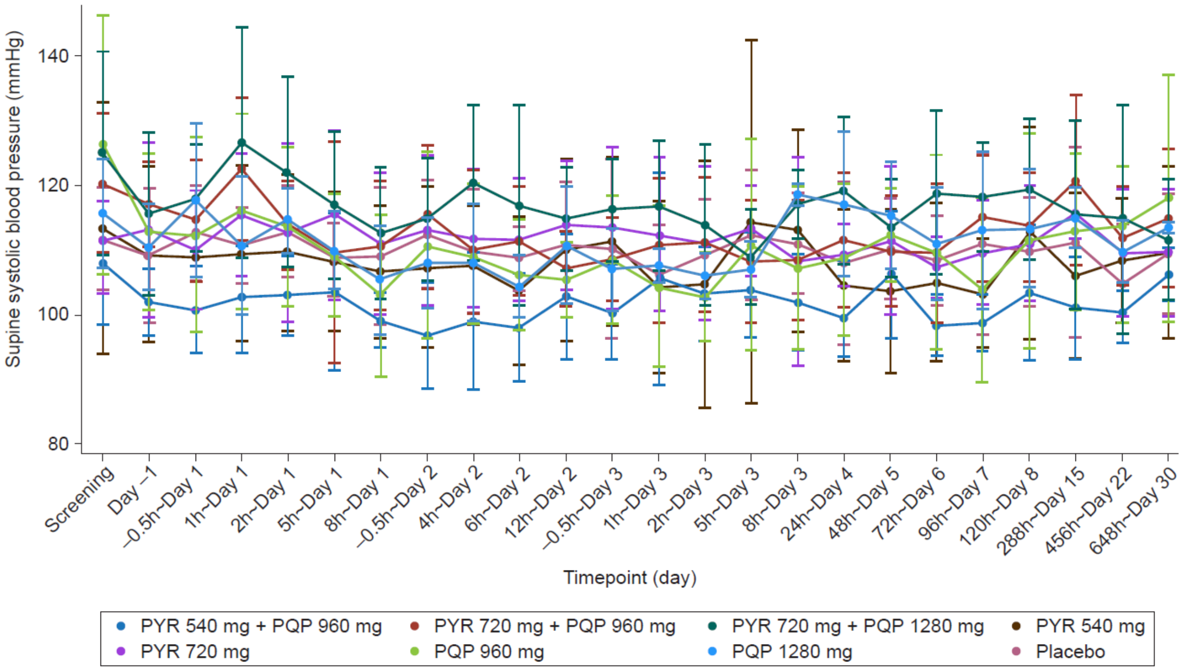

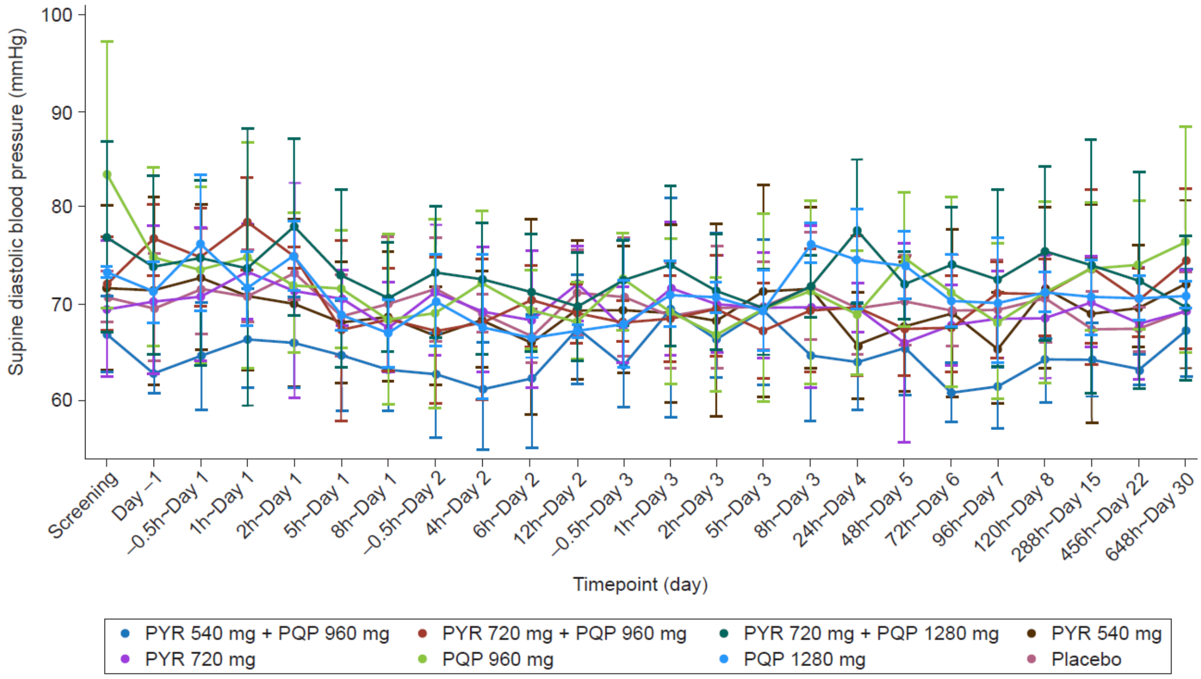

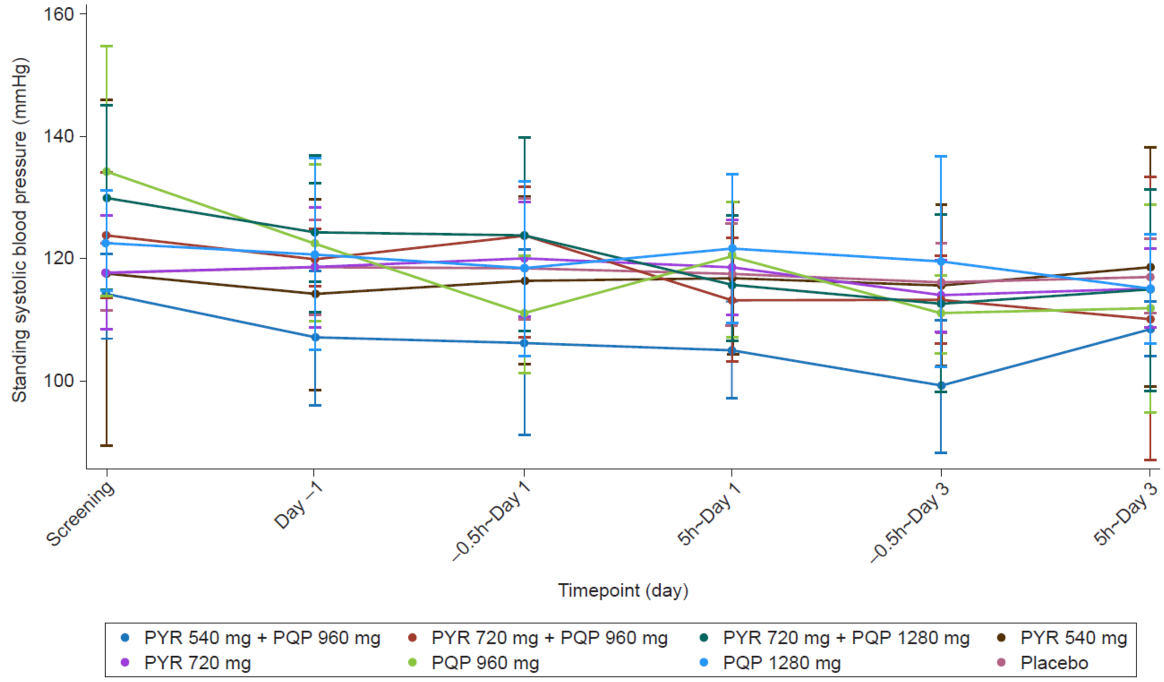
160
140
120
100
Standing systolic blood pressure (mmHg)
Screening
Day –1
–0.5h–Day 1
5h–Day 1
–0.5h–Day 3
5h–Day 3
Timepoint (day)
PYR 540 mg + PQP 960 mg
PYR 720 mg + PQP 960 mg
PYR 720 mg + PQP 1280 mg
PYR 540 mg
PYR 720 mg
PQP 960 mg
PQP 1280 mg
Placebo

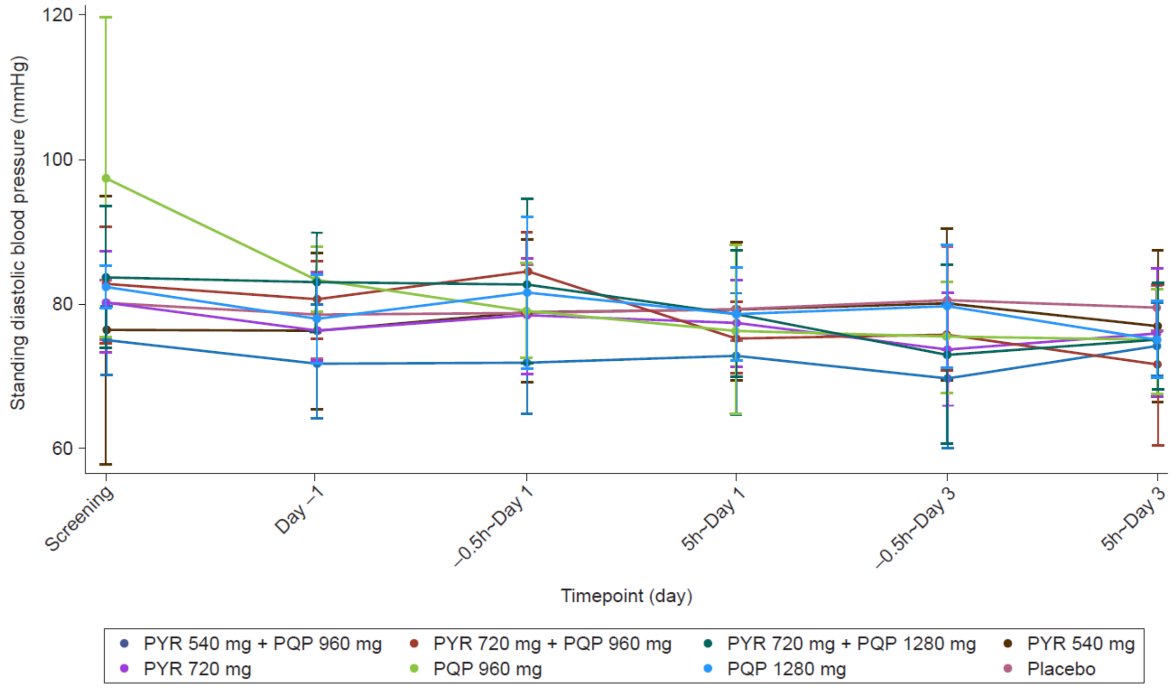
120
100
80
60
Standing diastolic blood pressure (mmHg)
Screening
Day –1
–0.5h–Day 1
5h–Day 1
–0.5h–Day 3
5h–Day 3
Timepoint (day)
PYR 540 mg + PQP 960 mg
PYR 720 mg + PQP 960 mg
PYR 720 mg + PQP 1280 mg
PYR 540 mg
PYR 720 mg
PQP 960 mg
PQP 1280 mg
Placebo

Table S4 Post-baseline categorical data for Fridericia-corrected QT interval (QTcF) change from baseline >30 msec and absolute values >450 msec.

QTcF >30 msec change from baseline	Time	PYR+PQP dose groups (mg)				PYR+placebo dose groups (mg)			PQP+placebo dose groups (mg)			Placebo (N=6)
		All doses (N=15)	540/960 (n=5)	720/960 (n=5)	720/1280 (n=5)	All doses (N=8)	540 (n=3)	720 (n=5)	All doses (N=8)	960 (n=5)	1280 (n=3)	
Day 1	1 h	0	0	0	0	0	0	0	0	0	0	0
	2 h	0	0	0	0	0	0	0	0	0	0	0
	3 h	1	0	1	0	1	0	1	0	0	0	0
	4 h	2	0	1	1	0	0	0	0	0	0	0
	5 h	1	1	0	0	0	0	0	1	1	0	0
	6 h	1	0	0	1	0	0	0	0	0	0	0
	7 h	0	0	0	0	1	1	0	0	0	0	0
	8 h	0	0	0	0	0	0	0	0	0	0	0
	12 h	1	0	0	1	0	0	0	0	0	0	0
Day 2	−1.5 h	0	0	0	0	0	0	0	0	0	0	0
	−1 h	0	0	0	0	0	0	0	0	0	0	0
	−0.5 h	1	0	0	1	0	0	0	0	0	0	0
	4 h	5	1	2	2	1	0	1	2	2	0	0
	6 h	0	0	0	0	0	0	0	0	0	0	0
	12 h	2	0	1	1	0	0	0	1	1	0	0
Day 3	−1.5 h	0	0	0	0	0	0	0	1	1	0	0
	−1 h	1	0	0	1	0	0	0	0	0	0	0
	−0.5 h	1	0	1	0	0	0	0	1	1	0	0
	−5 min	1	0	0	1	0	0	0	1	1	0	0
	1 h	2	0	1	1	0	0	0	0	0	0	0
	2 h	3	1	1	1	0	0	0	1	1	0	1
	3 h	6	1	3	2	0	0	0	2	2	0	0
	4 h	8	2	4	2	0	0	0	2	2	0	0
	5 h	2	1	1	0	0	0	0	1	1	0	0
	6 h	2	0	1	1	0	0	0	0	0	0	0
	7 h	1	1	0	0	0	0	0	0	0	0	0

	8 h	1	0	0	1	0	0	0	1	1	0	0
	12 h	1	0	1	0	0	0	0	0	0	0	0
Day 4	24 h	0	0	0	0	0	0	0	0	0	0	0
Day 5	48 h	1	0	1	0	0	0	0	0	0	0	0
Day 6	72 h	1	0	1	0	0	0	0	0	0	0	0
Day 7	96 h	0	0	0	0	0	0	0	0	0	0	0
Day 8	120 h	0	0	0	0	0	0	0	0	0	0	0
Day 15	288 h	0	0	0	0	0	0	0	0	0	0	0
Day 22	456 h	0	0	0	0	0	0	0	0	0	0	0
Day 30	648 h	0	0	0	0	0	0	0	0	0	0	0
Observations, n/N		45/540	8/180	20/180	17/180	3/288	1/108	2/180	14/288	14/180	0/108	1/216
Observations, %		8.3	4.4	11.1	9.4	1.0	0.9	1.1	4.9	7.8	0.0	0.5

**QTcF >450 msec**	**Time**	**PYR+PQP dose groups (mg)**				**PYR+placebo dose groups (mg)**			**PQP+placebo dose groups (mg)**			**Placebo (N=6)**
		**All doses (N=15)**	**540/960 (n=5)**	**720/960 (n=5)**	**720/1280 (n=5)**	**All doses (N=8)**	**540 (n=3)**	**720 (n=5)**	**All doses (N=8)**	**960 (n=5)**	**1280 (n=3)**	
Day 1	1 h	0	0	0	0	0	0	0	0	0	0	0
	2 h	0	0	0	0	0	0	0	0	0	0	0
	3 h	0	0	0	0	0	0	0	0	0	0	0
	4 h	0	0	0	0	0	0	0	0	0	0	0
	5 h	0	0	0	0	0	0	0	0	0	0	0
	6 h	0	0	0	0	0	0	0	0	0	0	0
	7 h	0	0	0	0	0	0	0	0	0	0	0
	8 h	0	0	0	0	0	0	0	0	0	0	0
	12 h	0	0	0	0	0	0	0	0	0	0	0
Day 2	−1.5 h	0	0	0	0	0	0	0	0	0	0	0
	−1 h	0	0	0	0	0	0	0	0	0	0	0
	−0.5 h	0	0	0	0	0	0	0	0	0	0	0
	4 h	0	0	0	0	0	0	0	2	1	1	0
	6 h	0	0	0	0	0	0	0	0	0	0	0
	12 h	0	0	0	0	0	0	0	0	0	0	0
Day 3	−1.5 h	0	0	0	0	0	0	0	1	0	1	0

	−1 h	0	0	0	0	0	0	0	1	0	1	0
	−0.5 h	0	0	0	0	0	0	0	1	0	1	0
	−5 Min	0	0	0	0	0	0	0	1	0	1	0
	1 h	0	0	0	0	0	0	0	1	0	1	0
	2 h	0	0	0	0	0	0	0	1	0	1	0
	3 h	0	0	0	0	0	0	0	2	1	1	0
	4 h	2	2	0	0	0	0	0	1	0	1	0
	5 h	0	0	0	0	0	0	0	1	0	1	0
	6 h	0	0	0	0	0	0	0	0	0	0	0
	7 h	0	0	0	0	0	0	0	0	0	0	0
	8 h	0	0	0	0	0	0	0	1	0	1	0
	12 h	0	0	0	0	0	0	0	0	0	0	0
Day 4	24 h	0	0	0	0	0	0	0	1	0	1	0
Day 5	48 h	0	0	0	0	0	0	0	0	0	0	0
Day 6	72 h	0	0	0	0	0	0	0	1	0	1	0
Day 7	96 h	0	0	0	0	0	0	0	0	0	0	0
Day 8	120 h	0	0	0	0	0	0	0	0	0	0	0
Day 15	288 h	0	0	0	0	0	0	0	0	0	0	0
Day 22	456 h	0	0	0	0	0	0	0	1	0	1	0
Day 30	648 h	0	0	0	0	0	0	0	0	0	0	0
Observations, n/N		2/540	2/180	0/180	0/180	0/288	0/108	0/180	16/288	2/180	14/108	0/216
Observations, %		0.4	1.1	0.0	0.0	0.0	0.0	0.0	5.6	1.1	13.0	0.0

PYR, pyronaridine; PQP, piperaquine; QTcF, Fridericia-corrected QT. Values are number of participants.

There were no absolute QTcF values >480 msec or changes in QTcF from baseline >60 msec at any time point.

Table S5 Fold changes comparing pyronaridine (PYR) exposure following PYR+placebo (n=8) with PYR exposure following PYR+piperaquine (PQP) coadministration.

Parameter	Time	All doses (n=15)	PYR+PQP dose groups (mg) 540/960 (n=5)	720/960 (n=5)	720/1280 (n=5)
C_{max} (ng/mL)	Day 1	1.2	1.0	1.3	1.2
	Day 2	1.5	1.5	1.7	1.3
	Day 3	1.3	1.3	1.4	1.3
$AUC_{0\text{-}t}$ (ng.h/mL)	Day 1	1.3	1.3	1.5	1.1
	Day 2	1.6	1.7	1.7	1.3
	Day 3	1.6	1.6	1.7	1.5
$AUC_{0\text{-}24h}$ (ng.h/mL)	Day 1	1.4	1.5	1.6	1.1
	Day 3	1.5	1.6	1.6	1.3
$AUC_{0\text{-}168h}$ (ng.h/mL)	Day 1	1.6	2.1	1.6	1.1
	Day 3	1.7	1.7	1.8	1.5
$AUC_{0\text{-}inf}$ (ng.h/mL)	Day 1	1.3	1.4	1.5	1.2
	Day 3	1.4	1.5	1.4	1.2
AUC_{tau} (ng.h/mL)	Day 3	1.5	1.6	1.6	1.3

PYR, pyronaridine; PQP, piperaquine; C_{max}, maximum observed plasma concentration; AUC, area under the plasma concentration time curve (AUC); $AUC_{0\text{-}t}$, AUC from time 0 to last detectable plasma concentration; $AUC_{0\text{-}inf}$, AUC from time 0 extrapolated to infinity; AUC_{tau}, AUC from time zero until the end of the dosing interval.

Table S6 Fold changes comparing piperaquine (PQP) exposure following PQP+placebo (n=8) with PQP exposure following pyronaridine (PYR)+PQP coadministration.

Parameter	Time	All doses	PYR+PQP dose groups (mg)		
			540/960 (n=5)	720/960 (n=5)	720/1280 (n=5)
C_{max} (ng/mL)	Day 1	2.7	3.0	2.5	2.6
	Day 2	1.2	1.3	0.9	1.5
	Day 3	1.4	1.2	1.1	1.7
AUC_{0-t} (ng.h/mL)	Day 1	2.2	2.4	2.2	2.0
	Day 2	1.2	1.3	1.0	1.4
	Day 3	1.0	1.0	1.0	1.1
AUC_{0-24h} (ng.h/mL)	Day 1	1.8	2.1	1.9	1.5
	Day 3	1.2	1.2	1.2	1.2
AUC_{0-168h} (ng.h/mL)	Day 1	2.0	2.8	1.8	1.6
	Day 3	1.0	1.0	1.0	1.0
AUC_{0-inf} (ng.h/mL)	Day 1	1.8	1.6	2.8	1.4
	Day 3	0.7	0.6	0.8	NC
AUC_{tau} (ng.h/mL)	Day 3	1.2	1.2	1.2	1.2

PYR, pyronaridine; PQP, piperaquine; C_{max}, maximum observed plasma concentration; AUC, area under the plasma concentration time curve (AUC); AUC_{0-t}, AUC from time 0 to last detectable plasma concentration; AUC_{0-inf}, AUC from time 0 extrapolated to infinity; AUC_{tau}, AUC from time zero until the end of the dosing interval. NC, not calculable.
